# Metabolic Responses of Newly Isolated Microalgal Strains Cultured in an Open Pond Simulating Reactor Under Balanced Conditions and Nutrient Limitation

**DOI:** 10.3390/life15091427

**Published:** 2025-09-11

**Authors:** Panagiotis Dritsas, George Aggelis

**Affiliations:** Department of Biology, School of Natural Sciences, University of Patras, 26500 Patras, Greece; dritsas.p@ac.upatras.gr

**Keywords:** *Picochlorum*, *Microchloropsis*, *Nephroselmis*, aquaculture, lipids

## Abstract

Microalgal strains—*Picochlorum costavermella* VAS2.5, *Picochlorum oklahomense* PAT3.2B and SAG4.4, *Microchloropsis gaditana* VON5.3, and *Nephroselmis pyriformis* PAT2.7—were evaluated in an Open Pond Simulating Reactor (OPSR) under varied conditions to assess their biomass yield and high-value metabolite production. Overall, the strains produced 269.1–523.0 mg/L of biomass under balanced growth conditions in modified Artificial Seawater, continuous illumination, and pH 8.5. Phosphorus limitation notably enhanced yields for SAG4.4 and PAT2.7 (529.0 ± 52.2 mg/L and 452.2 ± 21.0 mg/L, respectively). Conversely, nitrogen limitation reduced productivity. In most strains the glycolipid plus sphingolipid fraction was dominant. Significant quantities of 20:5(n-3) were traced in the cultures of VAS2.5 and VON5.3, while the PAT3.2B and SAG4.4 strains produced considerable amounts of 18:3(n-3). In contrast, the most interesting fatty acid synthesized by PAT2.7 was 16:1(n-7), which was also detected in significant quantities in VAS2.5 and VON5.3. Polysaccharide content remained stable across conditions (10–15%), and protein levels reached 45–50% under control and phosphorus-limited environments. Pigment synthesis peaked at control conditions. Overall, the biochemical profiles of these strains revealed their potential for use primarily as feed additives in the aquaculture sector.

## 1. Introduction

Microalgae are photosynthetic microorganisms found in all aquatic environments worldwide [1,2,3,4,5]. A key and ecologically significant characteristic of microalgae is their capacity for photosynthesis, accounting for approximately 50% of global atmospheric oxygen production. Importantly, under optimal culture conditions, microalgae can generate considerable amounts of high-value metabolites.

Genera like *Picochlorum*, *Microchloropsis*, and *Nephroselmis* have emerged as promising candidates for various biotechnological applications, due to their higher growth rates compared with commercial strains [6,7,8,9,10,11]. *Picochlorum* and *Microchloropsis* produce lipids with significant amounts of PUFAs, i.e., α-linolenic acid (ALA, 18:3(n-3)) and/or eicosapentaenoic acid (EPA, 20:5(n-3)) [8,11,12,13,14,15], which have been associated, among others, with the prevention of cardiovascular and neurodegenerative diseases [16,17,18]. *Nephroselmis* strains tend to synthesize more saturated lipids, which are more suitable for biodiesel manufacturing [8,13,19,20,21]. Nonetheless, such microalgae are considered to be important producers of proteins, providing essential amino acids to fish when used as aquafeeds, either directly or indirectly (through zooplankton feeding), and sugars [13,21,22]. Also, their pigment content, particularly carotenoids like lutein, beta-carotene, and zeaxanthin, is of high interest to various industrial sectors, e.g., food, feed, paints, etc. [6,14,23,24].

Regardless, the physiology and yields of microalgae depend on a plethora of abiotic and biotic parameters, such as the availability of nutrients (e.g., CO_2_, N, P, and trace elements), pH, light intensity, and photoperiod [25,26,27,28]. For example, the abundance of N, P, and minerals is critical for biomass production, whereas nutrient limitation can enhance lipid biosynthesis. Light availability and duration are also critical parameters, with insufficient intensity or photoperiod acting as limiting factors for microalgal growth. Additionally, pH is a very important parameter, since the uptake of nutrients (including CO_2_, N, and P) is heavily related to it. However, it must be highlighted that optimal conditions are species- or even strain-dependent.

The aim of the present study was the biochemical characterization of recently isolated [20] microalgal strains, i.e., *Picochlorum costavermella* VAS2.5, *Picochlorum oklahomense* SAG4.4, *Picochlorum oklahomense* PAT3.2B, *Microchloropsis gaditana* VON5.3, and *Nephroselmis pyriformis* PAT2.7, when cultured in an Open Pond Simulating Reactor (OPSR) containing a balanced growth medium (i.e., modified Artificial Seawater) or nitrogen- or phosphorus-limited media. An OPSR is a cost-effective and scalable type of photobioreactor which mimics large-scale photobioreactors that are often used for microalgal culture and offers the possibility to monitor and control various environmental variables, including light intensity, pH, and water temperature. The selection of the above microalgal strains was based on their interesting biochemical profiles, as showcased in previous studies [8,13,20], and due to the limited literature, especially for *Picochlorum* and *Nephroselmis*, regarding their physiology and their potential use in various biotechnological applications. Moreover, since these strains are indigenous, thus well adapted to the Mediterranean climate, they are expected to be cultured more efficiently and cost-effectively under such environmental conditions, challenging commercial strains. The selection of culture conditions was based on the goal of comparing the physiology of the selected microalgal strains under (a) balanced growth and (b) growth-limiting conditions, which presumably favor reserve material accumulation.

## 2. Materials and Methods

### 2.1. Biological Material

The green microalgal strains *Picochlorum costavermella* VAS2.5, *Picochlorum oklahomense* PAT3.2B, *Picochlorum oklahomense* SAG4.4, *Microchloropsis* (formerly known as *Nannochloropsis*) *gaditana* VON5.3, and *Nephroselmis pyriformis* PAT2.7 were used as biological material. These strains were previously collected from coastal areas of the Ionian Sea of Greece, treated in the laboratory until mono-algal but non-axenic using strain isolation, and identified by the use of molecular techniques, specifically by the PCR amplification of the 18S rRNA and ITS (internal transcribed spacer) gene markers [20]. Both the strain isolation process and molecular characterization are described in detail in Dritsas et al. (2023) [20].

### 2.2. Culture Conditions

**Figure 1:**
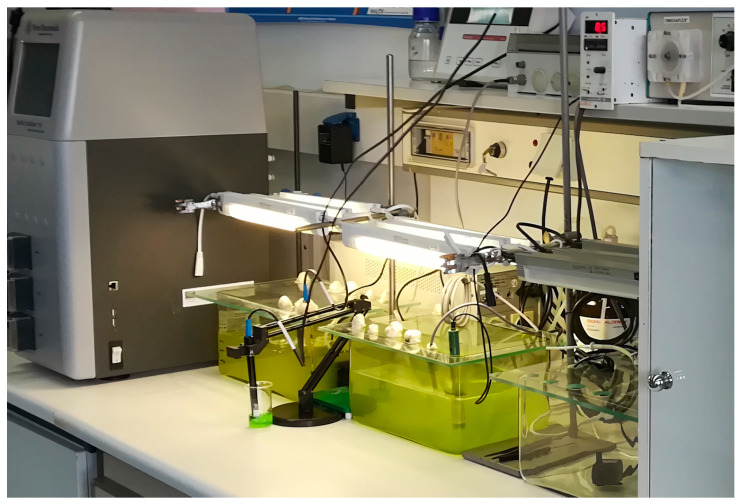
Open Pond Simulating Reactor (OPSR) with a capacity of 8.7 L (V_w_ = 5 L) used for the culture of selected microalgae strains.

The microalgal strains were cultured in an 8.7 L (V_w_ = 5 L) Open Pond Simulating Reactor (OPSR) (Figure 1). The OPSR was used for cultures under the different examined environmental conditions as a convenient and affordable type of bioreactor for lab-scale use. In detail, the dimensions of the OPSR were 29 cm × 20 cm × 15 cm (length × width × height). Transparent glass of 5 mm thickness was placed on the surface of the pond to minimize the evaporation of the growth medium and to prevent possible invasion by predators or suspended particles.

Pre-cultures were performed at a scaled level, initially from 0.25 L Erlenmeyer flasks (V_w_ = 0.1 L) to 1 L Erlenmeyer flasks (V_w_ = 0.5 L) prior to the inoculation of the cultures at 5 L with 0.2–0.5 L of microalgal pre-culture. The initial culture concentration was 1.5·10^6^ cells/mL. The basal growth medium was modified Artificial Seawater (mASW) (Table 1), which was used for the maintenance of microalgae, as well as growth medium in the control experiments, i.e., balanced growth conditions. The growth medium was sterilized in an autoclave (Raypa AES-75, Madrid, Spain) at 121 °C for 20 min under 1.1 atm. It is noted that prior to the addition of the growth medium (4.5–4.8 L) to the OPSR, the disinfection of the pond with 70% ethanol (vol/vol) occurred.

As depicted in Figure 1, three Τ5, 8 W, 6500 Κ fluorescent lamps placed above the surface of the culture provided a continuous illumination of 232 μE/m^2^·s. Preliminary experiments were also carried out under the same light intensity, but with the application of a 16:8 (h, light/dark) photoperiod (see Supplementary Material). Except for the practical restrictions of the used set-ups, the selection of the aforementioned light intensity was based on the fact that most green microalgae thrive within the range of 50–400 μE/m^2^·s for balanced growth and photosynthetic activity.

Stirring was carried out using an Astro Liquid Filter 300 (Ace Story Aquatic, Butterworth Penang, Malaysia) water circulator with a flow rate of 150 L/h, from the inlet of which natural, moisture-saturated air was supplied to the culture at a rate of 0.5 vvm corresponding to an air flow rate of 150 L/h. Cultures were carried out in a room with a temperature of 25 ± 1 °C. During the experiments, temperature and pH values were monitored using an electronic thermometer and a pH electrode (EYELA—Model FC-10, Tokyo Rikakikai Co. Ltd., Tokyo, Japan) and adjusted accordingly, with the addition of NaOH 2.5 M or HCl 2.5 M to 8.5 ± 0.3 or 7.5 ± 0.3, in the case of the respective cultures that were carried out in preliminary experiments (see Supplementary Material), when needed. Samples were harvested on a daily basis to estimate population growth by cell number enumeration, and at selected time points (approximately at 240 h and 450 h of culture), culture volumes of 1.5 L were harvested to determine dry biomass and various metabolic products.

The strains were cultured under balanced conditions (i.e., in mASW, having a ratio of N:P = 19:1, at pH 8.5, with continuous illumination). In addition, these strains were subjected to nitrogen- or phosphorus-limited conditions. For nitrogen-limited conditions, KNO_3_ was added in concentrations of 0.1 g/L, employed for the strains of the genera *Picochlorum* and *Nephroselmis*, or 0.4 g/L, employed only for *M. gaditana* VON5.3. The above means that N:P was equal to ~2:1 and ~8:1 when KNO_3_ was 0.1 g/L and 0.4 g/L, respectively; thus both growth media were considered as being strongly nitrogen-limited, taking into consideration that according to Redfield, a N:P ratio in the range of 16:1 offers balanced growth conditions for microalgae. Despite the lack of data in the literature regarding the ideal N:P ratio for enhanced lipid accumulation in *Picochlorum* and *Nephroselmis*, it was taken into consideration the knowledge that N:P in the range of 2:1 to 8:1 supports high lipid accumulation. Additionally, following the findings in a previous study [13] regarding NO_3_^−^-N (%) uptake when these strains were cultured in an Open Pond in mASW for 19 days, the conditions ranged from 14.8 to 29.9% of the initial KNO_3_ = 1 g/L. On the other hand, regarding *M. gaditana* VON5.3, the design of the culture in the selected N:P ratio was in accordance with evidence from the literature which presents efficacy in enhancing lipid accumulation. For phosphorus-limited treatments, KH_2_PO_4_ was added at 0.02 g/L instead of 0.07 g/L, i.e., a ratio of N:P of 67:1, establishing a strongly limited growth medium in phosphorus, as ratios in the range 40–80:1 are often used to study lipid accumulation or stress physiology. Moreover, in the previously mentioned study [13] the PO_4_^3−^ (%) uptake of the strains used herein ranged from 60.4 to 80% of the initial KH_2_PO_4_ = 0.07 g/L. All other parameters remained consistent with the control set-up.

### 2.3. Cell Growth and Biomass Determination

The growth of microalgae was determined by the use of a Neubauer improved cell counting chamber (Poly-Optik, Bad Blankenburg, Germany) on a daily basis. This method was preferred as it offers cell count visually, thus avoiding interference from other materials, e.g., salts, cell debris, etc., which would risk the accuracy of optical density measurements. The cell counts were expressed as microalgal cell density (cells/mL) and the integrated version of Verhulst’s model (i.e., Sigmoidal Logistic function) (1) was employed as follows to estimate the parameters of growth:

*N*(*t*) = *N_max_*/1 + *b*·e^1/^*^μ·t^* (1)

where *N* denotes the cell concentration (×10^6^ cells/mL) at time *t*, *b* is a positive constant defined as (*N_max_* − *N*_0_)/*N*_0_, with *N*_0_ representing the initial cell concentration, *μ* is the maximum specific growth rate (1/d), and *N_max_* is the carrying capacity of the system. Parameter values estimation was performed by fitting Equation (1) to the experimental data using the Levenberg–Marquardt optimization method. Model fit quality was evaluated by minimizing the residual root mean square error between the experimental and model-predicted data, quantified by the values of the coefficient of determination R^2^, which was used as a criterion for parameter optimization.

Biomass was quantified gravimetrically at designated culture time points: approximately 240 h and 450 h. Microalgal cells were harvested by centrifugation (NÜVE NF 800R, Ankara, Turkey) at 7455× *g* for 10 min at 4 °C, washed twice with deionized water, and dried at 80 °C until a constant weight was achieved. Biomass values were expressed as g/L.

### 2.4. Lipid Extraction and Purification

Total microalgal lipids were extracted using a 2:1 (vol/vol) mixture of chloroform (PENTA)/methanol (Fisher Chemical, Hampton, VA, USA), in accordance with a modified version of the Folch et al. (1957) [29] method, as adapted by Dourou et al. (2018) [30]. The purification of the obtained lipids from non-lipid components occurred as mentioned elsewhere [8]. The final solvent was evaporated under vacuum (Rotavapor R-210 evaporator—BUCHI, Flawil, Switzerland), allowing the gravimetric determination of total cellular lipids, expressed as a percentage of dry biomass (L/x%, wt/wt).

### 2.5. Lipid Fractionation

Microalgal lipids (~100 mg) were dissolved in 1 mL of chloroform and subjected to fractionation. The column used was of dimensions 25 × 100 mm, packed with 1 g of silicic acid (Fluka). Previously, silicic acid had been activated by heating at 80 °C overnight. Sequential elution with different solvents occurred for obtaining the fractions of neutral lipids (N), glycolipids and sphingolipids (G + S), and phospholipids (P), as described in detail elsewhere [31]. After solvent evaporation under vacuum, the lipid fractions were quantified gravimetrically and expressed as a percentage of the total lipids.

### 2.6. Fatty Acid Composition of Cellular Lipids

Total lipids and their fractions were converted into fatty acid methyl esters (FAMEs) and analyzed using an Agilent 7890A Gas Chromatography (GC) system (Agilent Technologies, Shanghai, China) to determine their fatty acid composition, as described in detail in the study of Dritsas & Aggelis [8].

### 2.7. Polysaccharide Determination

Lipid-free biomass (*x_f_*), in the range of 20–25 mg, was hydrolyzed with 5 mL HCl 2.5 M at 100 °C for 60 min. Upon the cooling of the sample at ambient temperature, the hydrolysate was neutralized with KOH 2.5 M and filtered through Whatman No. 1 paper to remove cell debris. The reducing sugars, expressed as glucose, were quantified using the 3,5-dinitrosalicylic acid (DNS) method [32]. Intracellular polysaccharides, i.e., both storage and structural polysaccharides, were quantified as glucose equivalents and reported as a percentage of dry biomass (S/x%, wt/wt).

### 2.8. Protein Determination

Cellular protein was quantified from 10 to 15 mg of *x_f_* using the biuret assay, as albumin equivalents [31], and expressed as a percentage of dry biomass (P/x%, wt/wt).

### 2.9. Pigment Estimation

Approximately 0.5 g of wet biomass was solved in 10 mL of ethanol 95% (vol/vol) (Fisher Chemical), prepared with Milli Q water (Honeywell, Charlotte, NC, USA). Following this, centrifugation (Hettich Mikro 200R, Föhrenstr, Germany) of the mixture at 13,300× *g* for 15 min at 4 °C occurred. Subsequently, 0.5 mL of the resulting supernatant was mixed with 4.5 mL of the ethanol solution [33] and spectrophotometric analysis for Chlorophyll-a, Chlorophyll-b, and carotenoids in a 1 cm^2^ quartz cuvette occurred. The above-mentioned ethanol solution was used as the blank.

Equations (2)–(4) were used for the pigment quantification (in µg/mL) as follows [33]:

*C_a_* = (13.36·*A*_664_) − (5.19·*A*_649_) (2)

*C_b_* = (27.43·*A*_649_) − (8.12·*A*_664_) (3)

*C_x+c_* = ((1000·*A*_470_) − (2.13·*Ch_a_*) − (97.63·*Ch_b_*))/209 (4)

where *A* represents absorbance and *Ch_a_*, *Ch_b_*, and *C_(exec)_* stand for chlorophyll-a, chlorophyll-b, and carotenoids, respectively. Pigments, evaluated as total chlorophylls (TCh) and total carotenoids (TC), were expressed as percentage in the dry biomass (TCh/x%, wt/wt, and TC/x%, wt/wt). It is noted that, in contrast to *Picochlora* strains and *N. pyriformis* PAT2.7, *M. gaditana* VON5.3 contains only chlorophyll-a [34]; thus the results provided in the following section refer only to chlorophyll-a and not the sum of chlorophyll-a and chlorophyll-b.

### 2.10. Data Treatment and Statistical Analysis

The treatment of the experimental data from the microalgal cultures and growth kinetics was carried out with the use of the graphing and analysis software OriginPro 2021 9.8.0.200 ^®^, 1991–2020 (OriginLab Corp., Northampton, MA, USA).

Additionally, all experimental sets in the Tables were performed in duplicate from two biological replicates and the results are presented as mean values ± standard deviation (SD). A two-sample *t*-test was applied for the statistical analysis of the values regarding biomass production and reserve materials accumulation (i.e., lipids, polysaccharides, and proteins) obtained for a culture at 240 h and 450 h of culture. Statistically significant differences in biomass production, reserve materials accumulation, and the fatty acid composition of total lipids among the various culture conditions at 240 h and 450 h of culture were analyzed using a one-way ANOVA analysis of variance. Tukey’s Honest Significant Difference (HSD) post hoc test was applied to identify pairwise differences between group means. A value of *p* ≤ 0.05 was considered statistically significant. It is noted that the results of the statistical analyses are presented in detail in the Supplementary Material.

## 3. Results

The growth curves of the five examined microalgal strains, namely *P. costavermella* VAS2.5, *P. oklahomense* PAT3.2B, *P. oklahomense* SAG4.4, *M. gaditana* VON5.3, and *N. pyriformis* PAT2.7, under balanced growth or nutrient-limited conditions are presented in the following paragraphs of this section.

Initially, the growth performance of the five strains was assessed under balanced growth conditions, i.e., without nitrogen nor phosphorus limitation, after their culture in mASW at pH 8.5 with a 24:0 photoperiod (h light/dark).

Considering that the optimal pH for green microalgae growth typically falls within the range of 7.0 and 8.5, depending on the species and culture conditions [9,35,36], a pH value of 7.5 was additionally tested in preliminary experiments (Figure S1).

On the other hand, even though some microalgal species show increased growth and biomass productivity under continuous illumination, a 24:0 photoperiod is not always the most suitable for green microalgae, while the application of a 16:8 photoperiod is quite favorable for the growth and physiology of many green microalgae species, including ones that belong to genera like *Picochlorum* and *Microchloropsis*, which presented sufficient growth and reserve material accumulation rates [37,38,39,40]. In addition, the potential for culturing microalgal strains efficiently under a 16:8 illumination regime is of high importance for reducing electricity costs in indoor set-ups. Moreover, it could be highly useful even for examining the possibility of further using these strains in larger-scale and outdoor set-ups. Considering the aforementioned, a 16:8 photoperiod was additionally tested in preliminary experiments (Figure S2).

**Figure 2:**
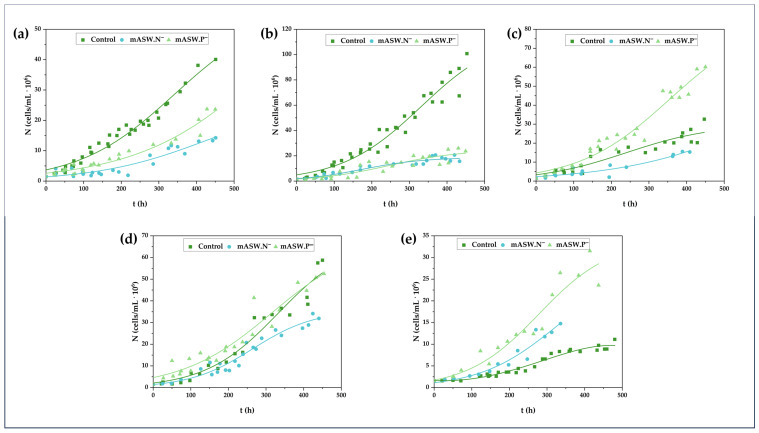
Growth curves of (**a**) *Picochlorum costavermella* VAS2.5, (**b**) *Picochlorum oklahomense* PAT3.2B, (**c**) *Picochlorum oklahomense* SAG4.4, (**d**) *Microchloropsis gaditana* VON5.3, (**e**) *Nephroselmis pyriformis* PAT2.7 cultured in modified Artificial Seawater (mASW) (control) and other variations in the growth medium under nutrient limitation in an Open Pond Simulating Reactor (OPSR) of 8.7 L (V_w_ = 5 L) capacity (two biological replicates). Each point is an average of two measurements and the curves were obtained by fitting the Verhulst model to the experimental data. In the legend of each graph the different culture conditions are denoted.

According to the retrieved data (see Supplementary Material, Tables S1–S10), in general, the highest yields in terms of biomass production and lipid, polysaccharide, and protein content were recorded for the cultures in mASW at pH = 8.5 and a photoperiod of 24:0. From now on, these culture conditions will be summarized under the term “control”. Therefore, the control was selected as the base to further explore the ability of the five microalgal strains to grow and synthesize high value-added metabolites under nitrogen- (mASW.N^−^) or phosphorus- (mASW.P^−^) limiting conditions (Figure 2).

### 3.1. Cell Growth and Biomass Production

The growth curves of *P. costavermella* VAS2.5 under the control and the different nutrient-limited culture conditions are presented in Figure 2a. *P. costavermella* VAS2.5 grew well under control conditions, gaining a biomass of 269.1 ± 6.7 mg/L. Despite the significant differences in cell number at the end of the cultures of this strain (Figure 2a), biomass production was slightly lower in mASW.N^−^ (x = 245.6 ± 8.9 mg/L) and mASW.P^−^ (x = 201.6 ± 0.4 mg/L) (Table 2a). Statistically significant differences in biomass production between 240 h and 450 h of culture were observed in the cases of the control (*p* = 0.007) and mASW.N^−^ (*p* = 0.002) experiments. Moreover, statistically significant differences in biomass production were recorded between the sets mASW.N^−^—mASW.P^−^ at 240 h (i.e., *p* = 0.037), and the set control—mASW.P^−^ (*p* = 0.010) and mASW.N^−^—mASW.P^−^ (*p* = 0.034)—at 450 h of culture.

The control conditions proved more suitable for the cell growth of *P. oklahomense* PAT3.2B as well (Figure 2b), though, cell growth herein was markedly higher (approximately 4–6 times) compared with the rest of the examined conditions (Figure 2b). Statistically significant differences in the biomass production of each culture between 240 h and 450 h were observed. Nonetheless, the highest biomass production, significantly different from the others, was recorded in the microalgal growth in the control medium (x = 421.1 ± 30.8 mg/L with), which was confirmed by the fact that the *p* values in the set control—mASW.N^−^ and control—mASW.P^−^ were 0.027 and 0.022, respectively, at 240 h of culture and 0.002 and 0.003, respectively, at 450 h. On the other hand, the final biomass produced in nutrient-limited conditions ranged from 98.0 to 125.3 mg/L (Table 3a). No statistically significant differences were observed in biomass production between the two nutrient-limited conditions.

The growth curves of *P. oklahomense* SAG4.4 under the control and nutrient-limited conditions are presented in Figure 2c. Notably, the growth data of culture mASW.N^−^ also applied to the linear model (linear correlation coefficient R^2^ = 0.87). Regarding this strain, like PAT3.2B, statistically significant differences in the biomass production of each culture between 240 h and 450 h were observed. Intriguingly, and in contrast to what was recorded for the other two *Picochlora*, this strain presented its highest biomass production under phosphorus-limiting conditions, i.e., x = 529.0 ± 52.2 mg/L, which was statistically significantly higher to what was recorded for the control experiment (*p* = 0.030) and mASW.N^−^ (*p* = 0.011) after 450 h of culture. Specifically, x was 299.3 ± 13.6 mg/L in the control experiment, while nitrogen limitation negatively affected the synthesis of biomass, as 195.7 ± 14.4 mg/L was produced by the end of the culture (Table 4a).

In the experiments carried out for *M. gaditana* VON5.3, cell growth was mostly favored under the control conditions (Figure 2d, Table 5a). However, despite the small differences in cell number at the end of the cultures, the highest biomass production, i.e., 523.0 ± 136.0 mg/L, which was obtained from the control culture, was higher, but not statistically significant, compared with the biomass produced at the end of the nutrient-limited cultures, reaching 393.9 ± 22.1 mg/L and 392.2 ± 38.0 mg/L in mASW.N^−^ and mASW.P^−^, respectively (Table 5a). Herein, statistically significant differences were observed only for the biomass produced between 240 h and 450 of the mASW.N^−^ (*p* = 0.012) culture and the mASW.P^−^ (*p* = 0.046) culture.

Lastly, regarding the growth curves of *N. pyriformis* PAT2.7, it was recorded that mASW.P^−^ was a condition quite favorable for cell growth, as well as mASW.N^−^ (Figure 2e). However, the tendency of the cells to form cell aggregates must be highlighted as this posed a risk for the misestimation of growth. In this context, the cell count for the culture in mASW.N^−^ ceased after the 14th day of culture (i.e., at approximately 330 h) due to the increased tendency to form cell aggregates. Regarding biomass production, the highest yield was recorded when *N. pyriformis* PAT2.7 was cultured under the control conditions (x = 471.4 ± 27.6 mg/L) and was practically at the same level in the phosphorus-limited culture (x = 452.2 ± 21.0 mg/L) (Table 6a). However, the biomass produced under nitrogen-limited conditions was at significantly lower levels at 162.0 ± 53.0 mg/L (Table 6a). Specifically, *p* was 0.019 in the set control mASW.N^−^ and in the set mASW.N^−^—mASW.P^−^, with 0.006 and 0.023 at 240 h and 450 h, respectively. Notably, statistically significant differences in the biomass production of the same culture were recorded only under control conditions (*p* = 0.016).

### 3.2. Synthesis of Storage Materials and Fatty Acid Composition of Total Lipids and Their Fractions

*P. costavermella* VAS2.5 showed an adequate to relatively high lipid reserve accumulation capacity under nutrient-limited conditions. To be more specific, the cultures performed in control conditions and under phosphorus limitation led to L/x% ≈ 16% (wt/wt) at the end of both cultures (Table 2a). A statistically significantly lower lipid content, compared with the above (*p* = 0.0004) was recorded for this strain under nitrogen-limitated conditions (L/x% = 10.4 ± 0.3%, wt/wt). The major lipid fraction in all culture conditions was that of G + S, in high percentages (i.e., 54.9–70.5%, wt/wt), while lipid fractions N and P appeared in lower and comparable proportions (Table 2a). Regarding the polysaccharide content of the biomass, percentages in the range of S/x% = 10–15.2 (wt/wt) were observed between the culture in control conditions and mASW.N^−^, which were statistically significantly different (*p* was 0.0001 and 0.0008, respectively, at 240 h and 450 h of culture). The polysaccharide content of the culture in mASW.P^−^, which at the end of the culture was S/x% = 1.5 ± 0.2%, wt/wt, was, as expected, significantly lower than the other culture conditions at both time checkpoints as well. Also, the protein content was high, especially in the control medium where it exceeded 50%, wt/wt, while in the other culture conditions the percentage ranged between 24.5 and 35.6%, wt/wt (Table 2a). Statistically significant differences were recorded in all cases herein, independently of the time of incubation or culture condition. Finally, total chlorophyll and carotenoids values were lower in mASW.N^−^ and mASW.P^−^ compared with the control medium (Table 2a).

The composition of total lipids and their lipid fractions in fatty acids of *P. costavermella* VAS2.5 when cultured in control and nutrient-limited conditions is shown in Table 2b. The main PUFA synthesized under all culture conditions was 20:5(n-3), ranging from 13.2 to 21.0% (wt/wt) with the most favorable condition being nitrogen limitation. Notably, the content of 20:5(n-3) was slightly higher, i.e., 23.9 ± 0.3% (wt/wt), when this strain was cultured in conditions similar to the control but at pH = 7.5 (Table S1), while it was lower, i.e., 8.0 ± 1.0% (wt/wt), when a 16:8 photoperiod was applied (Table S2). Similar percentages of 20:5(n-3) were also present in the polar lipid fractions per case (9.4–24.6%, wt/wt), while, in general, it was lower in the N lipid fraction (i.e., 3.6–9.5%, wt/wt) (Table 2b). Apart from 20:5(n-3), the other fatty acids produced in significant proportions were 16:0 (17.2–25.7%, wt/wt) and 16:1(n-7) (20–30.5%, wt/wt), with their percentages in the lipid fractions changing at similar levels to those determined in total lipids. Similar results were also obtained at cultures that were carried out at pH = 7.5 with constant illumination and at pH = 8.5 with a 16:8 photoperiod (Tables S1 and S2). It is also noted that in some cases 18:4(n-3) was detected. Regarding the composition of total lipids in fatty acids, statistically significant differences were observed for the following sets: (a) control—mASW.N^−^: 14:1(n-5), 16:0, 17:0, 18:1(n-9), 18:3(n-3), and 18:4(n-3) at 240 h and 17:0, 18:0, 18:3(n-3), and 18:4(n-3) at 450h; (b) control—mASW.P^−^: 16:0 and 18:0 at 240 h and C17:0, 18:3(n-3), and 18:4(n-3) at 450 h; (c) mASW.N^−^—mASW.P^−^: 14:1(n-5), 17:0, and 18:3(n-3) at 240 h and 18:0, 18:3(n-3), and 18:4(n-3) at 450 h.

*P. oklahomense* PAT3.2B presented a lower lipid content (L/x% = 5.1–6.0%, wt/wt) regardless of the growth conditions at the end of culture compared with the control experiment (L/x% = 11.5 ± 0.3%, wt/wt). At the end of culture, no statistically significant differences were observed in terms of lipid accumulation. As mentioned before, the produced lipids were predominated by G + S (54.4 ± 3.9%, wt/wt), followed by N (34.9 ± 2.3%, wt/wt) and P (12.9 ± 1.6%, wt/wt) (Table 3a). Polysaccharide content ranged between 8.8 and 11.5% (wt/wt). Notably, statistically significant differences were observed only at 240 h of culture, in the sets control—mASW.N^−^ (*p* = 0.026) and mASW.N^−^—mASW.P^−^ (*p* = 0.027). On the other hand, protein synthesis was particularly high under control conditions and phosphorus limitation (P/x% = 49.2 ± 4.8%, wt/wt and P/x% = 45.9 ± 6.4%, wt/wt, respectively). Regarding protein content, the only significant difference in terms of statistical analysis was observed at 240 h of the set control—mASW.N^−^ (*p* = 0.002). Lastly, a high concentration of total chlorophyll and carotenoids was recorded in all the conditions tested, especially under phosphorus-limited conditions (Table 3a).

In contrast to *P. costavermella* VAS2.5, the fatty acid composition of the total lipids and their fractions, where it was possible to be analyzed, of *P. oklahomense* PAT3.2B revealed 16:0, 18:1(n-9), 18:2(n-6), and the PUFA 18:3(n-3) (13.6–23.0%, wt/wt) as the predominant fatty acids, with similar percentages in both the G and P fractions but lower in N when the microalgae were grown under control conditions (Table 3b). Similar percentages were recorded for 18:3(n-3) synthesis when this strain was cultured in mASW at pH = 7.5 with constant illumination or in mASW at pH = 8.5 with a 16:8 photoperiod (Tables S3 and S4). Other fatty acids, at significantly lower percentages, synthesized by this strain under the examined culture conditions were 14:1(n-5), 18:0, 16:1(n-7), and 14:0. Finally, an unusual finding was the presence of 17:0 at 7.2–10.2%, wt/wt, under nitrogen- and phosphorus-limiting conditions (Table 3b), as well as at 240 h of the culture that was performed at pH = 7.5 (Table S3). Regarding the composition of total lipids in fatty acids, statistically significant differences were observed for the following sets: (a) control—mASW.N^−^: 14:1(n-5) at 240 h and 18:3(n-3) at 450 h; (b) control—mASW.P^−^: 14:0 at 240 h and 14:1(n-5), 16:0, 17:0, 18:0, and 18:4(n-3) at 450 h; (c) mASW.N^−^—mASW.P^−^: 16:0, 18:0, 18:2(n-6), and 18:3(n-3) at 450 h.

Similarly to the other *P. oklahomense* strain, SAG4.4 lipid synthesis was not affected positively by nutrient limitation. In fact, L/x% ranged from 1.2 to 5.7%, wt/wt, which was lower compared with the value recorded for the strain’s culture under control conditions (L/x% = 9.4 ± 1.5%, wt/wt) (Table 4a). Notably, lipid content was statistically significantly different in the set control—mASW.N^−^ at 450 h of culture (*p* = 0.040). The major lipid fraction in the control experiment and mASW.P^−^, when it was possible to determine the lipid fractions, was that of G + S, which obtained values ranging between 67.4 and 68.7% (wt/wt), followed by N and P (Table 4a). The biosynthesis of polysaccharides was similar between the different conditions, with their concentration ranging between 10.6 and 13.1%, wt/wt. The above was confirmed by the fact that no statistically significant differences were observed for the three culture conditions. The produced biomass was rich in proteins after culture at control conditions and in mASW.P^−^ (P/x% = 38.2–43.8%, wt/wt). Yet, clearly lower protein levels were determined in mASW.N^−^ (P/x% = 14.2 ± 4.0%, wt/wt), which were statistically significantly different to the other two cultures at both 240 h and 450 of culture (Table 4a). Finally, regarding the values of pigments, these ranged between 2.6 and 4.7%, wt/wt, for total chlorophyll and between 0.5 and 1.3%, wt/wt, for carotenoids (Table 4a).

In general, the fatty acid composition of total lipids and their lipid fractions, where possible, of the other *P. oklahomense* strain, SAG4.4, was similar to the one described for *P. oklahomense* PAT3.2B (Table 4b). Specifically, the major PUFA synthesized under all culture conditions was 18:3(n-3), the concentration of which ranged from 13.5 to 23.0% (wt/wt), with the most favorable condition for its synthesis in the control experiment. Similar to the above were the percentages of this fatty acid in the individual lipid fractions. Other fatty acids produced in significant concentrations were 18:1(n-9) (14.7–17.0%, wt/wt) and 16:0 (13.4–17.1%, wt/wt), with their percentages in the various lipid fractions being generally similar to those of total lipids. The detection of the fatty acids 18:3(n-6) and 18:4(n-3) in some cases is noted as well. Similar profiles regarding the fatty acid composition of total lipids of this strain were recorded for this strain when cultured under control conditions but at pH = 7.5 instead of pH = 8.5 or with a 16:8 photoperiod instead of 24:0 (Tables S5 and S6). Regarding the composition of total lipids in fatty acids, statistically significant differences were observed for the following sets: (a) control—mASW.N^−^: 18:1(n-9) at 240 h and 17:0, 18:0, and 18:3(n-3) at 450 h; (b) control—mASW.P^−^: 17:0 and 18:2(n-6) at 240 h and 18:0 at 450 h; (c) mASW.N^−^—mASW.P^−^: 18:2(n-6) at 240 h and 17:0, 18:1(n-9), and 18:3(n-3) at 450 h.

With regard to the ability of *M. gaditana* VON5.3 to accumulate reserve materials under the examined culture conditions, the highest lipid content was determined in cells when growing in mASW.P^−^, exceeding L/x% = 19% (wt/wt), followed by the cultures under nitrogen limitation and control conditions. The main lipid fraction in all the culture conditions tested, like *Picochlora*, was that of G + S, ranging between 55.2 and 56.9% (wt/wt), with the exception of the cultures in mASW.N^−^ in which the percentage of G + S reached 71.5 ± 4.0%, wt/wt (Table 5a). The following lipid fraction was that of N and then of P lipids (Table 5a). Regardless of the culture condition, polysaccharide content remained practically unchanged and at the level of 10% (wt/wt) in this strain, while protein accumulation seemed to be favored under phosphorus-limiting conditions, in which the concentration reached 35.1%, wt/wt, compared with 16.2–21.9%, wt/wt, in the other culture conditions (Table 5a). In this set of experiments, no statistically significant differences were recorded regarding the accumulation of reserve materials, regardless of the culture condition and the time of incubation. Finally, the pigment content was significantly higher when this strain grew in mASW.N^−^ (Table 5a).

In the case of *M. gaditana* VON5.3, the major PUFA synthesized, as traced in the fatty acid composition of total lipids and lipid fractions (where possible), under all culture conditions was 20:5(n-3), ranging from 20.0 to 22.9% (wt/wt), with the most favorable condition being the culture in mASW.P^−^ (Table 5b). A slightly higher content of 20:5(n-3), i.e., 24.4 ± 1.5% (wt/wt), was exhibited by this strain when cultured under similar conditions to the control experiment but at pH = 7.5 (Table S7). In general, the percentages of 20:5(n-3) in the individual lipid fractions were in the same range as those in the total lipids in polar lipid fractions, with minor variations. On the contrary, for example, the concentration of this fatty acid was significantly lower in the N fraction, regardless of the culture conditions, ranging between 7.9 and 9.2%, wt/wt. Other fatty acids produced in significant levels were 16:0 (19.9–23.0%, wt/wt) and 16:1(n-7) (26.9–31.3%, wt/wt) acids, with the percentages in their lipid fractions ranging at similar levels to those determined in total lipids. The production of these fatty acids was comparable when *M. gaditana* VO5.3 was cultured in mASW at pH = 7.5 and a 24:0 photoperiod or at pH = 8.5 with a 16:8 photoperiod (Tables S7 and S8). It can also be noted that 18:4(n-3) was detected in all cultures except for the cultures performed under nitrogen limitation (mASW.N^−^) (Table 5b, Tables S7 and S8). Regarding the composition of total lipids in fatty acids, statistically significant differences were observed for the following sets: (a) control—mASW.N^−^: 17:0 at 240 h and 16:1(n-7) at 450 h; (b) control—mASW.P^−^: 17:0 and 18:4(n-3) at 240 h and 18:4(n-3) at 450 h; (c) mASW.N^−^—mASW.P^−^: 18:4(n-3) at both 240 h and 450 h.

The accumulation of lipid reserves in the case of *N. pyriformis* PAT2.7 was even lower, regardless of the culture conditions, with L/x% ranging from 1.7 to 6.2%, wt/wt. Notably, statistically significant differences were observed among the three examined conditions at 240 h. As previously mentioned, the predominant lipid fraction was G + S in the culture performed under control conditions, followed by N and P lipid fractions (Table 6a). Regarding the polysaccharide content of the biomass, a high accumulation rate was observed under nitrogen- (S/x% = 31.6 ± 2.4%, wt/wt) and phosphorus- (S/x% = 21.8 ± 0.7%, wt/wt) limitated conditions at the end of the cultures (Table 6a). The statistical analysis revealed that the polysaccharide content was significantly higher at 240 h of culture in mASW.P^−^, i.e., *p* was 0.040 and 0.048 for the sets control—mASW.P^−^ and mASW.N^−^—mASW.P^−^, respectively. Similarly, at 450 h, the statistical analysis confirmed what was recorded for mASW.N^−^. Moreover, the protein content was significant in all the culture conditions tested (P/x% = 22.0—37.8%, wt/wt) and especially under control conditions (P/x% = 37.8% ± 3.8%, wt/wt) (Table 6a). The protein content was statistically significantly lower compared with the other two culture conditions, i.e., *p* was 0.049 and 0.022, respectively, for the sets control—mASW.P^−^ and mASW.N^−^—mASW.P^−^. Finally, regarding the values of pigments, there was a variation of 0.8–2.3%, wt/wt for total chlorophyll, while carotenoids were detected at lower concentrations, up to 0.5%, wt/wt (Table 6a).

Lastly, the composition of total lipids and lipid fractions (where possible) in fatty acids of N. pyriformis PAT2.7 when cultured under control and nutrient-limited conditions is shown in Table 6b. The predominant fatty acids were 16:1(n-7) (39–42.1%, wt/wt) and 14:0 (23.3–31%, wt/wt). It is noted that the synthesis of both fatty acids was comparable to the above when this strain was cultured in mASW at pH = 7.5 with constant illumination but lower when cultured in mASW at pH = 8.5 and a 16:8 photoperiod (Tables S9 and S10). The respective contents of the N and G lipid fractions with respect to these fatty acids were similar to those of TLs. However, for the P lipid fraction, a generally reduced presence of both of the above fatty acids was recorded, compared with TLs, and the two aforementioned lipid fractions. Regarding the composition of total lipids in fatty acids, statistically significant differences were observed for the following sets: (a) control—mASW.N^−^: 14:1(n-5) and 16:0 at 240 h; (b) control—mASW.P^−^: 14:1(n-5) at 240 h; (c) mASW.N^−^—mASW.P^−^: 14:0 and 18:1(n-9) at 240 h.

## 4. Discussion

Microalgae are used in a variety of biotechnological applications, while, at the same time, they are an important biological material for basic research. All strains used in the current study were previously characterized biochemically, exhibiting profiles of interest, and cultured under laboratory conditions [8,13,20]. In the following paragraphs, a comparison and discussion of the results of this study with previous studies will occur, in order to expand the discussion regarding the optimum culture conditions for these strains, as well as giving prominence to the strains’ plasticity of growing in different illumination intensities, temperatures, aeration, agitation, etc.

In general, and taking into account the relevant data in the literature, the strains grew adequately, and, intriguingly, in some cases outperformed other strains of the same species or even other strains of microalgae that are commercially exploited. The predominant reserve material of all microalgae was proteins, followed by polysaccharides and lipids. In some cases, lipid biosynthesis was favored under phosphorus-limiting conditions, while nitrogen limitation seemed to inhibit the biosynthesis of biomass and reserve materials. In the same context, cultures in mASW under constant illumination at pH = 7.5 led to enhanced lipid synthesis in some cases, whereas the application of a 16:8 photoperiod when the strains were cultured in mASW at pH = 8.5 negatively affected the biosynthesis of biomass and reserve materials (see Supplementary Material). In the following paragraphs, a detailed discussion about the findings of this research and a comparison with the literature occurs.

### 4.1. Cell Growth and Biomass Production

Initially, regarding the three *Picochlorum* strains, in addition to the growth that occurred under both nutrient-limitated conditions, all strains grew despite the culture conditions. However, in most of the cases, there was less biomass compared with their cultures under control conditions in the OPSR, Erlenmeyer flasks, or Stirred Tank Reactor (STR), with cultures that lasted approximately 450 h and 250 h, as presented elsewhere [8,20]. The above seems to confirm that for these strains the light supply played a crucial role in growth. In the cultures that were carried out in the STR and Erlenmeyer flasks it was possible to provide high light intensity (i.e., 1071 μE/m^2^·s and 387 μE/m^2^·s, respectively). On the other hand, de la Vega et al. (2011) [7] cultured *Picochlorum* sp. HM1 at luminous intensities of 100 and 1200 μE/m^2^·s and observed a slight elevation in specific growth rate (from 0.031 1/h to 0.034 1/h), suggesting that the growth of this strain approaches saturation at relatively low light intensities, while no evidence for photoinhibition was observed at elevated light intensities. However, the light energy supply of the cells can be severely affected by so-called “mutual shading”, a condition where denser populations block light from penetrating deeper into the microalgal culture as cell concentration increases [41,42,43]. In the OPSR, light energy was not only of relatively low light intensity, but also introduced from the top of the bioreactor. Consequently, as cell density grew, light penetration diminished at the lower layers, creating suboptimal illumination that adversely impacted the metabolism of cells to an extent.

It is noteworthy that the culture of *P. oklahomense* SAG4.4 in mASW.P^−^ led to almost double biomass production compared with the control conditions, indicating the intra-species variability regarding the response under similar environmental conditions, as documented for these strains in Dritsas et al. (2023) [20] as well. The importance of phosphorus for the growth of *Picochlorum* has also been studied by other researchers. For example, in another study, cultures of a *Picochlorum* sp. in 0.5 M MD4 media supplemented with varying concentrations of phosphorus in the form of KH_2_PO_4_, like this study, were carried out [44]. According to the findings of that study, KH_2_PO_4_ 0.16 g/L displayed the highest cell density from day 18 to 24, a concentration which was more than double compared with the 0.07 g/L or 0.02 g/L of mASW.P^−^ in the cultures performed herein. In another study, biomass productivity decreased by nearly 30% and 57% following *Picochlorum* sp. cultures in a medium supplemented with half or none NaH_2_PO_4_. 2H_2_O compared with the control experiment [39].

On the other hand, nitrogen is a nutrient of high importance for the growth of microalgae; thus its availability is considered as one of the primary factors affecting yields. Moreover, nitrogen is one of the nutrients that is most rapidly depleted during culture under laboratory conditions. Therefore, the low yields observed for the examined *Picochlora*, especially for the two *P. oklahomense* strains, came as no surprise when cultured under nitrogen limitation (mASW.N^−^). Similar findings were reported by El-Kassas (2013) [39] after growing a *Picochlorum* strain under nitrogen limitation/starvation conditions, who observed a negative correlation between biomass production and the low concentration of nitrogen, administered in the form of NaNO_3_. The above was in agreement with the conclusion reached by Vo et al. (2023) [45] for another strain of the genus. However, it cannot be neglected that high nitrogen concentrations seem to negatively affect growth as well [39]. According to a recent study, L-Asparagine posed as a more suitable nitrogen source for improved yields in *Picochlora* [46].

*M. gaditana* VON5.3 presented slightly lower growth rates when grown in mASW.N^−^ and mASW.P^−^ compared with the culture under control conditions. Yet, this strain produced more biomass compared with the two nutrient-limited culture conditions, in which biomass production was at similar levels, denoting the sufficiency of the initial concentration of both nutrients. Interestingly, all *M. gaditana* VON5.3 cultures in the OPSR led to the production of more biomass than *Microchloropsis salina* (formerly known as *Nannochloropsis salina*) (x_max_ ≈ 0.3 g/L) which was grown in mASW for 16 days under different light intensities [47]. Additionally, it is worth mentioning that *M. gaditana* VON5.3, growing under control conditions, produced almost twice the biomass compared with the *M. gaditana* strain used by Dourou et al. (2018) [30] under similar culture conditions. The abovementioned becomes even more interesting when considering that their culture lasted more than 100 hours longer compared with the one presented herein. Nonetheless, Dourou et al. (2018) [30] confirmed the importance of a high-concentration phosphorus supply in the growth environment of *M. gaditana*, as their study recorded a positive correlation between the provided phosphorus and biomass production. On the same note, recently, Kim et al. (2024) [47], culturing *Μ. gaditana* CCMP 526 (presented as *N. gaditana* CCMP 526) under N- and P-limitated conditions, recorded lower cell numbers by 36% and 11%, respectively, compared with the control conditions. Similar findings were presented by Cecchin et al. (2020) [34] under N-limited conditions. However, the supplementation of the culture medium with urea seems to be beneficial for microalgal growth [48].

The cultures of *M. gaditana* VON5.3 in the OPSR, regardless of the culture conditions, after 240 h of culture were significantly lower compared with the biomass production that was recorded when this strain grew in STR at 25 °C, [8], as well as compared with the cultures carried out in Erlenmeyer flasks [20]. However, biomass production was comparable with what was recorded in the other studies only in the case of the culture under control conditions after 450 h of incubation [8,20], showcasing the dependence of microalgae on light intensity. In addition, a study on *M. salina* showed a positive correlation of the increase in biomass production with the intensity of the provided light energy [49]. On the other hand, another *M. salina* grown under similar culture conditions in OPSR, but at 120 μE/m^2^·s, produced a biomass of equivalent amount to that of *M. gaditana* VON5.3 [31]. In our study, the cross-shading of the cells from the 10th day of culture might hinder the growth of the culture. However, it should also be taken into consideration that according to Simionato et al. (2011) [50], significant alterations in biomass production are evident only under extreme light intensities, whereas incubation within a defined light energy threshold causes little changes in biomass production.

Lastly, biomass production by *N. pyriformis* PAT2.7 was comparable under control conditions and in mASW.P^−^, whereas culture in mASW.N^−^ resulted in nearly a threefold reduction compared with the other two set-ups. Similar findings on nitrogen limitation are also reported by Mastropetros et al. (2023) [21]. Notably, cell counting in mASW.N^−^ was terminated prematurely, as from 330 h of culture a strong tendency to form aggregates of cells was observed, indicative of the stress induced.

Similarly to the above, the growth of this strain in STR at 25 °C [8] and in Erlenmeyer flasks [20] led to higher biomass production compared with what was recorded herein. This observation suggests once again the dependence of microalgae on light intensity. However, similarly to what was previously discussed, the cross-shading from the 10th day of culture played an important role since the cultures presented a dark green color, indicating the insufficient availability of the supplied light in the lower layers of the culture. Moreover, it is of interest to mention the apparent plasticity in the response to different temperature values, with the optimal growth temperature varying between species or even strains of *Nephroselmis* species. For instance, the ability of *Nephroselmis* to grow at 20–21.5 °C, marking relatively high biomass production, has been reported [8,9], while other representatives of the genus grow efficiently at higher temperatures (e.g., 25–27 °C) [19,23,51]. In any case, it is worth mentioning that the enrichment of the culture medium with vitamin B12 has been shown to positively affect the production of biomass by *Nephroselmis astigmatica* [52].

### 4.2. Synthesis of Storage Materials

The cultures of *P. costavermella* VAS2.5 under control conditions and phosphorus limitation favored lipid synthesis. The same was the case for the two *P. oklahomense* strains as well. Phosphorus limitation, as well as nitrogen limitation, though to a lesser extent, proved to be a particularly favorable condition for lipid accumulation in other *Picochlorum* strains as well [39,53], while Nguyen et al. (2024) [44] reported higher lipid accumulation when KH_2_PO_4_ = 0.16 g/L. In contrast, lipid accumulation in all *Picochlora* cultures used herein was negatively affected by nitrogen limitation. Undoubtedly, the above can be directly correlated with the reduced biomass production of the strains under these conditions. However, when Anto et al. (2024) [46] cultured *Picochlorum* sp. NITT 04 under various combinations of N and P, lipid content maximization under a N:P ratio of 1.4:0.6 and salinity of 24 ppt (i.e., L/x% ≈ 50%, wt/wt) was observed.

Similar levels of polysaccharide content were recorded under the different culture conditions for the three strains, except for the culture of *P. costavermella* VAS2.5 in mASW.P^−^, where intracellular polysaccharides were almost fully consumed by the end of the culture. Such physiological characteristics are strongly related to the need of the cells for energy and metabolic precursors at the expense of the reserve materials when growing under environmental stressors. In addition, in some cultures, a decrease in lipid stock was noted which was accompanied by an increase in polysaccharide concentration, and vice versa, indicating that the two biosynthetic pathways are competitive with each other, or even that the biotransformation of sugars to lipids and vice versa occurred. According to Bellou & Aggelis (2012) [31], these physiological characteristics can be attributed to the need of the cells for energy and metabolic precursors at the expense of the reserve materials when growing under environmental stressors.

High protein accumulation was also observed for all strains herein, in the range of 35–50% (wt/wt). This corroborates earlier predictions, since the ability of *Picochlora* to accumulate proteins at high levels has been well documented [8,11,13,14,20,39,53,54]. However, the protein content of the examined *Picochlorum* strains cultured in mASW.N^−^ was significantly lower compared with the other cultures, ranging from 14 to 25% (wt/wt). These findings are in accordance with what El-Kassas (2013) [39] described for other *Picochlorum*. Specifically, under nitrogen starvation conditions, other *Picochlorum* strains had shown dramatic reductions and low rates of intracellular protein accumulation when growing on various substrates and under various conditions [39]. In addition, El-Kassas et al. (2013) [39] recorded a significant decrease in protein content under phosphorus-limitated conditions, which in this case seems to be verified only in the case of *P. costavermella* VAS2.5, in which a decrease from 74.1 ± 4.1% at 240 h to 35.6 ± 0.4%, wt/wt at 450 h of culture was recorded. In any case, the fact that members of *Picochlorum* possess amino acid profiles of high commercial and biotechnological interest is of particular value. For instance, according to Dritsas et al. (2025) [13], *P. costavermella* VAS2.5 and *P. oklahomense* SAG4.4 proved to be great producers of lysine and threonine.

Finally, with regard to the pigments produced, i.e., chlorophylls and carotenoids, which usually are rich in lutein and zeaxanthin in *Picochlora* [7,14,55], generally similar or lower production was observed compared with other studies. Notably, in all measurements, chlorophyll-a was produced in significantly greater amounts than chlorophyll-b. This pattern aligns with the findings from previous studies on other strains within the genus [14,53]. However, nutrient limitation proved to be less suitable for the greater synthesis of pigments by *Picochlorum* strains in comparison with the control culture conditions. Nonetheless, the pigment content of the cells is influenced by many parameters, such as the growth phase of the culture, the intensity of the light energy supplied, and the CO_2_ concentration [56]. In certain microalgae, e.g., *H. pluvialis*, *Dunaliella salina*, providing a high light intensity or gradually increasing the temperature in the culture environment, parameters that stress cells and activate the photoprotection mechanisms of microalgae, is considered to be a safe and effective strategy to increase carotenoid synthesis [57]. The aforementioned seem to apply in *Picochlorum* as well, some species of which can grow sufficiently under light intensities of up to 2000 μE/m^2^·s and temperatures up to 39 °C [6,8,58]. Another influential parameter is nutrient availability. However, the phosphorus concentration in the culture medium did not seem to affect high pigment content in the study of Nguyen et al. (2024) [44]. On the other hand, LaPanse et al. (2024) [54] highlighted the trend of *Picochlorum celeri* reducing pigment content under nitrogen limitation, as the cells direct cellular metabolism towards polysaccharide production and away from nitrogen-rich macromolecules. However, another important parameter that can affect chlorophyll production is the concentration of Fe in the microalgal growth medium [59]. In particular, experiments conducted on a *P. oklahomense* (presented as *P. oklahomensis)* strain presented that iron limitation significantly reduced total chlorophyll per cell, up to 41% when the initial Fe concentration was half that of the control experiment [59], and a decrease that correlated with Fe concentration was also shown for the carotenoids produced.

In contrast to what was observed for the *P. oklahomense* strains used in this study, the most favorable condition for lipid accumulation in *M. gaditana* VON5.3 proved to be phosphorus limitation, since it approached 20% (wt/wt) at the end of culture. Interestingly, the lipid content was slightly higher than that of the commercial strain of the ‘sibling’ genus *Nannochloropsis*, such as *Nannochloropsis* sp. from Reed Mariculture (Campbell, CA), which exceeded 17% (wt/wt), but was lower compared with what was recorded for *Nannochloropsis oceanica* F&M-M24, which approached 30% (wt/wt) [60,61]. Similar results to this study were obtained by Dourou et al. (2018) [30] who recorded 2–3 times higher lipid accumulation levels when another strain of the species was grown under phosphorus-limiting conditions, exceeding up to 30% (wt/wt). On the other hand, nitrogen limitation did not seem to favor lipid accumulation, which was at similar levels to the control experiment. In agreement with these findings are the results of another study on *M. salina*, which reported that lipid production was not induced under nitrogen-limitated conditions [62], whereas the opposite conclusions were reached by other studies [63,64,65]. Therefore, the determination of the proper concentration of the nitrogen source is of most importance for the optimization of lipid yields.

The polysaccharide content of *M. gaditana* VON5.3 under nutrient limitation was slightly increased compared with the control medium. On the other hand, the results in this case were comparable to what was recorded for the cultures of this strain under the other balanced growth conditions of cultures in Erlenmeyer flasks [20] and the STR [8]. Moreover, despite the comparable amounts of polysaccharides produced by the *M. salina* strain that Bellou & Aggelis (2012) [31] used, it must be pointed out that *Microchloropsis* strains are able to produce polysaccharides in the range of 20–25% (wt/wt) of their dry biomass [30].

Among the cultures of *M. gaditana* VON5.3 in the OPSR under nutrient-limited conditions, mASW.P^−^ stood out in terms of protein synthesis, at approximately 35% (wt/wt). Notably, this protein content was significantly higher than what was recorded for the cultures of this strain in Erlenmeyer flasks and the STR [8,20]. Notably, the protein content after culture in mASW.P^−^ was comparable to that of other *Microchloropsis* strains [11,30,31]. On the contrary, it came as no surprise that the lowest protein content was recorded under nitrogen-limitated conditions.

Finally, *M. gaditana* VON5.3 showed a relatively high pigment content, which was at similar levels for all tested culture conditions and in most of the cases comparable to the cultures of this strain in other types of photobioreactors [8,13,20]. This feature offered an increased capacity in light energy uptake and contributed positively to their adaptation under the given culture conditions. The sole exception was the culture in mASW.P^−^, in which the chlorophyll composition was very low. In another study, nitrogen limitation decreased photosynthetic activity and chlorophyll content, while phosphorus limitation led to an increase in carotenoid content [47]. Overall, since *Microchloropsis* are known producers of carotenoids containing high levels of violaxanthin and β-carotene [47,66], it is important to optimize the conditions that will enhance their production levels.

Lastly, the lipid production of *N. pyriformis* PAT2.7 was low, regardless of the culture conditions. Neither nitrogen nor phosphorus limitation led to enhanced lipid synthesis rates for *N. pyriformis* PAT2.7. Although the available literature on the ability of strains of this genus to accumulate lipids is quite limited, the results of the present study are comparable to those of another *Nephroselmis* strain (i.e., L/x% = 10.5%, wt/wt), which was grown for about one week in a nutrient medium at 25 °C, with a salinity of 28‰ and a light intensity of 80–100 μE/m^2^·s [67]. *Nephroselmis* sp. (var. Messolonghi) of Hotos et al. (2023) [68] grown in Walne medium accumulated lipids at 15%, wt/wt. Even higher lipid accumulation was exhibited by *Nephroselmis* sp. KGE1 grown on Bold’s Basal Medium (i.e., which contained KNO_3_ = 0.25 g/L as the nitrogen source), with L/x% = 33.0 ± 0.06% (wt/wt) [51]. From the above, it is obvious that lipid production in *Nephroselmis* is significantly affected by the availability of nitrogen in the growth environment. Therefore, optimization of the culture medium is required. At the same time, the above suggests the different response of strains of the genus to similar types of environmental stresses. In contrast, phosphorus limitation, although not an inhibitory factor for the growth of *N. pyriformis* PAT2.7, led to the lowest yield among the cultures performed, regardless of the type of bioreactor [8,13,20].

Interestingly, *N. pyriformis* PAT2.7 seemed to be benefit from nutrient limitation in other aspects of its metabolism, as an increase in the polysaccharide content was observed under nitrogen- or phosphorus-limitated conditions, reaching S/x% = 31.6 ± 2.4% (wt/wt) and S/x% = 21.8 ± 0.7% (wt/wt), respectively. The results of the present study are in agreement with the study of Mastropetros et al. (2023) [21], who recorded polysaccharide contents of 45% (wt/wt) for *Nephroselmis* sp. grown under nitrogen limitation and high illumination. Moreover, the authors suggested that the application of high salinity and light intensity, as environmental stresses, led to increased polysaccharide production in *Nephroselmis*.

Regarding protein production, a relatively high content was observed in the *N. pyriformis* PAT2.7 culture under control conditions, which was lower in comparison with the cultures carried out in other types of bioreactors [8,13,20]. On the other hand, nutrient limitation did not enhance protein synthesis in *N. pyriformis* PAT2.7. The lower protein content of *N. pyriformis* PAT2.7 grown under nitrogen or phosphorus limitation confirmed once again that under conditions of environmental stress, microalgae channel energy towards energy conservation and/or growth. However, it is essential to acknowledge that protein production in *Nephroselmis* can be increased at higher incubation temperatures [21,67].

Lastly, pigment production was generally of the same level in *N. pyriformis* PAT2.7 cultures and at similar or below the levels reported in other studies [8,13,21,23,69,70]. The carotenoids of *Nephroselmis* are of particular interest, since they mainly consist of beta-carotene, siphonaxanthin, neoxanthin, and lutein, molecules with proven antioxidant and anti-inflammatory activity [71,72].

### 4.3. Fatty Acid Composition of Total Lipids and Their Lipid Fractions

The main lipid fraction for the three *Picochlorum* strains was the lipid fraction G + S, usually followed by the N lipid fraction and then by P. In general, the dominance of the G + S fraction over the total lipids seems to be a shared feature for a plethora of microalgae of different genera, as has been confirmed by various reports [8,13,14,73,74]. This can be attributed to the fact that G + S constitute the primary components (in percentages up to 80–90%) of chloroplasts and thylakoid membrane lipids [63,75,76,77] and phospholipids are the predominant structural lipids in microalgae. The glycolipids (G) of microalgae are considered an important source of omega-3 fatty acids, with a variety of applications, since they are known for their antimicrobial, antiviral, and anti-cancer properties [78,79]. Sphingolipids (S) represent a diverse group of membrane lipids which are characterized by highly conserved evolutionary traits and play a crucial role in cell communication, both with the environment and other cells, as well as in several key cellular processes, such as the control of cell division [80,81].

The level of accumulation of the G + S fraction in the total lipids of a microalgae is influenced by many parameters, such as the composition of the nutrient medium, the photoperiod, the intensity of the light energy provided, the growth phase of the culture, etc. [8,56,82]. For instance, *Picochlorum* sp. D3 accumulated a significantly lower percentage of glycolipids (≈ 34%) compared with the strains used herein, but overall, the polar lipids of the strain exceeded 75% of the total lipids [14], similarly to the *Picochlora* used in the present study. It is noted that glycolipids can also play the role of storage molecules and be utilized as a source of carbon and energy, both in heterotrophic (under conditions of paucity of external carbon source) and autotrophic microorganisms [83,84]. Notably, lipid fraction analyses for *P. costavermella* VAS2.5 revealed that the lipid fraction G + S in cultures grown in mASW.N^−^ and mASW.P^−^ increased from 54.9% of the control conditions to 70.5% and 68.7%, respectively. This observation has also been reported elsewhere. For example, Damiani et al. (2010) [85], growing *Haematococcus pluvialis* under nitrogen-deficient conditions, recorded an increase in glycolipid accumulation. Similar findings were obtained from the study by Wang et al. (2016) [86] on glycolipid accumulation for the microalgae *Chlorella pyrenoidosa* grown in growth medium containing nitrogen or phosphorus at various concentrations. The highest rates of glycolipid accumulation in both microorganisms occurred under nitrogen-deficient conditions. Another strain of *Chlorella* increased its glycolipid content when grown in phosphorus-limiting nutrient medium [87]. In general, a possible explanation for the increase in glycolipid content under nitrogen- and phosphorus-limited conditions is that glycolipids make up for the reduction in other lipid types in order to maintain cell homeostasis. On the other hand, nitrogen deficiency can lead to the reduction in cellular content in thylakoid membranes, the activation of hydrolytic enzymes, and the hydrolysis of phospholipids [88], while phosphorus deficiency can cause a drastic reduction in membrane phospholipids and their replacement by non-phosphorus glycolipids and sulfolipids [89].

Regarding the fatty acid composition of total lipids and the lipid fractions of the examined strains of the genus *Picochlorum*, the significant synthesis of 18:3(n-3) and 20:5(n-3) was recorded for the *P. oklahomense* strains and *P. costavermella* VAS2.5, respectively. The most favorable conditions for 18:3(n-3) synthesis proved to be the control conditions and the least favorable involved the application of a 16:8 photoperiod. The consumption of 18:3(n-3) by humans in reasonable limits can reduce triglycerides, total cholesterol, high-density lipoprotein, and low-density lipoprotein, hence, it is associated with a lower risk of cardiovascular disease and a reduced risk of fatal coronary heart disease [90,91,92]. Intriguingly, the 18:3(n-3) content of the isolated strains was higher than other strains recorded in the literature, e.g., *Picochlorum* HM1 [7], *P. oklahomense* UTEX B 2795 (presented as *P. oklahomensis* UTEX B 2795) [11,15], *Picochlorum* sp. D3 [14], *Picochlorum* Azisu1, *Picochlorum* Azisu2 [55], and *P. maculatum* [93]. In contrast, the strain used by Dahmen et al. (2014) [53] synthesized 18:3(n-3) at 26.5 ± 1.1% of total lipids. On the other hand, it is of great interest that *P. costavermella* VAS2.5 synthesized 20:5(n-3) at significant concentrations, especially in the P fraction, where PUFAs are biosynthesized. This PUFA is of particular value, since it is a biosynthetic precursor of prostaglandins-3, which inhibit platelet aggregation, thromboxane-3, and eicosanoids, including leukotriene-5, highlighting its anti-inflammatory and cardioprotective profile [94]. To our knowledge, the presence of this fatty acid has been traced in only one other strain of the genus, though at much lower levels [95]. The high content of *P. costavermella* VAS2.5 in 20:5(n-3) may explain the very low percentages or even the occasional absence of 18:3(n-3) and 18:4(n-3), according to the mechanisms of PUFA synthesis described in other studies [94,96] and thoroughly discussed in previous studies related to the strains used herein [8,13]. Additionally, *P. costavermella* VAS2.5 synthesized 16:1(n-7) in significant quantities, a fatty acid to which, among other things, anti-inflammatory properties and the ability to reprogram the intestinal microflora are attributed [97,98]. Notably, only *P. costavermella* VAS2.5 demonstrated the elevated synthesis of 20:5(n-3) and 16:1(n-7) compared with the control experiment, especially under nitrogen limitation.

In the cultures of *M. gaditana* VON5.3, when it was possible to determine the lipid fractions, the G + S fraction was found to be dominant. The predominance of G + S lipids over N in members of the genus has been documented elsewhere [8,13,30,31]. However, it should not be neglected that a crucial parameter for total lipid composition seems to be the age of the culture. For example, *M. gaditana* 1049 was shown to synthesize mainly N lipids; however, this changed after the 16th day of cultivation [99]. The authors interpret this phenomenon as a consequence of a shift in the lipid biosynthesis pathways of aged cultures, redirecting from chloroplasts and other cell membranes mainly towards the production of N lipids. With regard to the lipid composition of *M. gaditana* VON5.3, the very high percentage of G + S over total lipids is noteworthy, exceeding 70% (wt/wt) when *M. gaditana* VON5.3 was grown in mASW.N^−^, which can be explained by what was discussed above for *Picochlora*. Overall, given that G + S are richer in PUFAs, *M. gaditana* VON5.3 under specific growth conditions appears to be a rather attractive candidate for PUFA production.

*M. gaditana* VON5.3 produced 16:1(n-7) and 20:5(n-3) in significant quantities, especially in polar lipids, at similar levels compared with the control conditions, in both nitrogen- and phosphorus-limited cultures. As mentioned in the previous sub-section on balanced growth cultures, a similar pattern in total lipids and their lipid fractions was recorded for other *Microchloropsis* strains of other previously reported studies [30,31,100,101]. Furthermore, *M. gaditana* VON5.3 presented a significantly higher 20:5(n-3) content compared with the nine *Nannochloropsis* and *Microchloropsis* strains used in a study carried out by Ma et al. (2014) [102], where the most productive strain in terms of 20:5(n-3) did not exceed 13% (wt/wt) of total lipids. Moreover, in many cases, the content of *M. gaditana* VON5.3 in 20:5(n-3) was comparable to the commercial strains that were used by Castejón & Marko (2022) [100] and Abdelkarim et al. (2025) [40] in their studies. The above suggests the ability and suitability of *M. gaditana* VON5.3 as a source of lipid production of high nutritional and medicinal value.

Lastly, regarding *N. pyriformis* PAT2.7, interestingly, in contrast to what was recorded for its culture under control conditions and the other microalgae that were examined herein, the dominant lipid fraction was N when this strain was cultured in mASW at pH = 7.5 under constant illumination or in mASW at pH = 8.5 and a photoperiod of 16:8. These findings can be justified by the fact that some microalgae increase their N lipid content under environmental stress conditions, as was probably the case for *N. pyriformis* PAT2.7. Notably, that was also the case when this strain grew at 20 °C [8], a condition in which polar lipids usually dominate. In general, at low temperatures, the presence of saturated lipids in cell membranes reduces their fluidity and thus negatively affects cellular metabolism. Consequently, low temperatures are expected to stimulate an increased synthesis of unsaturated fatty acids, a feature that could increase survival under such unfavorable conditions [103,104,105]. The *notably* high content in N lipid reserves is another indication of the ability of this microalga to survive at 20 °C. However, Alboresi et al. (2016) [106] recorded a high N content in *M. gaditana*, as well, a feature that was attributed by the authors to the high light intensity provided to this microalga. On the other hand, it was not possible to further investigate the total lipid composition in their lipid fractions under nitrogen and phosphorus limitation due to the insufficient amount of extracted lipids.

Regarding the composition of lipids and their lipid fractions in fatty acids, where it was possible to determine them, the composition of mainly saturated fatty acids and the absence of PUFAs was observed. These results give prominence to this strain as a more suitable potential candidate for the biodiesel production industry. Nonetheless, it cannot be ignored that the predominant fatty acid in each case was 16:1(n-7) at outstanding levels. This fatty acid occurred in similar proportions in the N and G + S lipid fractions, while its proportion was reduced by half in the P fraction. The significant production of 16:1(n-7) by *N. pyriformis* PAT2.7 is considered to be of high importance and possibly opens up new prospects for the exploitation of this microalga.

Nonetheless, lipid biosynthesis depends on the growth phase of the microalgae. For example, the harvested biomass of *N. pyriformis* PAT2.7 during the late exponential or static phase contained increased amounts of N. Other strains of the genus synthesized 16:1(n-7) (3.9–6.1%, wt/wt) in much smaller amounts [19,67] but also amounts of linolenic acids (18:3(n-6) and 18:3(n-3)), which were not detected in the case of *N. pyriformis* PAT2.7, which in total approached 15% (wt/wt) of total lipids. However, it is worth mentioning that 18:3(n-6) in other works was detected at 37.7% and 56.4% of total lipids [51,69].

## 5. Conclusions

All strains used in this study generally produced a satisfactory amount of biomass when grown under control conditions, though higher biomass production was recorded for *P. oklahomense* SAG4.4 and *N. pyriformis* PAT2.7 under phosphorus-limited conditions. Conversely, nitrogen limitation emerged as the most unfavorable condition for microalgal growth, highlighting the essential role of adequate nitrogen availability in the growth medium. In addition, the application of a 16:8 photoperiod revealed the pronounced influence of both light intensity and exposure duration on microalgal growth. In contrast, the optimal condition for lipid accumulation for all strains was cultivation at pH 7.5, with phosphorus limitation also proving beneficial in certain cases. Also, the protein and pigment content were usually higher under the aforementioned conditions. Nonetheless, the polysaccharide content of the examined microalgae ranged at similar levels, regardless of the culture conditions. With regard to the composition of lipids and lipid fractions in fatty acids, the PUFAs 20:5(n-3), in the cases of *P. costavermella* VAS2.5 and *M. gaditana* VON5.3, and 18:3(n-3), for *P. oklahomense*, were traced in significant quantities, mostly in the dominant polar lipids, regardless of the culture condition. The exception was *N. pyriformis* PAT2.7, for which a low lipid composition and a general dominance of N lipids was recorded. At the same time, it is worth mentioning that in the polar lipids of *P. costavermella* VAS2.5 and *M. gaditana* VON5.3, 16:1(n-7) was also high, indicating their suitability as a source of lipid production of high nutritional and medicinal value.

Overall, the findings of this study have demonstrated the potential of the examined strains to be exploited under specific culture conditions for commercial purposes, primarily due to their interesting lipid profiles and high protein levels, features of obvious interest for the aquaculture sector. However, since research has shown that the growth capacity and productivity of strains vary between genera, species, and even between strains and is highly influenced by the culture conditions, further optimization is needed with respect to the desired metabolic product.

## Figures and Tables

**Table 1:** The composition of the modified Artificial Seawater (mASW) used as growth medium for control cultures and as basal growth medium for the rest of the cultures carried out in this study.

**Compound**	**Supplier**	**Concentration (g/L)**
NaCl	PENTA (Prague, Czech Republic)	27.0
MgSO_4_·7H_2_O	PanReac AppliChem (Darmstadt, Germany)	6.6
CaCl_2_	PENTA	1.5
KNO_3_	Scharlau (Barcelona, Spain)	1.0
KH_2_PO_4_	Himedia (Mumbai, India)	0.07
FeCl_3_·6H_2_O	BDH (Poole, UK)	0.014
Na_2_EDTA	Merck (Darmstadt, Germany)	0.019
**Microelement solution**		
**Compound**	**Supplier**	**Concentration (mg/L)**
ZnSO_4_·7H_2_O	Merck	40.0
H_3_BO_3_	Fluka (Steinheim, Germany)	600.0
CoCl_2_·6H_2_O	Sigma-Aldrich (St. Louis, MO, USA)	1.5
CuSO_4_·5H_2_O	BDH	40.0
MnCl_2_	Sigma-Aldrich	400.0
(NH_4_)_6_MO_7_O_24_·4H_2_O	Sigma-Aldrich	370.0

**Table 2:** (a) Biomass production, reserve materials accumulation, and growth parameters, (b) fatty acid composition of total lipids (TLs) and their lipid fractions (neutral—N, glycolipids—G, and phospholipids—P) of *Picochlorum costavermella* VAS2.5 grown in modified Artificial Seawater (mASW) and the different variations in growth in an 8.7 L (V_w_ = 5 L) capacity Open Pond Simulating Reactor (OPSR).

**a**	**Growth** **Medium**	**t** **(h)**	**Biomass** **(x)**	**Lipids** **(L)**				**Polysaccharides** **(S)**		**Proteins** **(P)**	**Pigments**		**Growth** **Parameters**			
			**x** **(mg/L)**	**L/x** **(%)**	**Lipid Fractions (%)**			**S/x** **(%)**		**P/x** **(%)**	**TCh/x** **(%)**	**TC/x** **(%)**	**μ** **(1/d)**		**R^2^**	
					**N**	**G + S**	**P**									
**Balanced growth**	**Control**	240	100.7 ± 12.4	22.9 ± 1.9	UND	UND	UND	12.1 ± 0.1		45.1 ± 6.4	0.4 ± 0.0	0.2 ± 0.0	0.19 ± 0.00		0.97	
		450	269.1 ± 6.7	16.1 ± 0.0	16.6 ± 0.3	54.9 ± 6.2	28.5 ± 6.5	15.2 ± 0.3		51.0 ± 2.5	5.1 ± 0.2	2.4 ± 0.8				
**Nutrient** **limitation**	**mASW.N** ^−^	240	51.5 ± 0.6	3.7 ± 0.1	UND	UND	UND	8.2 ± 0.0		15.3 ± 0.0	UND	UND	0.16 ± 0.04		0.99	
		450	245.6 ± 8.9	10.4 ± 0.3	12.1 ± 2.5	70.5 ± 7.7	17.4 ± 1.9	10.0 ± 0.0		24.5 ± 0.7	0.3 ± 0.0	0.3 ± 0.0				
	**mASW.P** ^−^	240	168.3 ± 28.0	5.6 ± 0.9	UND	UND	UND	7.3 ± 0.1		74.1 ± 4.1	UND	UND	0.13 ± 0.03		0.95	
		450	201.6 ± 0.4	16.1 ± 0.0	16.5 ± 0.2	68.7 ± 3.1	14.9 ± 3.3	1.5 ± 0.2		35.6 ± 0.4	0.7 ± 0.0	0.3 ± 0.0				
**b**	**Growth** **medium**	**t (h)**	**Lipid** **fraction**	**Fatty acid composition of total lipids and their fractions (%, wt/wt)**												
				**14:0**	**14:1(n-5)**	**16:0**	**16:1(n-7)**	**17:0**	**18:0**	**18:1(n-9)**	**18:2(n-6)**	**18:3(n-3)**	**18:4(n-3)**	**20:1(n-9)**	**20:5(n-3)**	* Others
**Balanced** **growth**	**Control**	240	TL	5.1 ± 1.1	3.2 ± 0.1	16.5 ± 0.8	24.0 ± 4.0	<0.1	1.5 ± 0.0	25.2 ± 5.6	4.8 ± 1.8	<0.5	ND	2.5 ± 0.6	16.4 ± 3.2	1.7 ± 0.3
		450	TL	4.0 ± 1.4	3.3 ± 1.0	17.2 ± 2.4	20.0 ± 4.6	<0.1	1.5 ± 0.4	30.8 ± 11.5	6.5 ± 2.6	<0.5	ND	1.9 ± 0.3	13.2 ± 4.4	1.5 ± 0.5
			N	3.8 ± 1.5	3.8 ± 1.7	25.0 ± 1.5	26.4 ± 6.5	<0.5	1.9 ± 0.7	26.3 ± 12.6	2.6 ± 1.5	0.7 ± 0.0	ND	3.5 ± 2.7	3.6 ± 1.1	2.1 ± 0.9
			G	9.0 ± 0.5	4.7 ± 1.8	19.1 ± 2.3	29.1 ± 2.3	<0.5	1.1 ± 0.8	6.6 ± 0.1	1.8 ± 0.3	<0.5	ND	1.9 ± 0.1	21.8 ± 3.8	2.4 ± 0.6
			P	1.3 ± 0.3	1.1 ± 0.6	16.4 ± 1.4	18.0 ± 2.9	<0.5	1.3 ± 0.2	39.4 ± 10.1	7.0 ± 0.6	0.7 ± 0.4	ND	4.5 ± 2.9	9.4 ± 2.6	1.0 ± 0.1
**Nutrient limitation**	**mASW.N^−^**	240	TL	4.9 ± 0.4	<0.5	22.0 ± 0.4	24.6 ± 0.5	1.0 ± 0.1	<0.1	5.3 ± 0.5	1.6 ± 0.4	12.7 ± 1.0	3.0 ± 0.3	3.9 ± 0.6	13.9 ± 0.5	9.9 ± 1.0
		450	TL	7.7 ± 0.6	4.7 ± 0.6	22.4 ± 1.5	30.5 ± 1.0	0.6 ± 0.1	6.5 ± 0.3	4.9 ± 1.1	1.1 ± 0.3	ND	ND	3.1 ± 0.8	21.0 ± 0.9	8.3 ± 0.2
			N	6.4 ± 1.3	16.0 ± 2.8	23.4 ± 1.0	28.4 ± 2.2	0.5 ± 0.1	1.5 ± 0.0	6.6 ± 0.9	1.2 ± 0.1	ND	ND	1.5 ± 0.4	6.4 ± 0.4	7.0 ± 0.9
			G	9.5 ± 0.9	2.7 ± 1.0	21.6 ± 0.4	30.7 ± 0.1	0.6 ± 0.0	1.4 ± 0.1	3.8 ± 0.3	1.1 ± 0.0	ND	ND	3.1 ± 0.0	24.6 ± 1.8	1.5 ± 0.5
			P	3.0 ± 0.3	0.5 ± 0.0	21.4 ± 0.5	28.5 ± 1.0	0.9 ± 0.2	<0.5	11.8 ± 1.7	3.9 ± 0.7	<0.5	ND	6.2 ± 0.8	20.8 ± 0.1	2.5 ± 0.3
	**mASW.P^−^**	240	TL	7.2 ± 0.0	2.8 ± 0.1	24.9 ± 0.4	28.5 ± 1.6	<0.5	0.6 ± 0.2	6.6 ± 0.5	1.7 ± 0.6	0.5 ± 0.3	2.3 ± 0.0	4.6 ± 2.0	19.6 ± 2.6	1.5 ± 0.2
		450	TL	7.0 ± 0.2	2.6 ± 0.2	25.7 ± 0.8	26.9 ± 1.6	<0.5	1.5 ± 0.7	8.4 ± 1.3	1.4 ± 0.2	ND	<0.5	3.9 ± 0.0	17.4 ± 3.0	5.1 ± 3.4
			N	4.1 ± 0.6	21.5 ± 0.2	17.2 ± 0.4	23.2 ± 2.0	ND	0.7 ± 0.0	5.8 ± 0.6	<0.5	ND	2.7 ± 0.0	3.0 ± 0.3	9.5 ± 1.6	10.8 ± 1.4
			G	8.3 ± 1.0	3.1 ± 1.2	24.6 ± 0.7	32.0 ± 0.8	ND	<0.5	4.4 ± 1.6	1.4 ± 0.4	ND	<0.5	2.9 ± 0.1	19.3 ± 3.0	3.3 ± 0.8
			P	5.0 ± 2.3	2.6 ± 1.7	25.6 ± 0.3	30.4 ± 0.8	ND	<0.5	9.1 ± 3.0	2.6 ± 0.8	0.9 ± 0.1	ND	6.9 ± 1.0	16.3 ± 0.0	1.0 ± 0.1

Abbreviations: (a) x (mg/L), dry biomass; L/x (%), lipids on dry biomass; N (%), neutral lipid fraction of total lipids; G + S (%), glycolipid and sphingolipid fraction of total lipids; P (%), fraction of phospholipids on total lipids; S/x (%), intracellular polysaccharides on dry biomass; P/x (%), intracellular proteins on dry biomass; TCh/x (%), total chlorophyll (chlorophyll a and b) on dry biomass; TC/x (%), total carotenoids on dry biomass; μ (1/d), maximum specific growth rate; R^2^, R-squared statistical measure; UND, undetermined. (b) ND: not detected. * Others: mainly 10:0, 12:0, and in some cases 18:3(n-6) Note: only glycolipids (i.e., G fraction) are mentioned, as the amide bond of sphingolipids resists methanolysis during methyl esterification.

**Table 3:** (a) Biomass production, reserve materials accumulation, and growth parameters, (b) fatty acid composition of total lipids (TLs) and their lipid fractions (neutral—N, glycolipids—G, and phospholipids—P) of *Picochlorum oklahomense* PAT3.2B grown in modified Artificial Seawater (mASW) and the different variations in growth in an 8.7 L (V_w_ = 5 L) capacity Open Pond Simulating Reactor (OPSR).

**a**	**Growth** **Medium**	**t** **(h)**	**Biomass** **(x)**	**Lipids** **(L)**				**Polysaccharides** **(S)**		**Proteins** **(P)**	**Pigments**		**Growth** **Parameters**	
			**x** **(mg/L)**	**L/x** **(%)**	**Lipid Fractions (%)**			**S/x** **(%)**		**P/x** **(%)**	**TCh/x** **(%)**	**TC/x** **(%)**	**μ** **(1/d)**	**R^2^**
					**N**	**G + S**	**P**							
**Balanced growth**	**Control**	240	100.0 ± 9.1	13.9 ± 1.5	UND	UND	UND	13.1 ± 0.6		58.4 ± 0.9	25.2 ± 1.7	4.7 ± 0.5	0.23 ± 0.03	0.96
		450	421.1 ± 30.8	11.5 ± 0.3	34.9 ± 2.3	54.4 ± 3.9	12.9 ± 1.6	8.8 ± 0.6		49.2 ± 4.8	9.0 ± 2.8	1.5 ± 0.6		
**Nutrient** **limitation**	**mASW.N** ^−^	240	49.3 ± 5.3	3.8 ± 0.0	UND	UND	UND	8.2 ± 0.0		15.3 ± 0.0	2.3 ± 0.3	0.7 ± 0.1	0.30 ± 0.06	0.92
		450	98.0 ± 4.6	5.1 ± 1.0	UND	UND	UND	8.9 ± 0.6		29.1 ± 3.9	5.4 ± 0.7	1.0 ± 0.0		
	**mASW.P** ^−^	240	45.2 ± 5.4	10.4 ± 0.5	UND	UND	UND	13.0 ± 1.1		28.1 ± 4.0	10.2 ± 0.9	1.7 ± 0.1	0.26 ± 0.08	0.81
		450	125.3 ± 4.1	6.0 ± 2.5	UND	UND	UND	11.5 ± 2.6		45.9 ± 6.4	15.4 ± 8.8	2.8 ± 1.5		
**b**	**Growth** **medium**	**t (h)**	**Lipid** **fraction**	**Fatty acid composition of total lipids and their fractions (%, wt/wt)**										
				**14:0**	**14:1(n-5)**	**16:0**	**16:1(n-7)**	**17:0**	**18:0**	**18:1(n-9)**	**18:2(n-6)**	**18:3(n-3)**	**18:4(n-3)**	* Others
**Balanced** **growth**	**Control**	240	TL	4.8 ± 0.7	2.7 ± 0.5	14.3 ± 0.9	2.3 ± 0.6	2.9 ± 0.7	7.5 ± 0.3	20.9 ± 2.4	4.8 ± 0.1	22.1 ± 2.4	4.5 ± 4.0	2.7 ± 0.5
		450	TL	1.7 ± 0.2	6.9 ± 0.6	17.1 ± 1.8	3.0 ± 0.3	<0.5	7.3 ± 1.4	14.7 ± 0.1	21.4 ± 0.8	23.0 ± 0.6	0.5 ± 0.0	6.9 ± 0.6
			N	5.6 ± 0.3	26.7 ± 2.9	10.6 ± 1.2	2.1 ± 0.3	1.6 ± 0.3	3.1 ± 0.2	9.2 ± 2.4	18.0 ± 2.0	9.5 ± 0.2	1.4 ± 0.5	26.7 ± 2.9
			G	<0.5	<0.5	18.6 ± 0.2	2.1 ± 0.1	ND	13.6 ± 0.0	14.4 ± 0.6	27.5 ± 0.0	20.3 ± 0.3	3.3 ± 0.5	<0.5
			P	1.0 ± 0.3	11.4 ± 2.6	11.8 ± 3.6	3.3 ± 1.0	1.3 ± 0.1	5.8 ± 2.4	12.5 ± 0.8	37.5 ± 2.0	17.7 ± 1.1	1.9 ± 0.8	11.4 ± 2.6
**Nutrient** **limitation**	**mASW.N** ^−^	240	TL	0.5 ± 0.0	6.7 ± 0.4	15.2 ± 0.4	3.8 ± 0.9	6.1 ± 1.9	10.9 ± 2.7	23.3 ± 1.9	19.1 ± 8.3	13.8 ± 0.6	ND	3.7 ± 1.0
		450	TL	1.1 ± 0.1	7.9 ± 0.3	14.9 ± 0.3	3.7 ± 0.1	7.2 ± 1.1	2.6 ± 0.2	17.6 ± 3.8	23.1 ± 4.0	13.6 ± 0.4	4.6 ± 0.6	3.9 ± 1.2
	**mASW.P** ^−^	240	TL	5.5 ± 1.0	7.6 ± 0.5	17.4 ± 2.1	3.5 ± 0.1	3.6 ± 0.0	5.9 ± 2.5	17.3 ± 2.7	25.4 ± 4.9	13.2 ± 3.2	ND	4.6 ± 0.0
		450	TL	7.7 ± 3.5	10.3 ± 1.8	8.2 ± 0.0	2.9 ± 0.2	10.2 ± 2.2	15.2 ± 1.7	22.1 ± 3.5	7.9 ± 0.0	18.3 ± 0.0	ND	2.8 ± 0.0

Abbreviations: (a) x (mg/L), dry biomass; L/x (%), lipids on dry biomass; N (%); neutral lipid fraction of total lipids; G + S (%); glycolipid and sphingolipid fraction of total lipids; P (%), fraction of phospholipids on total lipids; S/x (%), intracellular polysaccharides on dry biomass; P/x (%), intracellular proteins on dry biomass; TCh/x (%), total chlorophyll (chlorophyll a and b) on dry biomass; TC/x (%), total carotenoids on dry biomass; μ (1/d), maximum specific growth rate; R^2^, R-squared statistical measure; UND, undetermined. (b) ND: not detected, * Others: mainly 10:0, 12:0 and in some cases 18:3(n-6) Note: only glycolipids (i.e., G fraction) are mentioned, as the amide bond of sphingolipids resists methanolysis during methyl esterification.

**Table 4:** (a) Biomass production, reserve materials accumulation, and growth parameters, (b) fatty acid composition of total lipids (TLs) and their lipid fractions (neutral—N, glycolipids—G, and phospholipids—P) of *Picochlorum oklahomense* SAG4.4 grown in modified Artificial Seawater (mASW) and the different variations in growth in an 8.7 L (V_w_ = 5 L) capacity Open Pond Simulating Reactor (OPSR).

**a**	**Growth** **Medium**	**t** **(h)**	**Biomass** **(x)**	**Lipids** **(L)**				**Polysaccharides** **(S)**			**Proteins** **(P)**	**Pigments**			**Growth Parameters**		
			**x** **(mg/L)**	**L/x** **(%)**	**Lipid Fractions (%)**			**S/x** **(%)**			**P/x** **(%)**	**TCh/x** **(%)**	**TC/x** **(%)**		**μ** **(1/d)**		**R^2^**
					**N**	**G + S**	**P**										
**Balanced growth**	**Control**	240	90.7 ± 4.9	8.6 ± 2.0	UND	UND	UND	11.2 ± 0.8			35.0 ± 1.9	1.5 ± 0.1	0.3 ± 0.0		0.21 ± 0.06		0.87
		450	299.3 ± 13.6	9.4 ± 1.5	16.9 ± 0.9	68.7 ± 0.3	15.4 ± 1.1	13.1 ± 0.7			43.8 ± 2.3	4.7 ± 1.1	1.3 ± 0.0				
**Nutrient** **limitation**	**mASW.N** ^−^	240	80.4 ± 5.9	2.1 ± 0.4	UND	UND	UND	11.9 ± 2.7			8.9 ± 3.5	2.0 ± 1.8	0.4 ± 0.3		0.13 ± 0.07		0.90
		450	195.7 ± 14.4	1.2 ± 0.0	UND	UND	UND	10.9 ± 1.1			14.2 ± 4.0	3.9 ± 0.2	0.8 ± 0.0				
	**mASW.P** ^−^	240	95.9 ± 12.3	7.6 ± 3.0	UND	UND	UND	13.0 ± 1.1			29.5 ± 4.2	3.3 ± 0.3	0.7 ± 0.2		0.20 ± 0.03		0.95
		450	529.0 ± 52.2	5.7 ± 1.6	26.1 ± 2.5	67.4 ± 3.7	5.1 ± 0.3	10.6 ± 1.5			38.2 ± 1.4	2.6 ± 0.2	0.5 ± 0.0				
**b**	**Growth** **medium**	**t (h)**	**Lipid** **fraction**	**Fatty acid composition of total lipids and their fractions (%, wt/wt)**													
				**14:0**	**14:1(n-5)**	**16:0**	**16:1(n-7)**	**17:0**	**18:0**	**18:1(n-9)**	**18:2(n-6)**	**18:3(n-6)**		**18:3 (n-3)**	**18:4(n-3)**	**20:1(n-9)**	* Others
**Balanced** **growth**	**Control**	240	TL	4.1 ± 1.9	2.2 ± 0.6	13.4 ± 0.1	1.7 ± 0.1	2.2 ± 0.6	4.7 ± 1.1	18.4 ± 0.7	4.7 ± 1.8	7.6 ± 1.0		19.7 ± 5.0	8.5 ± 2.4	ND	12.8 ± 1.7
		450	TL	1.7 ± 0.2	6.9 ± 0.6	17.1 ± 1.8	3.0 ± 0.3	<0.5	7.3 ± 1.4	14.7 ± 0.1	21.4 ± 0.8	<0.5		23.0 ± 0.6	1.0 ± 0.0	ND	4.4 ± 0.3
			N	16.4 ± 0.4	13.1 ± 0.4	14.1 ± 0.5	3.1 ± 0.1	2.1 ± 0.0	0.8 ± 0.0	11.5 ± 0.4	17.3 ± 0.8	4.3 ± 0.2		13.2 ± 1.0	0.6 ± 0.2	1.6 ± 0.3	2.6 ± 0.1
			G	0.5 ± 0.1	0.6 ± 0.1	18.8 ± 0.6	2.4 ± 0.0	<0.5	11.0 ± 0.1	19.0 ± 0.5	21.0 ± 0.7	ND		24.4 ± 1.5	2.1 ± 0.0	ND	<0.5
			P	0.6 ± 0.1	0.8 ± 0.2	26.5 ± 0.0	5.6 ± 0.5	0.9 ± 0.2	2.5 ± 0.3	15.9 ± 1.7	26.9 ± 0.6	ND		16.3 ± 1.1	2.8 ± 0.0	ND	1.2 ± 0.5
**Nutrient** **limitation**	**mASW.N** ^−^	240	TL	3.6 ± 0.4	7.6 ± 2.2	18.3 ± 1.6	7.0 ± 1.1	7.2 ± 1.2	2.6 ± 1.7	9.0 ± 0.9	5.7 ± 0.5	ND		10.3 ± 0.0	ND	ND	33.8 ± 4.9
		450	TL	2.5 ± 0.0	10.1 ± 0.1	15.2 ± 0.9	4.7 ± 0.7	7.6 ± 0.1	3.1 ± 0.1	17.0 ± 0.5	19.9 ± 0.5	ND		13.5 ± 0.5	ND	ND	6.4 ± 0.5
	**mASW.P** ^−^	240	TL	1.1 ± 0.6	4.8 ± 1.8	17.7 ± 2.2	3.7 ± 1.2	4.4 ± 1.1	0.7 ± 0.1	18.2 ± 1.4	21.1 ± 1.5	1.7 ± 0.0		19.3 ± 0.2	3.2 ± 0.4	ND	4.9 ± 1.3
		450	TL	2.6 ± 1.1	10.1 ± 1.9	13.4 ± 6.1	2.6 ± 0.2	5.8 ± 0.3	0.6 ± 0.0	15.3 ± 2.6	21.1 ± 3.2	ND		19.7 ± 3.2	2.4 ± 1.5	ND	6.4 ± 2.5
			N	2.6 ± 0.2	10.7 ± 0.1	17.7 ± 0.3	3.5 ± 0.3	5.9 ± 0.7	<0.5	14.4 ± 2.0	20.9 ± 0.4	ND		18.3 ± 0.4	<0.5	ND	5.9 ± 0.2
			G	0.5 ± 0.2	1.3 ± 0.1	15.5 ± 0.2	2.7 ± 0.3	13.2 ± 0.2	<0.5	16.9 ± 0.6	23.7 ± 0.1	ND		22.1 ± 0.1	3.0 ± 0.5	ND	1.3 ± 0.3
			P	<0.5	<0.5	20.4 ± 1.1	5.0 ± 0.1	1.5 ± 0.0	4.8 ± 0.2	14.3 ± 0.3	32.7 ± 1.3	ND		17.7 ± 0.7	<0.5	ND	2.5 ± 0.2

Abbreviations: (a) x (mg/L), dry biomass; L/x (%), lipids on dry biomass; N (%), neutral lipid fraction of total lipids; G + S (%), glycolipid and sphingolipid fraction of total lipids; P (%), fraction of phospholipids on total lipids; S/x (%), intracellular polysaccharides on dry biomass; P/x (%), intracellular proteins on dry biomass; TCh/x (%), total chlorophyll (chlorophyll a and b) on dry biomass; TC/x (%), total carotenoids on dry biomass; μ (1/d), maximum specific growth rate; R^2^, R-squared statistical measure; UND, undetermined. (b) ND: not detected. * Others: mainly 10:0, 12:0. Note: only glycolipids (i.e., G fraction) are mentioned, as the amide bond of sphingolipids resists methanolysis during methyl esterification.

**Table 5:** (a) Biomass production, reserve materials accumulation, and growth parameters, (b) fatty acid composition of total lipids (TLs) and their lipid fractions (neutral—N, glycolipids—G, and phospholipids—P) of *Microchloropsis gaditana* VON5.3 grown in modified Artificial Seawater (mASW) and the different variations in growth in an 8.7 L (V_w_ = 5 L) capacity Open Pond Simulating Reactor (OPSR).

**a**	**Growth** **Medium**	**t** **(h)**	**Biomass** **(x)**	**Lipids** **(L)**				**Polysaccharides** **(S)**		**Proteins** **(P)**	**Pigments**		**Growth** **Parameters**			
			**x** **(mg/L)**	**L/x** **(%)**	**Lipid Fractions (%)**			**S/x** **(%)**		**P/x** **(%)**	**TCh/x** **(%)**	**TC/x** **(%)**	**μ** **(1/d)**		**R^2^**	
					**N**	**G + S**	**P**									
**Balanced growth**	**Control**	240	109.3 ± 36.5	10.7 ± 2.7	UND	UND	UND	7.0 ± 0.4		18.7 ± 0.5	3.2 ± 0.8	1.5 ± 0.2	0.25 ± 0.05		0.95	
		450	523.0 ± 136.0	11.8 ± 3.5	34.4 ± 9.1	56.9 ± 5.7	5.4 ± 0.1	9.7 ± 0.4		21.9 ± 4.9	0.4 ± 0.1	0.6 ± 0.4				
**Nutrient** **limitation**	**mASW.N** ^−^	240	97.4 ± 24.6	10.1 ± 0.8	UND	UND	UND	8.7 ± 1.1		12.5 ± 1.2	3.3 ± 0.2	1.3 ± 0.2	0.24 ± 0.04		0.94	
		450	393.9 ± 22.1	11.9 ± 0.3	15.2 ± 4.2	71.5 ± 4.0	13.4 ± 0.2	10.7 ± 0.2		16.2 ± 2.0	2.8 ± 1.8	1.3 ± 0.3				
	**mASW.P** ^−^	240	220.7 ± 1.0	10.2 ± 2.6	UND	UND	UND	7.3 ± 0.2		17.1 ± 4.5	0.8 ± 0.1	0.8 ± 0.1	0.20 ± 0.04		0.94	
		450	392.2 ± 38.0	19.2 ± 2.2	27.2 ± 9.3	55.2 ± 8.6	17.6 ± 0.6	9.7 ± 0.4		35.1 ± 3.5	0.2 ± 0.0	0.3 ± 0.1				
**b**	**Growth** **medium**	**t (h)**	**Lipid** **fraction**	**Fatty acid composition of total lipids and their fractions (%, wt/wt)**												
				**14:0**	**14:1(n-5)**	**16:0**	**16:1(n-7)**	**17:0**	**18:0**	**18:1(n-9)**	**18:2(n-6)**	**18:3(n-3)**	**18:4(n-3)**	**20:1(n-9)**	**20:5(n-3)**	* Others
**Balanced** **growth**	**Control**	240	TL	6.9 ± 1.2	1.9 ± 1.0	23.2 ± 0.0	28.5 ± 3.6	<0.5	0.8 ± 0.4	7.5 ± 0.2	1.9 ± 0.6	1.8 ± 0.8	<0.5	3.5 ± 1.0	17.6 ± 1.4	6.2 ± 3.4
		450	TL	6.6 ± 0.9	3.8 ± 0.9	22.8 ± 0.4	31.3 ± 0.8	<0.5	<0.5	6.8 ± 0.9	2.0 ± 0.5	0.7 ± 0.2	<0.5	2.9 ± 0.4	20.0 ± 1.0	2.5 ± 0.3
			N	5.7 ± 1.4	3.0 ± 1.2	28.6 ± 0.4	34.9 ± 2.7	0.5 ± 0.3	0.6 ± 0.1	8.1 ± 1.2	1.4 ± 0.1	ND	1.8 ± 0.6	2.5 ± 0.6	8.5 ± 1.0	4.9 ± 0.3
			G	9.1 ± 0.0	5.6 ± 0.8	20.8 ± 2.0	28.1 ± 2.8	<0.5	ND	6.4 ± 1.1	1.3 ± 0.4	ND	ND	2.0 ± 0.7	22.3 ± 3.0	4.1 ± 0.5
			P	2.4 ± 0.1	0.6 ± 0.0	21.9 ± 0.2	31.8 ± 0.8	0.8 ± 0.3	<0.5	16.3 ± 1.5	4.4 ± 0.1	0.6 ± 0.2	ND	3.5 ± 0.9	15.7 ± 0.7	2.0 ± 0.4
**Nutrient limitation**	**mASW.N** ^−^	240	TL	7.1 ± 0.0	3.6 ± 0.1	21.5 ± 3.3	28.7 ± 2.1	<0.5	<0.5	5.8 ± 0.1	2.2 ± 0.7	ND	ND	2.8 ± 0.0	20.9 ± 3.7	8.3 ± 1.4
		450	TL	7.6 ± 0.5	3.7 ± 0.0	23.0 ± 1.8	26.9 ± 0.3	<0.5	<0.5	10.0 ± 4.1	1.4 ± 0.2	<0.5	ND	2.9 ± 0.1	20.8 ± 3.7	3.2 ± 1.8
			N	8.3 ± 0.6	7.0 ± 0.5	26.2 ± 0.5	32.2 ± 0.6	0.5 ± 0.3	3.6 ± 2.8	7.2 ± 0.8	2.0 ± 0.6	ND	ND	2.8 ± 0.0	9.2 ± 1.3	1.1 ± 0.4
			G	10.1 ± 0.2	4.1 ± 0.2	22.8 ± 0.1	30.0 ± 0.9	0.6 ± 0.3	0.6 ± 0.0	4.3 ± 0.8	1.6 ± 0.3	ND	ND	2.2 ± 0.0	22.6 ± 0.1	3.8 ± 0.7
			P	3.2 ± 0.0	1.1 ± 0.1	23.7 ± 0.8	30.8 ± 0.1	0.5 ± 0.1	<0.5	15.9 ± 1.2	3.5 ± 0.2	0.6 ± 0.0	ND	4.6 ± 1.1	15.1 ± 1.7	0.8 ± 0.3
	**mASW.P** ^−^	240	TL	6.8 ± 1.5	3.6 ± 0.2	21.8 ± 1.3	28.7 ± 0.0	<0.5	<0.5	7.3 ± 4.0	2.7 ± 0.8	<0.5	0.9 ± 0.1	2.8 ± 0.6	20.6 ± 1.4	3.8 ± 1.2
		450	TL	6.9 ± 0.1	4.8 ± 0.2	19.9 ± 0.9	29.1 ± 0.4	<0.5	<0.5	6.4 ± 2.0	1.8 ± 0.6	0.7 ± 0.3	0.6 ± 0.0	4.3 ± 0.1	22.9 ± 2.9	2.3 ± 0.9
			N	5.8 ± 0.2	4.7 ± 0.3	29.9 ± 1.2	29.4 ± 1.1	0.5 ± 0.0	1.2 ± 0.1	11.4 ± 0.8	1.7 ± 0.2	ND	2.6 ± 1.0	2.9 ± 0.3	7.9 ± 1.1	2.4 ± 0.4
			G	9.0 ± 0.0	4.6 ± 1.0	19.9 ± 2.9	27.3 ± 0.4	<0.5	<0.5	7.7 ± 0.3	1.4 ± 0.4	<0.5	<0.5	3.0 ± 0.3	24.3 ± 1.8	2.3 ± 0.2
			P	2.7 ± 0.1	1.0 ± 0.0	18.2 ± 0.7	25.4 ± 0.5	<0.5	0.6 ± 0.1	13.9 ± 2.1	5.6 ± 2.0	2.1 ± 0.1	<0.5	7.4 ± 1.9	22.1 ± 3.0	0.8 ± 0.2

Abbreviations: (a) x (mg/L), dry biomass; L/x (%), lipids on dry biomass; N (%), neutral lipid fraction of total lipids; G + S (%), glycolipid and sphingolipid fraction of total lipids; P (%), fraction of phospholipids on total lipids; S/x (%), intracellular polysaccharides on dry biomass; P/x (%), intracellular proteins on dry biomass; TCh/x (%), total chlorophyll (chlorophyll a and b) on dry biomass; TC/x (%), total carotenoids on dry biomass; μ (1/d), maximum specific growth rate; R^2^, R-squared statistical measure; UND, undetermined. (b) ND: not detected. * Others: mainly 10:0, 12:0, and in some cases 18:3(n-6) Note: only glycolipids (i.e., G fraction) are mentioned, as the amide bond of sphingolipids resists methanolysis during methyl esterification.

**Table 6:** (a) Biomass production, reserve materials accumulation, and growth parameters, (b) fatty acid composition of total lipids (TLs) and their lipid fractions (neutral—N, glycolipids—G, and phospholipids—P) of *Nephroselmis pyriformis* PAT2.7 grown in modified Artificial Seawater (mASW) and the different variations in growth in an 8.7 L (V_w_ = 5 L) capacity Open Pond Simulating Reactor (OPSR).

**a**	**Growth** **Medium**	**t** **(h)**	**Biomass** **(x)**	**Lipids** **(L)**				**Polysaccharides** **(S)**	**Proteins** **(P)**	**Pigments**		**Growth** **Parameters**	
			**x** **(mg/L)**	**L/x** **(%)**	**Lipid Fractions (%)**			**S/x** **(%)**	**P/x** **(%)**	**TCh/x** **(%)**	**TC/x** **(%)**	**μ** **(1/d)**	**R^2^**
					**N**	**G + S**	**P**						
**Balanced growth**	**Control**	240	137.9 ± 32.8	6.7 ± 0.2	UND	UND	UND	11.6 ± 1.1	21.8 ± 1.8	1.4 ± 0.1	0.4 ± 0.0	0.22 ± 0.03	0.95
		450	471.4 ± 27.6	5.5 ± 1.6	41.5 ± 1.5	53.6 ± 3.1	5.0 ± 1.8	15.3 ± 0.9	37.8 ± 3.8	0.8 ± 0.1	0.2 ± 0.0		
**Nutrient** **limitation**	**mASW.N** ^−^	240	122.1 ± 15.4	3.6 ± 0.1	UND	UND	UND	11.9 ± 0.6	25.2 ± 2.2	3.5 ± 1.1	0.8 ± 0.3	** 0.24 ± 0.07	0.94
		450	162.0 ± 53.0	6.2 ± 1.6	UND	UND	UND	31.6 ± 2.4	23.1 ± 3.7	1.5 ± 0.5	0.5 ± 0.2		
	**mASW.P** ^−^	240	386.6 ± 7.6	2.5 ± 0.0	UND	UND	UND	16.2 ± 0.0	12.0 ± 0.0	0.9 ± 0.1	0.2 ± 0.0	0.25 ± 0.06	0.92
		450	452.2 ± 21.0	1.7 ± 0.1	UND	UND	UND	21.8 ± 0.7	22.1 ± 0.3	2.3 ± 0.0	0.0 ± 0.0		
**b**	**Growth** **medium**	**t (h)**	**Lipid** **fraction**	**Fatty acid composition of total lipids and their fractions (%, wt/wt)**									
				**14:0**	**14:1(n-5)**	**16:0**	**16:1(n-7)**	**18:0**	**18:1(n-9)**	**18:2(n-6)**		* Others	
**Balanced** **growth**	**Control**	240	TL	30.2 ± 0.2	8.3 ± 0.6	9.8 ± 0.0	40.0 ± 4.1	2.6 ± 1.6	5.6 ± 1.7	1.9 ± 0.0		2.4 ± 0.3	
		450	TL	31.0 ± 0.7	4.4 ± 1.1	9.4 ± 0.9	39.0 ± 3.9	2.7 ± 2.0	5.4 ± 2.2	0.8 ± 0.3		7.3 ± 1.8	
			N	34.6 ± 1.2	5.7 ± 0.4	10.0 ± 0.6	41.1 ± 0.9	0.9 ± 0.0	2.3 ± 0.2	1.3 ± 0.1		7.7 ± 0.4	
			G	31.7 ± 0.2	4.7 ± 1.3	8.6 ± 0.8	39.4 ± 0.3	3.3 ± 2.2	3.9 ± 1.4	0.7 ± 0.4		4.1 ± 0.8	
			P	14.4 ± 1.8	10.0 ± 0.0	13.7 ± 1.1	29.6 ± 0.4	4.7 ± 0.9	17.1 ± 2.4	5.2 ± 2.8		5.8 ± 0.3	
**Nutrient limitation**	**mASW.N** ^−^	240	TL	32.2 ± 1.3	4.5 ± 0.3	11.6 ± 0.4	38.6 ± 3.2	1.3 ± 0.3	2.1 ± 0.3	ND		9.7 ± 3.2	
		450	TL	28.4 ± 3.8	3.4 ± 1.1	13.2 ± 1.6	42.1 ± 0.9	2.4 ± 1.1	4.5 ± 2.1	<0.1		9.7 ± 3.2	
	**mASW.P** ^−^	240	TL	26.8 ± 0.3	3.5 ± 0.2	11.0 ± 0.1	34.9 ± 0.4	2.3 ± 0.7	9.1 ± 0.5	2.8 ± 1.1		9.7 ± 0.1	
		450	TL	23.3 ± 0.8	7.5 ± 0.3	9.9 ± 0.2	40.4 ± 1.3	1.7 ± 0.6	7.4 ± 2.4	1.1 ± 0.0		9.4 ± 0.1	

Abbreviations: (a) x (mg/L), dry biomass; L/x (%), lipids on dry biomass; N (%), neutral lipid fraction of total lipids; G + S (%), glycolipid and sphingolipid fraction of total lipids; P (%), fraction of phospholipids on total lipids; S/x (%), intracellular polysaccharides on dry biomass; P/x (%), intracellular proteins on dry biomass; TCh/x (%), total chlorophyll (chlorophyll a and b) on dry biomass; TC/x (%), total carotenoids on dry biomass; μ (1/d), maximum specific growth rate; R^2^, R-squared statistical measure; UND, undetermined. (b) ND: not detected. * Others: mainly 10:0, 12:0. Note: only glycolipids (i.e., G fraction) are mentioned, as the amide bond of sphingolipids resists methanolysis during methyl esterification. ** Cell count ceased at approximately 330 h of culture due to the increased tendency of the cells to form cell aggregates.

## Data Availability

The original contributions presented in this study are included in the article. Further inquiries can be directed to the corresponding author.

## Supplementary Materials

The following supporting information can be downloaded at: https://www.mdpi.com/article/10.3390/life15091427/s1, Figures S1 and S2; Tables S1–S20.

## Abbreviations

The following abbreviations are used in this manuscript:

PUFAs	Poly-Unsaturated Fatty Acids
EPA	n-3 Eicosapentaenoic Acid
ALA	α-Linolenic Acid
OPSR	Open Pond Simulating Reactor
mASW	Modified Artificial Seawater
mASW.N^−^	Nitrogen-limited growth medium
mASW.P^−^	Phosphorus-limited growth medium
NLs	Neutral Lipids
G + S	Glycolipids + Sphingolipids
P	Phospholipids

## References

[1] Ahiahonu E.K., Anku W.W., Roopnarain A., Green E., Govender P.P., Serepa-Dlamini M.H. (2023). Bioprospecting wild South African microalgae as a potential third-generation biofuel feedstock, biological carbon-capture agent and for nutraceutical applications. Biomass Conv. Bioref..

[2] Keresztes Z.G., Felföldi T., Somogyi B., Székely G., Dragoş N., Márialigeti K., Bartha C., Vörös L. (2012). First record of picophytoplankton diversity in Central European hypersaline lakes. Extremophiles.

[3] Ntzouvaras A., Chantzistrountsiou X., Papageorgiou N., Koletti A., Adamakis I.-D., Zografaki M.-E., Marka S., Vasilakis G., Tsirigoti A., Tzovenis I. (2023). New records of *Tetraselmis* sp. strains with biotechnological potential isolated from Greek coastal lagoons. Water.

[4] Arunachalam Sivagurulingam A.P., Sivanandi P., Pandian S. (2022). Isolation, mass cultivation, and biodiesel production potential of marine microalgae identified from Bay of Bengal. Environ. Sci. Pollut. Res..

[5] Gonzalez-Esquer C.R., Wright K.T., Sudasinghe N., Carr C.K., Sanders C.K., Turmo A., Kerfeld C.A., Twary S., Dale T. (2019). Demonstration of the potential of *Picochlorum soloecismus* as a microalgal platform for the production of renewable fuels. Algal Res..

[6] Barten R., Kleisman M., D’Ermo G., Nijveen H., Wijffels R.H., Barbosa M.J. (2022). Short-term physiologic response of the green microalga *Picochlorum* sp. (BPE23) to supra-optimal temperature. Sci. Rep..

[7] de la Vega M., Díaz E., Vila M., León R. (2011). Isolation of a new strain of *Picochlorum* sp. and characterization of its potential biotechnological applications. Biotechnol. Prog..

[8] Dritsas P., Aggelis G. (2025). Impact of temperature on the biochemical potential of five newly isolated strains of microalgae cultured in a stirred tank reactor. Microorganisms.

[9] Hotos G.N., Avramidou D. (2021). The effect of various salinities and light intensities on the growth performance of five locally isolated microalgae [*Amphidinium carterae*, *Nephroselmis* sp., *Tetraselmis* sp. (Var. Red Pappas), *Asteromonas gracilis* and *Dunaliella* sp.] in laboratory batch cultures. J. Mar. Sci. Eng..

[10] Weissman J.C., Likhogrud M., Thomas D.C., Fang W., Karns D.A.J., Chung J.W., Nielsen R., Posewitz M.C. (2018). High-light selection produces a fast-growing *Picochlorum celeri*. Algal Res..

[11] Zhu Y., Dunford N.T. (2013). Growth and biomass characteristics of *Picochlorum oklahomensis* and *Nannochloropsis oculata*. J. Am. Oil Chem. Soc..

[12] Díaz N., Muñoz S., Medina A., Riquelme C., Lozano-Muñoz I. (2025). *Microchloropsis gaditana* as a natural antimicrobial with a one health approach to food safety in farmed salmon. Life.

[13] Dritsas P., Patsialou S., Kampantais D., Roussos E., Kotzamanis Y., Tekerlekopoulou A., Vayenas D.V., Aggelis G. (2025). Investigating the potential of newly isolated microalgae strains from the Ionian Sea (Greece) cultured in an open raceway pond. Appl. Sci..

[14] Grubišić M., Šantek B., Zorić Z., Čošić Z., Vrana I., Gašparović B., Čož-Rakovac R., Šantek M.I. (2022). Bioprospecting of microalgae isolated from the Adriatic Sea: Characterization of biomass, pigment, lipid and fatty acid composition, and antioxidant and antimicrobial activity. Molecules.

[15] Kang S., Shin H.H., Li Z. (2024). The discovery and characterization of a novel microalgal strain, *Picochlorum* sp. KCTC AG61293, with potential for α-linolenic acid production. J. Mar. Sci. Eng..

[16] Blondeau N., Lipsky R.H., Bourourou M., Duncan M.W., Gorelick P.B., Marini A.M. (2015). Alpha-linolenic acid: An 1323 omega-3 fatty acid with neuroprotective properties—Ready for use in the stroke clinic?. BioMed Res. Int..

[17] Nassar M., Jaffery A., Ibrahim B., Baraka B., Abosheaishaa H. (2023). The multidimensional benefits of eicosapentaenoic acid: From heart health to inflammatory control. Egypt. J. Intern. Med..

[18] Sala-Vila A., Fleming J., Kris-Etherton P., Ros E. (2022). Impact of α-linolenic acid, the vegetable ω-3 fatty acid, on cardiovascular disease and cognition. Adv. Nutr..

[19] Ahn Y., Park S., Ji M.K., Ha G.S., Jeon B.H., Choi J. (2022). Biodiesel production potential of microalgae, cultivated in acid mine drainage and livestock wastewater. J. Environ. Manag..

[20] Dritsas P., Asimakis E., Lianou A., Efstratiou M., Tsiamis G., Aggelis G. (2023). Microalgae from the Ionian Sea (Greece): Isolation, molecular identification and biochemical features of biotechnological interest. Algal Res..

[21] Mastropetros S.G., Tsigkou K., Cladas Y., Priya A.K., Kornaros M. (2023). Effect of nitrogen, salinity, and light intensity on the biomass composition of *Nephroselmis* sp.: Optimization of lipids accumulation (including EPA). Mar. Drugs.

[22] Qazi W.M., Ballance S., Kousoulaki K., Uhlen A.K., Kleinegris D.M.M., Skjånes K., Rieder A. (2021). Protein enrichment of wheat bread with microalgae: *Microchloropsis gaditana*, *Tetraselmis chui* and *Chlorella vulgaris*. Foods.

[23] Coulombier N., Nicolau E., Déan L.L., Antheaume C., Jauffrais T., Lebouvier N. (2020). Impact of light intensity on antioxidant activity of tropical microalgae. Mar. Drugs.

[24] Zanella L., Vianello F. (2020). Microalgae of the genus *Nannochloropsis*: Chemical composition and functional implications for human nutrition. J. Funct. Foods.

[25] Chowdury K.H., Nahar N., Deb U.K. (2020). The growth factors involved in microalgae cultivation for biofuel production: A review. Comput. Water Energy Environ. Eng..

[26] Dourou M., Dritsas P., Baeshen M.N., Elazzazy A., Al-Farga A., Aggelis G. (2021). High-added value products from microalgae and prospects of aquaculture wastewaters as microalgae growth media. FEMS Microbiol. Lett..

[27] Khan M.I., Shin J.H., Kim J.D. (2018). The promising future of microalgae: Current status, challenges, and optimization of a sustainable and renewable industry for biofuels, feed, and other products. Microb. Cell Fact..

[28] Lu Q., Li H., Xiao Y., Liu H. (2021). A state-of-the-art review on the synthetic mechanisms, production technologies, and practical application of polyunsaturated fatty acids from microalgae. Algal Res..

[29] Folch J., Lees M., Stanley G.H.S. (1957). A simple method for the isolation and purification of total lipides from animal tissues. J. Biol. Chem..

[30] Dourou M., Tsolcha O.N., Tekerlekopoulou A.G., Bokas D., Aggelis G. (2018). Fish farm effluents are suitable growth media for *Nannochloropsis gaditana*, a polyunsaturated fatty acid producing microalga. Eng. Life Sci..

[31] Bellou S., Aggelis G. (2012). Biochemical activities in *Chlorella* sp. and *Nannochloropsis salina* during lipid and sugar synthesis in a lab-scale open pond simulating reactor. J. Biotechnol..

[32] Miller G.L. (1959). Use of dinitrosalicylic acid reagent for determination of reducing sugar. Anal. Chem..

[33] Sumanta N., Haque C.I., Nishika J., Suprakash R. (2014). Spectrophotometric analysis of chlorophylls and carotenoids from commonly grown fern species by using various extracting solvents. Res. J. Chem. Sci..

[34] Cecchin M., Berteotti S., Paltrinieri S., Vigliante I., Iadarola B., Giovannone B., Maffei M.E., Delledonne M., Ballottari M. (2020). Improved lipid productivity in *Nannochloropsis gaditana* in nitrogen-replete conditions by selection of pale green mutants. Biotechnol. Biofuels.

[35] Bartley M.L., Boeing W.J., Dungan B.N., Holguin F.O., Schaub T. (2014). pH effects on growth and lipid accumulation of the biofuel microalgae *Nannochloropsis salina* and invading organisms. J. Appl. Phycol..

[36] Moheimani N.R. (2013). Inorganic carbon and pH effect on growth and lipid productivity of *Tetraselmis suecica* and *Chlorella* sp. (Chlorophyta) grown outdoors in bag photobioreactors. J. Appl. Phycol..

[37] Jui T.J., Tasnim A., Islam S.M.R., Manjur O.H.B., Hossain M.S., Tasnim N., Karmakar D., Hasan M.R., Karim M.R. (2024). Optimal growth conditions to enhance *Chlorella vulgaris* biomass production in indoor phyto tank and quality assessment of feed and culture stock. Heliyon.

[38] Minhas A.K., Gaur S., Adholeya A. (2023). Influence of light intensity and photoperiod on the pigment and, lipid production of *Dunaliella tertiolecta* and *Nannochloropsis oculata* under three different culture medium. Heliyon.

[39] El-Kassas H.Y. (2013). Growth and fatty acid profile of the marine microalga *Picochlorum* sp. grown under nutrient stress conditions. Egypt. J. Aquat. Res..

[40] Abdelkarim O.H., Verhagen R.A., Wijffels R.H., Barbosa M.J. (2024). Physiological, biochemical, and morphological responses to nitrogen starvation and biomass-specific photon supply rates of *Nannochloropsis oceanica* and *Microchloropsis gaditana*. J. Appl. Phycol..

[41] Carvalho A.P., Silva S.O., Baptista J.M., Malcata F.X. (2011). Light requirements in microalgal photobioreactors: An overview of biophotonic aspects. Appl. Microbiol. Biotechnol..

[42] Saccardo A., Bezzo F., Sforza E. (2022). Microalgae growth in ultra-thin steady-state continuous photobioreactors: Assessing self-shading effects. Front. Bioeng. Biotechnol..

[43] Yen H.-W., Chiang W.-C. (2012). Effects of mutual shading, pressurization and oxygen partial pressure on the autotrophical cultivation of *Scenedesmus obliquus*. J. Taiwan Inst. Chem. Eng..

[44] Nguyen P.T.H., Cao P., Vo T. (2024). Effect of phosphorus on the growth, pigmentation and lipid accumulation in microalgae *Picochlorum* sp.. Eur. J. Appl. Sci. Eng. Technol..

[45] Vo T., Cao P., Nguyen P.T.H. (2023). Effect of nitrate on the growth and lipid accumulation in microalgae *Picochlorum* sp.. J. Basic Appl. Res. Int..

[46] Anto S., Premalatha M., Mathimani T. (2024). N:P Ratio and salinity as keys: A study on optimizing biomass and lipid production in marine *Chlorella* sp. NITT 02 and *Picochlorum* sp. NITT 04 for biodiesel production. Biomass Bioenergy.

[47] Kim S.Y., Moon H., Kwon Y.M., Kim K.W., Kim J.Y.H. (2024). Comparative analysis of the biochemical and molecular responses of *Nannochloropsis gaditana* to nitrogen and phosphorus limitation: Phosphorus limitation enhances carotenogenesis. Mar. Drugs.

[48] Rocha J.M.S., Garcia J.E.C., Henriques M.H.F. (2003). Growth aspects of the marine microalga *Nannochloropsis gaditana*. Biomol. Eng..

[49] Mohammady N. (2014). Growth and oil production of *Nannochloropsis salina* cultivated under multiple stressors. J. Pure Appl. Microbiol..

[50] Simionato D., Sforza E., Carpinelli E.C., Bertucco A., Giacometti G.M., Morosinotto T. (2011). Acclimation of *Nannochloropsis gaditana* to different illumination regimes: Effects on lipids accumulation. Bioresour. Technol..

[51] Yun H.S., Lee H., Park Y.T., Ji M.K., Kabra A.N., Jeon C., Jeon B.H., Choi J. (2014). Isolation of novel microalgae from acid mine drainage and its potential application for biodiesel production. Appl. Biochem. Biotechnol..

[52] Subasankari K., Thanappan V., Anantharaman P. (2018). A comparative study on vitamin B12 and co-culture system promotes the growth of microalgae *Nephroselmis astigmatica*. Int. J. Pharm. Biol. Sci..

[53] Dahmen I., Chtourou H., Jebali A., Daassi D., Karray F., Hassairi I., Sayadi S., Abdelkafi S., Dhouib A. (2014). Optimisation of the critical medium components for better growth of *Picochlorum* sp. and the role of stressful environments for higher lipid production. J. Sci. Food Agric..

[54] LaPanse A.J., Krishnan A., Dennis G., Karns D.A.J., Dahlin L.R., Van Wychen S., Burch T.A., Guarnieri M.T., Weissman J.C., Posewitz M.C. (2024). Proximate biomass characterization of the high productivity marine microalga *Picochlorum celeri* TG2. Plant Physiol. Biochem..

[55] Watanabe K., Fujii K. (2016). Isolation of high-level-CO2-preferring *Picochlorum* sp. strains and their biotechnological potential. Algal Res..

[56] Maltsev Y., Maltseva K., Kulikovskiy M., Maltseva S. (2021). Influence of light conditions on microalgae growth and content of lipids, carotenoids, and fatty acid composition. Biology.

[57] Aditi, Bhardwaj R., Yadav A., Swapnil P., Meena M. (2025). Characterization of microalgal β-carotene and astaxanthin: Exploring their health-promoting properties under the effect of salinity and light intensity. Biotechnol. Biofuels Bioprod..

[58] Chin-On R., De Boer M., Van De Voort C., Camstra J., Barbosa M., Wijffels R.H., Janssen M. (2024). Outdoor cultivation of *Picochlorum* sp. in a novel V-shaped photobioreactor on the Caribbean Island Bonaire. Front. Bioeng. Biotechnol..

[59] Nana Annan J. (2014). Growth and photosynthesis response of the green alga, *Picochlorum oklahomensis* to iron limitation and salinity stress. Int. J. Plant Physiol. Biochem..

[60] Magpusao J., Oey I., Kebede B. (2024). Chemical, rheological, and volatile profiling of microalgae *Arthrospira*, *Isochrysis*, *Nannochloropsis*, and *Tetraselmis* species. Food Innov. Adv..

[61] Niccolai A., Chini Zittelli G., Rodolfi L., Biondi N., Tredici M.R. (2019). Microalgae of interest as food source: Biochemical composition and digestibility. Algal Res..

[62] Boussiba S., Vonshak A., Cohen Z., Avissar Y., Richmond A. (1987). Lipid and biomass production by the halotolerant microalga *Nannochloropsis salina*. Biomass.

[63] Hoffmann M., Marxen K., Schulz R., Vanselow K.H. (2010). TFA and EPA productivities of *Nannochloropsis salina* influenced by temperature and nitrate stimuli in turbidostatic controlled experiments. Mar. Drugs.

[64] Jia J., Han D., Gerken H.G., Li Y., Sommerfeld M., Hu Q., Xu J. (2015). Molecular mechanisms for photosynthetic carbon partitioning into storage neutral lipids in *Nannochloropsis oceanica* under nitrogen-depletion conditions. Algal Res..

[65] Rodolfi L., Chini Zittelli G., Bassi N., Padovani G., Biondi N., Bonini G., Tredici M.R. (2009). Microalgae for oil: Strain selection, induction of lipid synthesis and outdoor mass cultivation in a low-cost photobioreactor. Biotech. Bioeng..

[66] Di Lena G., Casini I., Lucarini M., Lombardi-Boccia G. (2019). Carotenoid profiling of five microalgae species from large-scale production. Food Res. Int..

[67] Thinh L.-V., Renaud S.M., Parry D.L. (1999). Evaluation of recently isolated Australian tropical microalgae for the enrichment of the dietary value of brine shrimp, *Artemia nauplii*. Aquaculture.

[68] Hotos G., Avramidou D., Mastropetros S.G., Tsigkou K., Kouvara K., Makridis P., Kornaros M. (2023). Isolation, identification, and chemical composition analysis of nine microalgal and cyanobacterial species isolated in lagoons of Western Greece. Algal Res..

[69] Ji M.-K., Yun H.-S., Hwang B.S., Kabra A.N., Jeon B.-H., Choi J. (2016). Mixotrophic cultivation of *Nephroselmis* sp. using industrial wastewater for enhanced microalgal biomass production. Ecol. Eng..

[70] Hotos G.N., Bekiari V. (2023). Absorption spectra as predictors of algal biomass and pigment content of the cultured microalgae *Amphidinium carterae*, *Isochrysis galbana*, *Nephroselmis* sp., and *Anabaena* sp.. Int. J. Plant Biol..

[71] Dambeck M., Sandmann G. (2014). Antioxidative activities of algal keto carotenoids acting as antioxidative protectants in the chloroplast. Photochem. Photobiol..

[72] Manabe Y., Takii Y., Sugawara T. (2020). Siphonaxanthin, a carotenoid from green algae, suppresses advanced glycation end product-induced inflammatory responses. J. Nat. Med..

[73] Kallau M., Yang H. (2025). Quantification of the total lipids in three aquaculture microalgae using BODIPYTM 505/515 stain and flow cytometry. J. World Aquac. Soc..

[74] Wang X., Wang Y., Zuo L., Guo S., Song P., Kong W., Shen B. (2025). Lipidomic analysis of microalgae and its application in microalgae cultivation and alternative liquid biofuel production. Biomass Conv. Bioref..

[75] Guschina I.A., Harwood J.L. (2006). Lipids and lipid metabolism in eukaryotic algae. Prog. Lipid Res..

[76] Harwood J.L., Guschina I.A. (2009). The versatility of algae and their lipid metabolism. Biochimie.

[77] Zhong Y., Li Y., Xu J., Cao J., Zhou C., Yan X. (2022). Isolation of chloroplasts from marine microalga *Isochrysis galbana* Parke for their lipid composition analysis. J. Ocean. Univ. China.

[78] Fernandes T., Cordeiro N. (2021). Microalgae as sustainable biofactories to produce high-value lipids: Biodiversity, exploitation, and biotechnological applications. Mar. Drugs.

[79] Maltsev Y., Maltseva K. (2021). Fatty acids of microalgae: Diversity and applications. Rev. Environ. Sci. Biotechnol..

[80] Li Y., Lou Y., Mu T., Ke A., Ran Z., Xu J., Chen J., Zhou C., Yan X., Xu Q. (2017). Sphingolipids in marine microalgae: Development and application of a mass spectrometric method for global structural characterization of ceramides and glycosphingolipids in three major phyla. Anal. Chim. Acta.

[81] Miazek K., Lebecque S., Hamaidia M., Paul A., Danthine S., Willems L., Frédérich M., Pauw E.D., Deleu M., Richel A. (2016). Sphingolipids: Promising lipid-class molecules with potential applications for industry. A review. Biotechnol. Agron. Soc. Environ..

[82] Nzayisenga J.C., Farge X., Groll S.L., Sellstedt A. (2020). Effects of light intensity on growth and lipid production in microalgae grown in wastewater. Biotechnol. Biofuels.

[83] Fakas S., Papanikolaou S., Galiotou-Panayotou M., Komaitis M., Aggelis G. (2006). Lipids of *Cunninghamella echinulata* with emphasis to γ-linolenic acid distribution among lipid classes. Appl. Microbiol. Biotechnol..

[84] Khozin-Goldberg I., Cohen Z. (2011). Unraveling algal lipid metabolism: Recent advances in gene identification. Biochimie.

[85] Damiani M.C., Popovich C.A., Constenla D., Leonardi P.I. (2010). Lipid analysis in *Haematococcus pluvialis* to assess its potential use as a biodiesel feedstock. Bioresour. Technol..

[86] Wang X., Shen Z., Miao X. (2016). Nitrogen and hydrophosphate affects glycolipids composition in microalgae. Sci. Rep..

[87] Liang K., Zhang Q., Gu M., Cong W. (2013). Effect of phosphorus on lipid accumulation in freshwater microalga *Chlorella* sp.. J. Appl. Phycol..

[88] Xin L., Hong-ying H., Ke G., Ying-xue S. (2010). Effects of different nitrogen and phosphorus concentrations on the growth, nutrient uptake, and lipid accumulation of a freshwater microalga *Scenedesmus* sp.. Bioresour. Technol..

[89] Benning C., Huang Z.H., Gage D.A. (1995). Accumulation of a novel glycolipid and a betaine lipid in cells of *Rhodobacter sphaeroides* grown under phosphate limitation. Arch. Biochem. Biophys..

[90] Rajaram S. (2014). Health benefits of plant-derived α-linolenic acid. Am. J. Clin. Nutr..

[91] Kris-Etherton P.M., Fleming J.A. (2015). Emerging nutrition science on fatty acids and cardiovascular disease: Nutritionists’ perspectives. Adv. Nutr..

[92] Poli A., Catapano A.L., Corsini A., Manzato E., Werba J.P., Catena G., Cetin I., Cicero A.F.G., Cignarella A., Colivicchi F. (2023). LDL-cholesterol control in the primary prevention of cardiovascular diseases: An expert opinion for clinicians and health professionals. Nutr. Metab. Cardiovasc. Dis..

[93] Dinesh Kumar S., Ananth S., Santhanam P., Parveez Ahamed A., Thajuddin N. (2019). Effect of photoperiod (PP) and photosynthetic photon flux intensity (PPFI) on nutrients consumption, growth and lipid profile of unusual microalga *Picochlorum maculatum* (PSDK01) in shrimp culture effluent. Indian. J. Exp. Biol..

[94] Jesionowska M., Ovadia J., Hockemeyer K., Clews A.C., Xu Y. (2023). EPA and DHA in microalgae: Health benefits, biosynthesis, and metabolic engineering advances. J. Am. Oil Chem. Soc..

[95] Tran D., Giordano M., Louime C., Tran N., Vo T., Nguyen D., Hoang T. (2014). An isolated *Picochlorum* species for aquaculture, food, and biofuel. N. Am. J. Aquac..

[96] Bellou S., Baeshen M.N., Elazzazy A.M., Aggeli D., Sayegh F., Aggelis G. (2014). Microalgal lipids biochemistry and biotechnological perspectives. Biotechnol. Adv..

[97] Chen Y., Mai Q., Chen Z., Lin T., Cai Y., Han J., Wang Y., Zhang M., Tan S., Wu Z. (2023). Dietary palmitoleic acid reprograms gut microbiota and improves biological therapy against colitis. Gut Microbes.

[98] Yan D., Ye S., He Y., Wang S., Xiao Y., Xiang X., Deng M., Luo W., Chen X., Wang X. (2023). Fatty acids and lipid mediators in inflammatory bowel disease: From mechanism to treatment. Front. Immunol..

[99] Hu Q., Xiang W., Dai S., Li T., Yang F., Jia Q., Wang G., Wu H. (2015). The influence of cultivation period on growth and biodiesel properties of microalga *Nannochloropsis gaditana* 1049. Bioresour. Technol..

[100] Castejón N., Marko D. (2022). Fatty acid composition and cytotoxic activity of lipid extracts from *Nannochloropsis gaditana* produced by green technologies. Molecules.

[101] Abdelkarim O.H., Wijffels R.H., Barbosa M.J. (2025). Microalgal lipid production: A comparative analysis of *Nannochlo-ropsis* and *Microchloropsis* strains. J. Appl. Phycol..

[102] Ma Y., Wang Z., Yu C., Yin Y., Zhou G. (2014). Evaluation of the potential of 9 *Nannochloropsis* strains for biodiesel production. Bioresour. Technol..

[103] Van Wagenen J., Miller T.W., Hobbs S., Hook P., Crowe B., Huesemann M. (2012). Effects of light and temperature on fatty acid production in *Nannochloropsis salina*. Energies.

[104] Venkata Subhash G., Rohit M.V., Devi M.P., Swamy Y.V., Venkata Mohan S. (2014). Temperature induced stress influence on biodiesel productivity during mixotrophic microalgae cultivation with wastewater. Bioresour. Technol..

[105] Tian L., Chi G., Lin S., Ling X., He N. (2024). Marine microorganisms: Natural factories for polyunsaturated fatty acid production. Blue Biotechnol..

[106] Alboresi A., Perin G., Vitulo N., Diretto G., Block M., Jouhet J., Meneghesso A., Valle G., Giuliano G., Maréchal E. (2016). Light remodels lipid biosynthesis in *Nannochloropsis gaditana* by modulating carbon partitioning between organelles. Plant Physiol..

