# Supplementary material for: Metabolic Responses of Newly Isolated Microalgal Strains Cultured in an Open Pond Simulating Reactor Under Balanced Conditions and Nutrient Limitation
Source: Life (Basel). 2025 Sep 11;15(9):1427. doi: 10.3390/life15091427 (PMC12471615; doi:10.3390/life15091427)
Supplement: Supplementary file 1 [file life-15-01427-s001.zip › Supplementary Material.v2.pdf]

Article

Metabolic responses of newly isolated microalgal strains cultured in an Open-Pond Simulating Reactor under balanced conditions and nutrient limitation

Panagiotis Dritsas, George Aggelis*

SUPPLEMENTARY MATERIAL

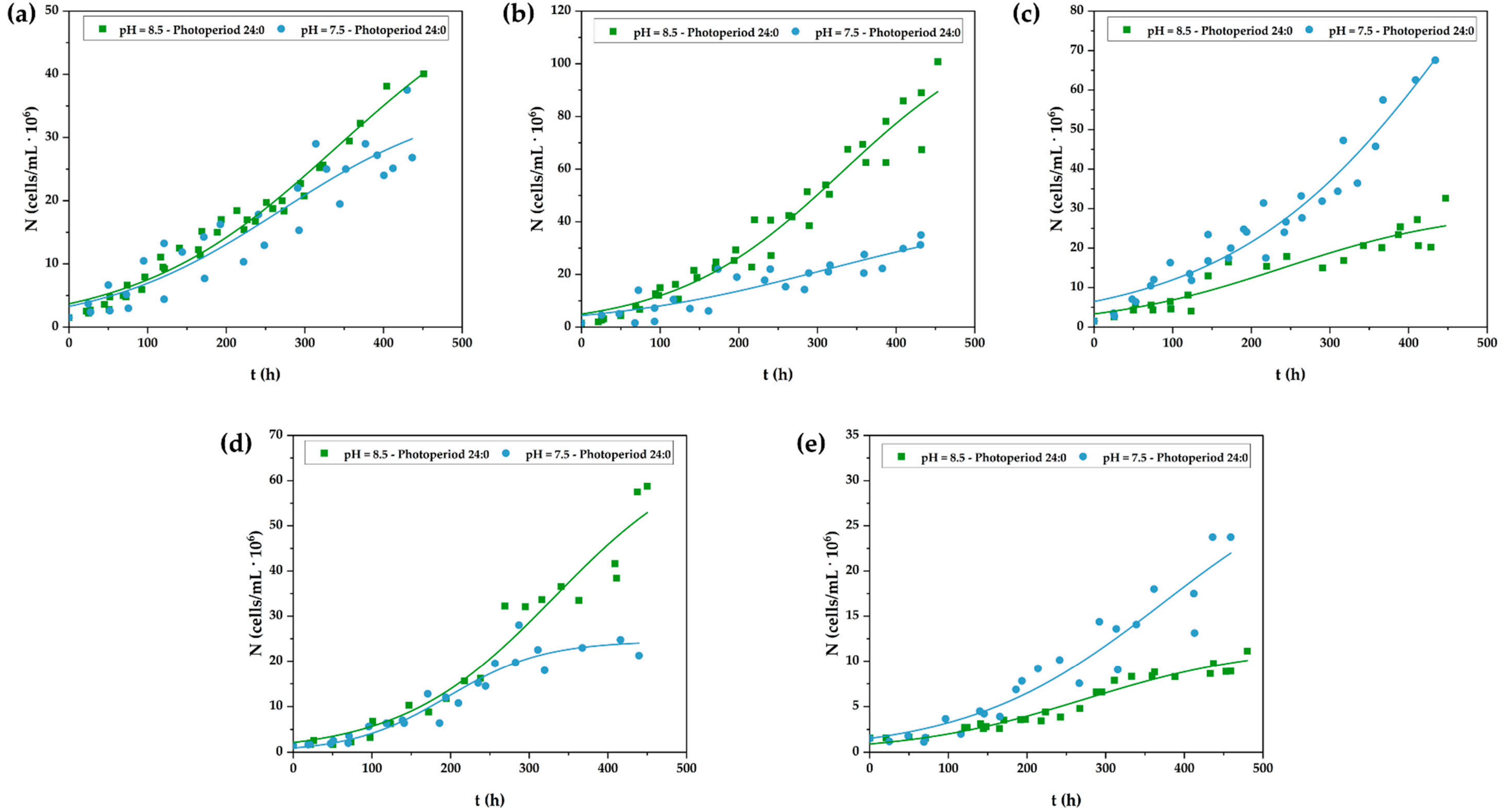

Figure S1. Growth curves of (a) *Picochlorum costavermella* VAS2.5, (b) *Picochlorum oklahomense* PAT3.2B, (c) *Picochlorum oklahomense* SAG4.4, (d) *Microchloropsis gaditana* VON5.3, and (e) *Nephroselmis pyriformis* PAT2.7 cultured in modified Artificial Seawater (mASW) under photoperiod of 24:0 (h light:dark) at pH = 8.5 or pH = 7.5 in an Open-Pond Simulating Reactor (OPSR) of 8.7 L (V_w = 5 L) capacity (two biological replicates). Each point is an average of two measurements and the curves were obtained by fitting the Verhulst model to the experimental data. In the legend of each graph the different culture conditions are denoted.

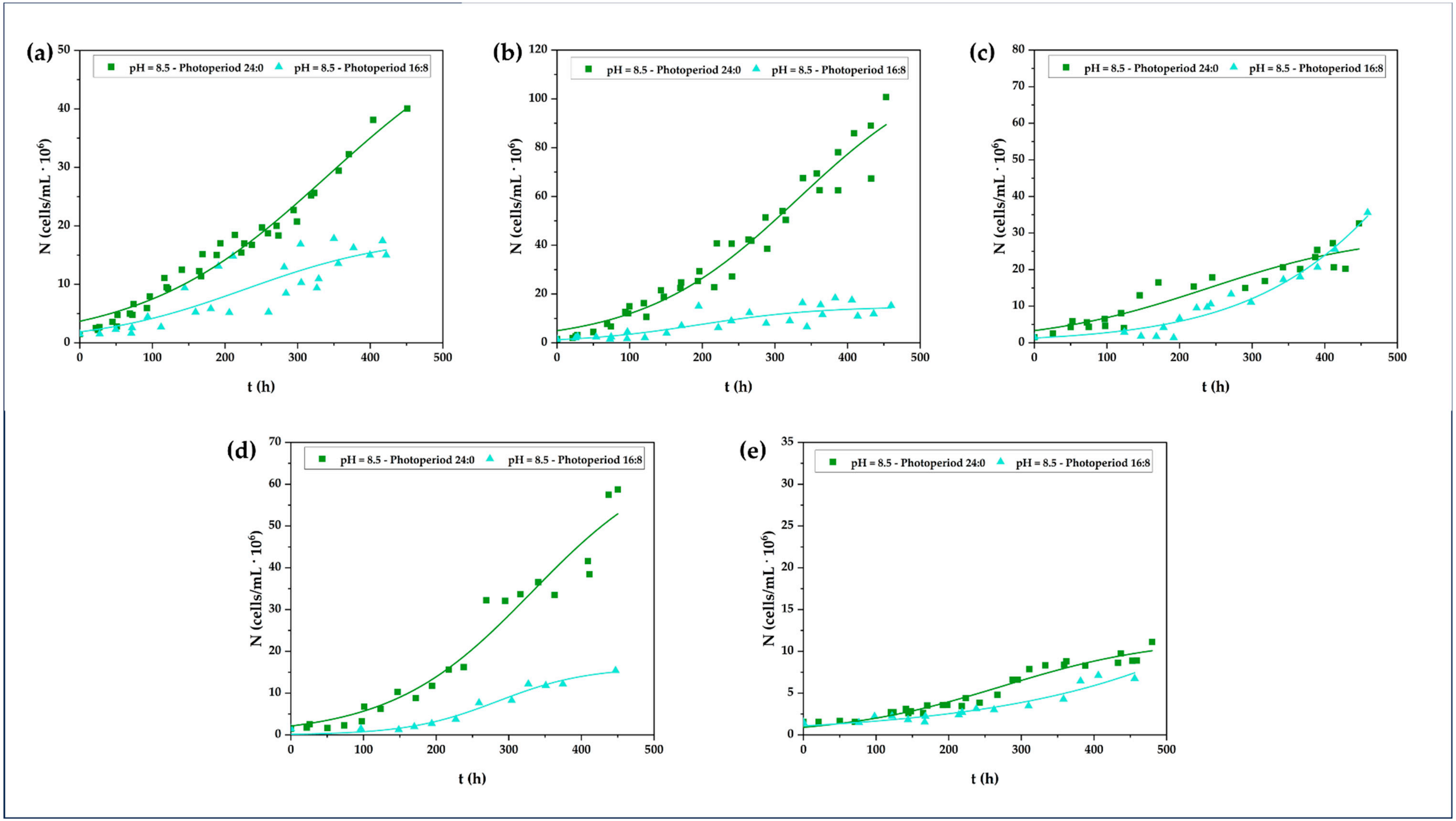

Figure S2. Growth curves of (a) *Picochlorum costavermella* VAS2.5, (b) *Picochlorum oklahomense* PAT3.2B, (c) *Picochlorum oklahomense* SAG4.4, (d) *Microchloropsis gaditana* VON5.3, and (e) *Nephroselmis pyriformis* PAT2.7 cultured in modified Artificial Seawater (mASW), at pH = 8.5 and under applied photoperiod of 24:0 (h light:dark) or 16:8, in an Open-Pond Simulating Reactor (OPSR) of 8.7 L (V_w = 5 L) capacity (two biological replicates). Each point is an average of two measurements and the curves were obtained by fitting the Verhulst model to the experimental data. In the legend of each graph the different culture conditions are denoted.

Table S1. (a) Biomass production, reserve materials accumulation and growth parameters and (b) fatty acid composition of total lipids (TL) and their lipid fractions (neutral – N, glycolipids - G and phospholipids - P), of *Picochlorum costavermella* VAS2.5 grown in modified Artificial Seawater (mASW) under photoperiod of 24:0 (h light:dark) at pH = 8.5 (Control) or pH = 7.5 in an 8.7 L (V_w = 5 L) capacity Open-Pond Simulating Reactor (OPSR).

a

Growth medium	t (h)	Biomass (x)	Lipids (L)				Polysaccharides (S)	Proteins (P)	Pigments		Growth parameters	
		x (mg/L)	L/x (%)	Lipid fractions (%) N	G+S	P	S/x (%)	P/x (%)	TCh/x (%)	TC/x (%)	μ (1/d)	R^2
**pH = 8.5 (Control)**	240	100.7 ± 12.4	22.9 ± 1.9	UND	UND	UND	12.1 ± 0.1	45.1 ± 6.4	0.4 ± 0.0	0.2 ± 0.0	0.19 ± 0.00	0.97
	450	269.1 ± 6.7	16.1 ± 0.0	16.6 ± 0.3	54.9 ± 6.2	28.5 ± 6.5	15.2 ± 0.3	51.0 ± 2.5	5.1 ± 0.2	2.4 ± 0.8		
**pH = 7.5**	240	66.8 ± 3.2	26.5 ± 12.1	UND	UND	UND	10.4 ± 2.1	43.0 ± 10.8	13.3 ± 1.9	5.3 ± 0.7	0.21 ± 0.05	0.87
	450	144.3 ± 57.5	34.4 ± 4.0	41.3 ± 3.8	42.2 ± 0.1	15.6 ± 4.5	9.4 ± 0.4	36.7 ± 3.9	8.2 ± 0.7	1.6 ± 0.7		

b

Growth medium	t (h)	Lipid fraction	Fatty acid composition of total lipids and their fractions (%, wt/wt) 14:0	14:1(n-5)	16:0	16:1(n-7)	17:0	18:0	18:1(n-9)	18:2(n-6)	18:3(n-3)	20:1(n-9)	20:5(n-3)	Others
**pH = 8.5 (Control)**	240	TL	5.1 ± 1.1	3.2 ± 0.1	16.5 ± 0.8	24.0 ± 4.0	<0.1	1.5 ± 0.0	25.2 ± 5.6	4.8 ± 1.8	<0.5	2.5 ± 0.6	16.4 ± 3.2	1.7 ± 0.3
	450	TL	4.0 ± 1.4	3.3 ± 1.0	17.2 ± 2.4	20.0 ± 4.6	<0.1	1.5 ± 0.4	30.8 ± 11.5	6.5 ± 2.6	<0.5	1.9 ± 0.3	13.2 ± 4.4	1.5 ± 0.5
		N	3.8 ± 1.5	3.8 ± 1.7	25.0 ± 1.5	26.4 ± 6.5	<0.5	1.9 ± 0.7	26.3 ± 12.6	2.6 ± 1.5	0.7 ± 0.0	3.5 ± 2.7	3.6 ± 1.1	2.1 ± 0.9
		G	9.0 ± 0.5	4.7 ± 1.8	19.1 ± 2.3	29.1 ± 2.3	<0.5	1.1 ± 0.8	6.6 ± 0.1	1.8 ± 0.3	<0.5	1.9 ± 0.1	21.8 ± 3.8	2.4 ± 0.6
		P	1.3 ± 0.3	1.1 ± 0.6	16.4 ± 1.4	18.0 ± 2.9	<0.5	1.3 ± 0.2	39.4 ± 10.1	7.0 ± 0.6	0.7 ± 0.4	4.5 ± 2.9	9.4 ± 2.6	1.0 ± 0.1
**pH = 7.5**	240	TL	8.3 ± 0.6	4.2 ± 0.4	25.7 ± 0.7	34.0 ± 0.6	<0.5	0.6 ± 0.1	5.7 ± 0.4	1.9 ± 0.0	0.6 ± 0.0	1.5 ± 0.3	15.2 ± 0.1	2.3 ± 0.0
	450	TL	9.7 ± 1.0	3.4 ± 0.3	14.2 ± 0.6	24.2 ± 1.4	<0.5	1.5 ± 0.4	6.7 ± 1.5	4.0 ± 0.2	2.6 ± 0.7	2.8 ± 0.0	23.9 ± 0.3	6.8 ± 1.8
		N	5.2 ± 0.1	7.5 ± 0.0	23.8 ± 1.2	33.8 ± 0.8	<0.5	7.9 ± 0.1	8.6 ± 0.5	1.8 ± 0.0	ND	1.9 ± 0.9	3.8 ± 1.1	5.2 ± 0.5
		G	11.6 ± 2.1	5.7 ± 1.1	21.6 ± 5.7	31.2 ± 1.5	<0.5	1.0 ± 0.5	3.6 ± 0.2	1.4 ± 0.1	ND	1.9 ± 0.2	19.4 ± 4.4	2.4 ± 0.4
		P	3.0 ± 0.2	0.6 ± 0.1	18.6 ± 4.4	31.5 ± 4.0	0.6 ± 0.1	1.0 ± 0.1	19.4 ± 1.6	6.6 ± 1.5	0.5 ± 0.2	3.2 ± 0.0	13.6 ± 1.1	1.6 ± 0.5

Abbreviations: (a) x (mg/L), dry biomass; L/x (%), lipids on dry biomass; N (%); neutral lipid fraction of total lipids; G+S (%); glycolipid and sphingolipid fraction of total lipids; P (%), fraction of phospholipids on total lipids; S/x (%), intracellular polysaccharides on dry biomass; P/x (%), intracellular proteins on dry biomass; TCh/x (%), total chlorophyll (chlorophyll a and b) on dry biomass; TC/x (%), total carotenoids on dry biomass; μ (1/d), maximum specific growth rate; R^2, the R-squared statistical measure; UND, undetermined. (b) ND: not detected, *Others: mainly 10:0, 12:0 an in some cases 18:3(n-6) Note: only glycolipids (i.e., G fraction) are mentioned as the amide bond of sphingolipids resists methanolysis during methyl esterification.

Table S2. (a) Biomass production, reserve materials accumulation and growth parameters and (b) fatty acid composition of total lipids (TL) and their lipid fractions (neutral – N, glycolipids - G and phospholipids - P), of *Picochlorum costavermella* VAS2.5 grown in modified Artificial Seawater (mASW) at pH = 8.5 under photoperiod of 24:0 (h light:dark) or photoperiod 16:8 in an 8.7 L (V_w = 5 L) capacity Open-Pond Simulating Reactor (OPSR).

a

Growth medium	t (h)	Biomass (x)	Lipids (L)				Polysaccharides (S)	Proteins (P)	Pigments		Growth parameters	
		x (mg/L)	L/x (%)	Lipid fractions (%) N	G+S	P	S/x (%)	P/x (%)	TCh/x (%)	TC/x (%)	μ (1/d)	R^2
**Photoperiod 24:0 (Control)**	240	100.7 ± 12.4	22.9 ± 1.9	UND	UND	UND	12.1 ± 0.1	45.1 ± 6.4	0.4 ± 0.0	0.2 ± 0.0	0.19 ± 0.00	0.97
	450	269.1 ± 6.7	16.1 ± 0.0	16.6 ± 0.3	54.9 ± 6.2	28.5 ± 6.5	15.2 ± 0.3	51.0 ± 2.5	5.1 ± 0.2	2.4 ± 0.8		
**Photoperiod 16:8**	240	129.7 ± 59.8	7.4 ± 0.2	UND	UND	UND	8.0 ± 0.3	9.5 ± 0.1	3.7 ± 1.1	0.8 ± 0.3	0.23 ± 0.09	0.73
	450	220.7 ± 47.6	7.3 ± 1.0	28.6 ± 5.8	60.7 ± 11.2	10.3 ± 5.7	10.0 ± 0.9	32.6 ± 4.3	2.8 ± 1.3	1.0 ± 0.5		

b

Growth medium	t (h)	Lipid fraction	Fatty acid composition of total lipids and their fractions (%, wt/wt) 14:0	14:1(n-5)	16:0	16:1(n-7)	17:0	18:0	18:1(n-9)	18:2(n-6)	18:3(n-3)	20:1(n-9)	20:5(n-3)	*Others
**Photoperiod 24:0 (Control)**	240	TL	5.1 ± 1.1	3.2 ± 0.1	16.5 ± 0.8	24.0 ± 4.0	<0.1	1.5 ± 0.0	25.2 ± 5.6	4.8 ± 1.8	<0.5	2.5 ± 0.6	16.4 ± 3.2	1.7 ± 0.3
	450	TL	4.0 ± 1.4	3.3 ± 1.0	17.2 ± 2.4	20.0 ± 4.6	<0.1	1.5 ± 0.4	30.8 ± 11.5	6.5 ± 2.6	<0.5	1.9 ± 0.3	13.2 ± 4.4	1.5 ± 0.5
		N	3.8 ± 1.5	3.8 ± 1.7	25.0 ± 1.5	26.4 ± 6.5	<0.5	1.9 ± 0.7	26.3 ± 12.6	2.6 ± 1.5	0.7 ± 0.0	3.5 ± 2.7	3.6 ± 1.1	2.1 ± 0.9
		G	9.0 ± 0.5	4.7 ± 1.8	19.1 ± 2.3	29.1 ± 2.3	<0.5	1.1 ± 0.8	6.6 ± 0.1	1.8 ± 0.3	<0.5	1.9 ± 0.1	21.8 ± 3.8	2.4 ± 0.6
		P	1.3 ± 0.3	1.1 ± 0.6	16.4 ± 1.4	18.0 ± 2.9	<0.5	1.3 ± 0.2	39.4 ± 10.1	7.0 ± 0.6	0.7 ± 0.4	4.5 ± 2.9	9.4 ± 2.6	1.0 ± 0.1
**Photoperiod 16:8**	240	TL	6.0 ± 1.6	1.9 ± 0.2	26.4 ± 1.8	27.5 ± 3.5	ND	2.9 ± 0.4	11.0 ± 0.6	2.8 ± 1.6	1.3 ± 0.0	7.5 ± 2.2	9.4 ± 0.6	3.8 ± 2.3
	450	TL	5.2 ± 0.8	2.8 ± 0.9	23.8 ± 2.6	26.4 ± 1.1	ND	4.5 ± 1.6	13.9 ± 2.9	2.9 ± 0.1	2.0 ± 0.7	6.5 ± 1.0	8.0 ± 1.0	4.2 ± 0.4
		N	4.4 ± 1.1	3.7 ± 0.1	21.2 ±0.2	25.3 ± 0.7	0.7 ± 0.4	6.4 ± 0.3	17.1 ± 0.4	3.0 ± 0.4	<0.5	4.8 ± 0.7	6.6 ± 0.4	3.3 ± 0.2
		G	10.6 ± 0.2	10.1 ± 0.0	10.9 ± 0.0	17.4 ± 0.1	0.5 ± 0.1	1.3 ± 0.0	8.4 ± 1.2	2.2 ± 0.2	<0.5	2.7 ± 0.0	31.4 ± 1.7	5.9 ± 1.8
		P	1.9 ± 1.3	2.1 ± 0.0	12.3 ± 2.0	22.4 ± 1.0	2.1 ± 0.0	6.2 ± 5.0	7.5 ± 0.3	1.9 ± 0.5	1.6 ± 0.5	3.0 ± 0.1	36.9 ± 0.3	3.3 ± 0.3

Abbreviations: (a) x (mg/L), dry biomass; L/x (%), lipids on dry biomass; N (%); neutral lipid fraction of total lipids; G+S (%); glycolipid and sphingolipid fraction of total lipids; P (%), fraction of phospholipids on total lipids; S/x (%), intracellular polysaccharides on dry biomass; P/x (%), intracellular proteins on dry biomass; TCh/x (%), total chlorophyll (chlorophyll a and b) on dry biomass; TC/x (%), total carotenoids on dry biomass; μ (1/d), maximum specific growth rate; R^2, the R-squared statistical measure; UND, undetermined. (b) ND: not detected, *Others: mainly 10:0, 12:0 an in some cases C18:3(n-6), Note: only glycolipids (i.e., G fraction) are mentioned as the amide bond of sphingolipids resists methanolysis during methyl esterification.

Table S3. (a) Biomass production, reserve materials accumulation and growth parameters and (b) fatty acid composition of total lipids (TL) and their lipid fractions (neutral – N, glycolipids - G and phospholipids - P), of *Picochlorum oklahomense* PAT3.2B grown in modified Artificial Seawater (mASW) under photoperiod of 24:0 (h light:dark) at pH = 8.5 (Control) or pH = 7.5 in an 8.7 L (Vw = 5 L) capacity Open-Pond Simulating Reactor (OPSR).

a

Growth medium	t (h)	Biomass (x)	Lipids (L)				Polysaccharides (S)	Proteins (P)	Pigments		Growth parameters	
		x (mg/L)	L/x (%)	Lipid fractions (%)			S/x (%)	P/x (%)	TCh/x (%)	TC/x (%)	μ (1/d)	R^2
				N	G+S	P						
**pH = 8.5 (Control)**	240	100.0 ± 9.1	13.9 ± 1.5	UND	UND	UND	13.1 ± 0.6	58.4 ± 0.9	25.2 ± 1.7	4.7 ± 0.5	0.23 ± 0.03	0.96
	450	421.1 ± 30.8	11.5 ± 0.3	34.9 ± 2.3	54.4 ± 3.9	12.9 ± 1.6	8.8 ± 0.6	49.2 ± 4.8	9.0 ± 2.8	1.5 ± 0.6		
**pH = 7.5**	240	49.0 ± 6.1	24.2 ± 11.0	UND	UND	UND	12.0 ± 5.4	43.0 ± 10.8	23.6 ± 3.3	5.0 ± 0.1	0.17 ± 0.06	0.81
	450	241.1 ± 62.5	7.8 ± 1.8	UND	UND	UND	19.8 ± 4.1	51.9 ± 8.5	8.0 ± 2.9	1.7 ± 0.7		

b

Growth medium	t (h)	Lipid fraction	Fatty acid composition of total lipids and their fractions (%, wt/wt)										
			**14:0**	**14:1(n-5)**	**16:0**	**16:1(n-7)**	**17:0**	**18:0**	**18:1(n-9)**	**18:2(n-6)**	**18:3(n-3)**	**18:4(n-3)**	**Others**
**pH = 8.5 (Control)**	240	TL	4.8 ± 0.7	2.7 ± 0.5	14.3 ± 0.9	2.3 ± 0.6	2.9 ± 0.7	7.5 ± 0.3	20.9 ± 2.4	4.8 ± 0.1	22.1 ± 2.4	4.5 ± 4.0	13.4 ± 4.1
	450	TL	1.7 ± 0.2	6.9 ± 0.6	17.1 ± 1.8	3.0 ± 0.3	<0.5	7.3 ± 1.4	14.7 ± 0.1	21.4 ± 0.8	23.0 ± 0.6	0.5 ± 0.0	4.4 ± 0.3
		N	5.6 ± 0.3	26.7 ± 2.9	10.6 ± 1.2	2.1 ± 0.3	1.6 ± 0.3	3.1 ± 0.2	9.2 ± 2.4	18.0 ± 2.0	9.5 ± 0.2	1.4 ± 0.5	16.7 ± 4.4
		G	<0.5	<0.5	18.6 ± 0.2	2.1 ± 0.1	ND	13.6 ± 0.0	14.4 ± 0.6	27.5 ± 0.0	20.3 ± 0.3	3.3 ± 0.5	<0.5
		P	1.0 ± 0.3	11.4 ± 2.6	11.8 ± 3.6	3.3 ± 1.0	1.3 ± 0.1	5.8 ± 2.4	12.5 ± 0.8	37.5 ± 2.0	17.7 ± 1.1	1.9 ± 0.8	0.9 ± 0.1
**pH = 7.5**	240	TL	2.1 ± 0.5	6.7 ± 0.8	18.3 ± 1.0	2.8 ± 0.5	4.6 ± 1.3	1.0 ± 0.2	14.4 ± 1.2	21.4 ± 2.5	20.9 ± 5.4	4.3 ± 1.5	3.6 ± 0.2
	450	TL	2.0 ± 0.6	11.4 ± 2.6	11.8 ± 3.6	3.3 ± 1.0	ND	7.0 ± 2.2	13.3 ± 1.4	20.8 ± 1.6	22.8 ± 4.5	1.5 ± 0.5	3.3 ± 1.8

Abbreviations: (a) x (mg/L), dry biomass; L/x (%), lipids on dry biomass; N (%); neutral lipid fraction of total lipids; G+S (%); glycolipid and sphingolipid fraction of total lipids; P (%), fraction of phospholipids on total lipids; S/x (%), intracellular polysaccharides on dry biomass; P/x (%), intracellular proteins on dry biomass; TCh/x (%), total chlorophyll (chlorophyll a and b) on dry biomass; TC/x (%), total carotenoids on dry biomass; μ (1/d), maximum specific growth rate; R^2, the R-squared statistical measure; UND, undetermined. (b) ND: not detected, *Others: mainly 10:0, 12:0 an in some cases 18:3(n-6)

Note: only glycolipids (i.e., G fraction) are mentioned as the amide bond of sphingolipids resists methanolysis during methyl esterification.

Table S4. (a) Biomass production, reserve materials accumulation and growth parameters and (b) fatty acid composition of total lipids (TL) and their lipid fractions (neutral – N, glycolipids - G and phospholipids - P), of *Picochlorum oklahomense* PAT3.2B grown in modified Artificial Seawater (mASW) at pH = 8.5 under photoperiod of 24:0 (h light:dark) or photoperiod 16:8 in an 8.7 L (Vw = 5 L) capacity Open-Pond Simulating Reactor (OPSR).

a

Growth medium	t (h)	Biomass (x)	Lipids (L)				Polysaccharides (S)	Proteins (P)	Pigments		Growth parameters	
		x (mg/L)	L/x (%)	Lipid fractions (%)			S/x (%)	P/x (%)	TCh/x (%)	TC/x (%)	μ (1/d)	R^2
				N	G+S	P						
Photoperiod 24:0 (Control)	240	100.0 ± 9.1	13.9 ± 1.5	UND	UND	UND	13.1 ± 0.6	58.4 ± 0.9	25.2 ± 1.7	4.7 ± 0.5	0.23 ± 0.03	0.96
	450	421.1 ± 30.8	11.5 ± 0.3	34.9 ± 2.3	54.4 ± 3.9	12.9 ± 1.6	8.8 ± 0.6	49.2 ± 4.8	9.0 ± 2.8	1.5 ± 0.6		
Photoperiod 16:8	240	47.0 ± 7.3	3.2 ± 0.5	UND	UND	UND	5.8 ± 0.9	6.8 ± 1.1	UND	UND	0.29 ± 0.11	0.73
	450	154.6 ± 36.6	1.0 ± 0.4	UND	UND	UND	5.6 ± 0.1	21.1 ± 2.8	2.7 ± 0.0	0.5 ± 0.1		

b

Growth medium	t (h)	Lipid fraction	Fatty acid composition of total lipids and their fractions (%, wt/wt)										
			14:0	14:1(n-5)	16:0	16:1(n-7)	17:0	18:0	18:1(n-9)	18:2(n-6)	18:3(n-3)	18:4(n-3)	Others
Photoperiod 24:0 (Control)	240	TL	4.8 ± 0.7	2.7 ± 0.5	14.3 ± 0.9	2.3 ± 0.6	2.9 ± 0.7	7.5 ± 0.3	20.9 ± 2.4	4.8 ± 0.1	22.1 ± 2.4	4.5 ± 4.0	13.4 ± 4.1
	450	TL	1.7 ± 0.2	6.9 ± 0.6	17.1 ± 1.8	3.0 ± 0.3	<0.5	7.3 ± 1.4	14.7 ± 0.1	21.4 ± 0.8	23.0 ± 0.6	0.5 ± 0.0	4.4 ± 0.3
		N	5.6 ± 0.3	26.7 ± 2.9	10.6 ± 1.2	2.1 ± 0.3	1.6 ± 0.3	3.1 ± 0.2	9.2 ± 2.4	18.0 ± 2.0	9.5 ± 0.2	1.4 ± 0.5	16.7 ± 4.4
		G	<0.5	<0.5	18.6 ± 0.2	2.1 ± 0.1	ND	13.6 ± 0.0	14.4 ± 0.6	27.5 ± 0.0	20.3 ± 0.3	3.3 ± 0.5	<0.5
		P	1.0 ± 0.3	11.4 ± 2.6	11.8 ± 3.6	3.3 ± 1.0	1.3 ± 0.1	5.8 ± 2.4	12.5 ± 0.8	37.5 ± 2.0	17.7 ± 1.1	1.9 ± 0.8	0.9 ± 0.1
Photoperiod 16:8	450	TL	4.5 ± 1.2	8.6 ± 0.6	13.7 ± 0.3	2.4 ± 0.0	ND	8.0 ± 0.8	14.2 ± 1.6	26.7 ± 6.3	18.5 ± 6.3	ND	3.3 ± 1.8

Abbreviations: (a) x (mg/L), dry biomass; L/x (%), lipids on dry biomass; N (%); neutral lipid fraction of total lipids; G+S (%); glycolipid and sphingolipid fraction of total lipids; P (%), fraction of phospholipids on total lipids; S/x (%), intracellular polysaccharides on dry biomass; P/x (%), intracellular proteins on dry biomass; TCh/x (%), total chlorophyll (chlorophyll a and b) on dry biomass; TC/x (%), total carotenoids on dry biomass; μ (1/d), maximum specific growth rate; R^2, the R-squared statistical measure; UND, undetermined. (b) ND: not detected, *Others: mainly 10:0, 12:0 an in some cases 18:3(n-6)

Note: only glycolipids (i.e., G fraction) are mentioned as the amide bond of sphingolipids resists methanolysis during methyl esterification.

Table S5. (a) Biomass production, reserve materials accumulation and growth parameters and (b) fatty acid composition of total lipids (TL) and their lipid fractions (neutral – N, glycolipids - G and phospholipids - P), of *Picochlorum oklahomense* SAG4.4 grown in modified Artificial Seawater (mASW) under photoperiod of 24:0 (h light:dark) at pH = 8.5 (Control) or pH = 7.5 in an 8.7 L (V_w = 5 L) capacity Open-Pond Simulating Reactor (OPSR).

a

Growth medium	t (h)	Biomass (x)	Lipids (L)				Polysaccharides (S)	Proteins (P)	Pigments		Growth parameters	
		x (mg/L)	L/x (%)	Lipid fractions (%)			S/x (%)	P/x (%)	TCh/x (%)	TC/x (%)	μ (1/d)	R^2
				N	G+S	P						
**pH = 8.5 (Control)**	240	90.7 ± 4.9	8.6 ± 2.0	UND	UND	UND	11.2 ± 0.8	35.0 ± 1.9	1.5 ± 0.1	0.3 ± 0.0	0.21 ± 0.06	0.87
	450	299.3 ± 13.6	9.4 ± 1.5	16.9 ± 0.9	68.7 ± 0.3	15.4 ± 1.1	13.1 ± 0.7	43.8 ± 2.3	4.7 ± 1.1	1.3 ± 0.0		
**pH = 7.5**	240	76.6 ± 0.4	14.6 ± 0.6	UND	UND	UND	13.2 ± 0.9	47.4 ± 1.7	UND	UND	0.16 ± 0.03	0.94
	450	216.2 ± 1.3	11.8 ± 0.1	UND	UND	UND	12.8 ± 0.0	40.7 ± 5.5	UND	UND		

b

Growth medium	t (h)	Lipid fraction	Fatty acid composition of total lipids and their fractions (%, wt/wt)													
			**14:0**	**14:1(n-5)**	**16:0**	**16:1(n-7)**	**17:0**	**18:0**	**18:1(n-9)**	**18:2(n-6)**	**18:3(n-6)**	**18:3(n-3)**	**18:4(n-3)**	**20:1(n-9)**	**20:5(n-3)**	**Others**
**pH = 8.5 (Control)**	240	TL	4.1 ± 1.9	2.2 ± 0.6	13.4 ± 0.1	1.7 ± 0.1	2.2 ± 0.6	4.7 ± 1.1	18.4 ± 0.7	4.7 ± 1.8	7.6 ± 1.0	19.7 ± 5.0	8.5 ± 2.4	ND	ND	12.8 ± 1.7
	450	TL	1.7 ± 0.2	6.9 ± 0.6	17.1 ± 1.8	3.0 ± 0.3	<0.5	7.3 ± 1.4	14.7 ± 0.1	21.4 ± 0.8	<0.5	23.0 ± 0.6	1.0 ± 0.0	ND	ND	4.4 ± 0.3
		N	16.4 ± 0.4	13.1 ± 0.4	14.1 ± 0.5	3.1 ± 0.1	2.1 ± 0.0	0.8 ± 0.0	11.5 ± 0.4	17.3 ± 0.8	4.3 ± 0.2	13.2 ± 1.0	0.6 ± 0.2	1.6 ± 0.3	ND	2.6 ± 0.1
		G	0.5 ± 0.1	0.6 ± 0.1	18.8 ± 0.6	2.4 ± 0.0	<0.5	11.0 ± 0.1	19.0 ± 0.5	21.0 ± 0.7	ND	24.4 ± 1.5	2.1 ± 0.0	ND	ND	<0.5
		P	0.6 ± 0.1	0.8 ± 0.2	26.5 ± 0.0	5.6 ± 0.5	0.9 ± 0.2	2.5 ± 0.3	15.9 ± 1.7	26.9 ± 0.6	ND	16.3 ± 1.1	2.8 ± 0.0	ND	ND	1.2 ± 0.5
**pH = 7.5**	240	TL	0.5 ± 0.2	1.3 ± 0.2	12.4 ± 1.1	12.9 ±0.6	1.5 ± 0.1	6.6 ± 0.2	51.0 ± 0.6	4.9 ± 0.1	ND	4.8 ± 0.8	ND	ND	ND	4.2 ± 0.2
	450	TL	1.7 ± 0.1	6.3 ± 0.3	21.4 ± 0.4	5.5 ± 0.0	5.0 ± 0.0	0.7 ± 0.0	14.3 ± 0.1	19.4 ± 0.8	ND	13.6 ± 0.2	0.6 ± 0.1	1.2 ± 0.1	2.8 ± 0.7	7.5 ± 0.1

Abbreviations: (a) x (mg/L), dry biomass; L/x (%), lipids on dry biomass; N (%); neutral lipid fraction of total lipids; G+S (%); glycolipid and sphingolipid fraction of total lipids; P (%), fraction of phospholipids on total lipids; S/x (%), intracellular polysaccharides on dry biomass; P/x (%), intracellular proteins on dry biomass; TCh/x (%), total chlorophyll (chlorophyll a and b) on dry biomass; TC/x (%), total carotenoids on dry biomass; μ (1/d), maximum specific growth rate; R^2, the R-squared statistical measure; UND, undetermined. (b) ND: not detected, *Others: mainly 10:0, 12:0. Note: only glycolipids (i.e., G fraction) are mentioned as the amide bond of sphingolipids resists methanolysis during methyl esterification.

Table S6. (a) Biomass production, reserve materials accumulation and growth parameters and (b) fatty acid composition of total lipids (TL) and their lipid fractions (neutral – N, glycolipids - G and phospholipids - P), of *Picochlorum oklahomense* SAG4.4 grown in modified Artificial Seawater (mASW) at pH = 8.5 under photoperiod of 24:0 (h light:dark) or photoperiod 16:8 in an 8.7 L (Vw = 5 L) capacity Open-Pond Simulating Reactor (OPSR).

a

Growth medium	t (h)	Biomass (x)	Lipids (L)				Polysaccharides (S)	Proteins (P)	Pigments		Growth parameters	
		x (mg/L)	L/x (%)	Lipid fractions (%) N	G+S	P	S/x (%)	P/x (%)	TCh/x (%)	TC/x (%)	μ (1/d)	R^2
**Photoperiod 24:0 (Control)**	240	90.7 ± 4.9	8.6 ± 2.0	UND	UND	UND	11.2 ± 0.8	35.0 ± 1.9	1.5 ± 0.1	0.3 ± 0.0	0.21 ± 0.06	0.87
	450	299.3 ± 13.6	9.4 ± 1.5	16.9 ± 0.9	68.7 ± 0.3	15.4 ± 1.1	13.1 ± 0.7	43.8 ± 2.3	4.7 ± 1.1	1.3 ± 0.0		
**Photoperiod 16:8**	240	62.4 ± 2.8	1.8 ± 0.8	UND	UND	UND	6.2 ± 0.1	9.6 ± 0.1	1.8 ± 0.1	0.5 ± 0.0	0.18 ± 0.04	0.95
	450	199.7 ± 0.0	1.4 ± 0.3	UND	UND	UND	7.9 ± 0.7	23.5 ± 0.5	1.9 ± 0.0	0.4 ± 0.0		

b

Growth medium	t (h)	Lipid fraction	14:0	14:1(n-5)	16:0	16:1(n-7)	17:0	18:0	18:1(n-9)	18:2(n-6)	18:3(n-6)	18:3(n-3)	18:4(n-3)	20:1(n-9)	20:5(n-3)	Others
			**Fatty acid composition of total lipids and their fractions (%, wt/wt)**													
**Photoperiod 24:0 (Control)**	240	TL	4.1 ± 1.9	2.2 ± 0.6	13.4 ± 0.1	1.7 ± 0.1	2.2 ± 0.6	4.7 ± 1.1	18.4 ± 0.7	4.7 ± 1.8	7.6 ± 1.0	19.7 ± 5.0	8.5 ± 2.4	ND	ND	12.8 ± 1.7
	450	TL	1.7 ± 0.2	6.9 ± 0.6	17.1 ± 1.8	3.0 ± 0.3	<0.5	7.3 ± 1.4	14.7 ± 0.1	21.4 ± 0.8	<0.5	23.0 ± 0.6	1.0 ± 0.0	ND	ND	4.4 ± 0.3
		N	16.4 ± 0.4	13.1 ± 0.4	14.1 ± 0.5	3.1 ± 0.1	2.1 ± 0.0	0.8 ± 0.0	11.5 ± 0.4	17.3 ± 0.8	4.3 ± 0.2	13.2 ± 1.0	0.6 ± 0.2	1.6 ± 0.3	ND	2.6 ± 0.1
		G	0.5 ± 0.1	0.6 ± 0.1	18.8 ± 0.6	2.4 ± 0.0	<0.5	11.0 ± 0.1	19.0 ± 0.5	21.0 ± 0.7	ND	24.4 ± 1.5	2.1 ± 0.0	ND	ND	<0.5
		P	0.6 ± 0.1	0.8 ± 0.2	26.5 ± 0.0	5.6 ± 0.5	0.9 ± 0.2	2.5 ± 0.3	15.9 ± 1.7	26.9 ± 0.6	ND	16.3 ± 1.1	2.8 ± 0.0	ND	ND	1.2 ± 0.5
**Photoperiod 16:8**	240	TL	6.3 ± 0.1	4.0 ± 0.5	6.7 ± 0.4	6.4 ± 3.0	11.6 ± 2.9	10.0 ± 4.7	19.1 ± 5.6	11.0 ± 4.0	ND	8.9 ± 2.1	ND	ND	ND	16.1 ± 0.6
	450	TL	5.2 ± 1.1	5.5 ± 1.5	6.9 ± 0.2	7.9 ± 1.5	11.6 ± 0.0	10.4 ± 0.4	21.1 ± 2.0	10.0 ± 1.0	ND	9.1 ± 0.1	ND	ND	ND	12.5 ± 3.6

Abbreviations: (a) x (mg/L), dry biomass; L/x (%), lipids on dry biomass; N (%); neutral lipid fraction of total lipids; G+S (%); glycolipid and sphingolipid fraction of total lipids; P (%), fraction of phospholipids on total lipids; S/x (%), intracellular polysaccharides on dry biomass; P/x (%), intracellular proteins on dry biomass; TCh/x (%), total chlorophyll (chlorophyll a and b) on dry biomass; TC/x (%), total carotenoids on dry biomass; μ (1/d), maximum specific growth rate; R^2, the R-squared statistical measure; UND, undetermined. (b) ND: not detected, *Others: mainly 10:0, 12:0. Note: only glycolipids (i.e., G fraction) are mentioned as the amide bond of sphingolipids resists methanolysis during methyl esterification.

Table S7. (a) Biomass production, reserve materials accumulation and growth parameters and (b) fatty acid composition of total lipids (TL) and their lipid fractions (neutral – N, glycolipids - G and phospholipids - P), of *Microchloropsis gaditana* VON5.3 grown in modified Artificial Seawater (mASW) under photoperiod of 24:0 (h light:dark) at pH = 8.5 (Control) or pH = 7.5 in an 8.7 L (V_w = 5 L) capacity Open-Pond Simulating Reactor (OPSR).

a

Growth medium	t (h)	Biomass (x)	Lipids (L)				Polysaccharides (S)	Proteins (P)	Pigments		Growth parameters	
		x (mg/L)	L/x (%)	Lipid fractions (%)			S/x (%)	P/x (%)	TCh/x (%)	TC/x (%)	μ (1/d)	R^2
				N	G+S	P						
**pH = 8.5 (Control)**	240	109.3 ± 36.5	10.7 ± 2.7	UND	UND	UND	7.0 ± 0.4	18.7 ± 0.5	3.2 ± 0.8	1.5 ± 0.2	0.25 ± 0.05	0.95
	450	523.0 ± 136.0	11.8 ± 3.5	34.4 ± 9.1	56.9 ± 5.7	5.4 ± 0.1	9.7 ± 0.4	21.9 ± 4.9	0.4 ± 0.1	0.6 ± 0.4		
**pH = 7.5**	240	68.0 ± 16.5	12.0 ± 3.3	UND	UND	UND	8.7 ± 2.1	46.9 ± 16.1	6.2 ± 1.5	2.5 ± 0.6	0.39 ± 0.07	0.92
	450	439.6 ± 47.2	14.1 ± 0.8	17.7 ± 1.4	59.3 ± 0.6	23.1 ± 2.0	10.5 ± 1.1	32.1 ± 5.0	2.9 ± 0.3	1.3 ± 0.1		

b

Growth medium	t (h)	Lipid fraction	Fatty acid composition of total lipids and their fractions (%, wt/wt)												
			14:0	14:1(n-5)	16:0	16:1(n-7)	17:0	18:0	18:1(n-9)	18:2(n-6)	18:3(n-3)	18:4(n-3)	20:1(n-9)	20:5(n-3)	Others
**pH = 8.5 (Control)**	240	TL	6.9 ± 1.2	1.9 ± 1.0	23.2 ± 0.0	28.5 ± 3.6	<0.5	0.8 ± 0.4	7.5 ± 0.2	1.9 ± 0.6	1.8 ± 0.8	0.1 ± 0.0	3.5 ± 1.0	17.6 ± 1.4	6.2 ± 3.4
	450	TL	6.6 ± 0.9	3.8 ± 0.9	22.8 ± 0.4	31.3 ± 0.8	0.3 ± 0.0	0.4 ± 0.1	6.8 ± 0.9	2.0 ± 0.5	0.7 ± 0.2	0.1 ± 0.0	2.9 ± 0.4	20.0 ± 1.0	2.5 ± 0.3
		N	5.7 ± 1.4	3.0 ± 1.2	28.6 ± 0.4	34.9 ± 2.7	0.5 ± 0.3	0.6 ± 0.1	8.1 ± 1.2	1.4 ± 0.1	ND	1.8 ± 0.6	2.5 ± 0.6	8.5 ± 1.0	4.9 ± 0.3
		G	9.1 ± 0.0	5.6 ± 0.8	20.8 ± 2.0	28.1 ± 2.8	<0.5	ND	6.4 ± 1.1	1.3 ± 0.4	ND	ND	2.0 ± 0.7	22.3 ± 3.0	4.1 ± 0.5
		P	2.4 ± 0.1	0.6 ± 0.0	21.9 ± 0.2	31.8 ± 0.8	0.8 ± 0.3	<0.5	16.3 ± 1.5	4.4 ± 0.1	0.6 ± 0.2	ND	3.5 ± 0.9	15.7 ± 0.7	2.0 ± 0.4
**pH = 7.5**	240	TL	7.2 ± 0.3	3.5 ± 0.3	21.2 ± 1.6	26.7 ± 1.8	ND	ND	7.7 ± 0.2	2.8 ± 0.3	2.7 ± 0.1	ND	ND	21.4 ± 1.4	6.8 ± 1.4
	450	TL	7.5 ± 0.0	4.1 ± 1.0	18.9 ± 0.8	27.2 ± 2.3	0.3 ± 0.0	0.3 ± 0.1	6.3 ± 1.7	2.5 ± 0.6	1.6 ± 1.2	0.3 ± 0.2	3.3 ± 0.4	24.4 ± 1.5	3.6 ± 1.0
		N	5.7 ± 0.0	7.2 ± 3.0	24.8 ± 3.8	34.1 ± 0.8	0.4 ± 0.1	0.7 ± 0.1	5.8 ± 1.1	1.4 ± 0.0	<0.1	2.1 ± 0.3	2.2 ± 0.3	10.0 ± 0.5	5.7 ± 0.8
		G	10.9 ± 0.8	4.5 ± 0.2	22.1 ± 0.7	28.4 ± 1.6	<0.1	<0.1	4.4 ± 0.1	2.1 ± 0.2	ND	ND	2.1 ± 0.1	23.2 ± 0.6	2.4 ± 0.6
		P	2.7 ± 0.0	0.8 ± 0.2	19.0 ± 0.8	28.0 ± 2.6	<0.1	<0.1	13.2 ± 0.8	5.1 ± 0.6	<0.1	ND	4.5 ± 1.0	23.3 ± 1.2	3.4 ± 0.4

Abbreviations: (a) x (mg/L), dry biomass; L/x (%), lipids on dry biomass; N (%); neutral lipid fraction of total lipids; G+S (%); glycolipid and sphingolipid fraction of total lipids; P (%), fraction of phospholipids on total lipids; S/x (%), intracellular polysaccharides on dry biomass; P/x (%), intracellular proteins on dry biomass; TCh/x (%), total chlorophyll (chlorophyll a and b) on dry biomass; TC/x (%), total carotenoids on dry biomass; μ (1/d), maximum specific growth rate; R^2, the R-squared statistical measure; UND, undetermined. (b) ND: not detected, *Others: mainly 10:0, 12:0 an in some cases 18:3(n-6) Note: only glycolipids (i.e., G fraction) are mentioned as the amide bond of sphingolipids resists methanolysis during methyl esterification.

Table S8. (a) Biomass production, reserve materials accumulation and growth parameters and (b) fatty acid composition of total lipids (TL) and their lipid fractions (neutral – N, glycolipids - G and phospholipids - P), of *Microchloropsis gaditana* VON5.3 grown in modified Artificial Seawater (mASW) at pH = 8.5 under photoperiod of 24:0 (h light:dark) or photoperiod 16:8 in an 8.7 L (Vw = 5 L) capacity Open-Pond Simulating Reactor (OPSR).

a

Growth medium	t (h)	Biomass (x)	Lipids (L)				Polysaccharides (S)	Proteins (P)	Pigments		Growth parameters	
		x (mg/L)	L/x (%)	Lipid fractions (%)			S/x (%)	P/x (%)	TCh/x (%)	TC/x (%)	μ (1/d)	R^2
				N	G+S	P						
**Photoperiod 24:0 (Control)**	240	109.3 ± 36.5	10.7 ± 2.7	UND	UND	UND	7.0 ± 0.4	18.7 ± 0.5	3.2 ± 0.8	1.5 ± 0.2	0.25 ± 0.05	0.95
	450	523.0 ± 136.0	11.8 ± 3.5	34.4 ± 9.1	56.9 ± 5.7	5.4 ± 0.1	9.7 ± 0.4	21.9 ± 4.9	0.4 ± 0.1	0.6 ± 0.4		
**Photoperiod 16:8**	240	106.3 ± 7.3	4.4 ± 0.2	UND	UND	UND	4.7 ± 0.4	9.1 ± 0.2	1.7 ± 0.1	0.7 ± 0.0	0.39 ± 0.07	0.97
	450	177.0 ± 12.0	11.9 ± 0.8	UND	UND	UND	7.1 ± 0.8	23.6 ± 5.7	1.1 ± 0.1	0.8 ± 0.0		

b

Growth medium	t (h)	Lipid fraction	Fatty acid composition of total lipids and their fractions (%, wt/wt)												
			14:0	14:1(n-5)	16:0	16:1(n-7)	17:0	18:0	18:1(n-9)	18:2(n-6)	18:3(n-3)	18:4(n-3)	20:1(n-9)	20:5(n-3)	Others
**Photoperiod 24:0 (Control)**	240	TL	6.9 ± 1.2	1.9 ± 1.0	23.2 ± 0.0	28.5 ± 3.6	<0.5	0.8 ± 0.4	7.5 ± 0.2	1.9 ± 0.6	1.8 ± 0.8	≤0.1	3.5 ± 1.0	17.6 ± 1.4	6.2 ± 3.4
	450	TL	6.6 ± 0.9	3.8 ± 0.9	22.8 ± 0.4	31.3 ± 0.8	<0.5	≤0.5	6.8 ± 0.9	2.0 ± 0.5	0.7 ± 0.2	≤0.1	2.9 ± 0.4	20.0 ± 1.0	2.5 ± 0.3
		N	5.7 ± 1.4	3.0 ± 1.2	28.6 ± 0.4	34.9 ± 2.7	0.5 ± 0.3	0.6 ± 0.1	8.1 ± 1.2	1.4 ± 0.1	ND	1.8 ± 0.6	2.5 ± 0.6	8.5 ± 1.0	4.9 ± 0.3
		G	9.1 ± 0.0	5.6 ± 0.8	20.8 ± 2.0	28.1 ± 2.8	<0.5	ND	6.4 ± 1.1	1.3 ± 0.4	ND	ND	2.0 ± 0.7	22.3 ± 3.0	4.1 ± 0.5
		P	2.4 ± 0.1	0.6 ± 0.0	21.9 ± 0.2	31.8 ± 0.8	0.8 ± 0.3	<0.5	16.3 ± 1.5	4.4 ± 0.1	0.6 ± 0.2	ND	3.5 ± 0.9	15.7 ± 0.7	2.0 ± 0.4
**Photoperiod 16:8**	240	TL	3.2 ± 0.1	2.3 ± 0.1	22.1 ± 0.4	12.6 ± 0.0	ND	3.9 ± 0.6	36.2 ± 0.9	9.9 ± 1.0	2.0 ± 0.0	ND	1.4 ± 0.0	6.1 ± 0.5	2.9 ± 0.2
	450	TL	11.8 ± 1.0	7.5 ± 0.7	19.9 ± 1.0	25.9 ± 1.3	0.6 ± 0.1	1.2 ± 0.2	5.7 ± 0.6	2.8 ± 0.4	ND	2.7 ± 0.7	2.3 ± 0.1	16.1 ± 0.1	3.4 ± 0.4

Abbreviations: (a) x (mg/L), dry biomass; L/x (%), lipids on dry biomass; N (%); neutral lipid fraction of total lipids; G+S (%); glycolipid and sphingolipid fraction of total lipids; P (%), fraction of phospholipids on total lipids; S/x (%), intracellular polysaccharides on dry biomass; P/x (%), intracellular proteins on dry biomass; TCh/x (%), total chlorophyll (chlorophyll a and b) on dry biomass; TC/x (%), total carotenoids on dry biomass; μ (1/d), maximum specific growth rate; R^2, the R-squared statistical measure; UND, undetermined. (b) ND: not detected, *Others: mainly 10:0, 12:0 an in some cases 18:3(n-6) Note: only glycolipids (i.e., G fraction) are mentioned as the amide bond of sphingolipids resists methanolysis during methyl esterification.

Table S9. (a) Biomass production, reserve materials accumulation and growth parameters and (b) fatty acid composition of total lipids (TL) and their lipid fractions (neutral – N, glycolipids - G and phospholipids - P), of *Nephroselmis pyriformis* PAT2.7 grown in modified Artificial Seawater (mASW) under photoperiod of 24:0 (h light:dark) at pH = 8.5 (Control) or pH = 7.5 in an 8.7 L (V_w = 5 L) capacity Open-Pond Simulating Reactor (OPSR).

a

Growth medium	t (h)	Biomass (x)	Lipids (L)				Polysaccharides (S)	Proteins (P)	Pigments		Growth parameters	
		x (mg/L)	L/x (%)	Lipid fractions (%)			S/x (%)	P/x (%)	TCh/x (%)	TC/x (%)	μ (1/d)	R^2
				N	G+S	P						
**pH = 8.5 (Control)**	240	137.9 ± 32.8	6.7 ± 0.2	UND	UND	UND	11.6 ± 1.1	21.8 ± 1.8	1.4 ± 0.1	0.4 ± 0.0	0.22 ± 0.03	0.95
	450	471.4 ± 27.6	5.5 ± 1.6	41.5 ± 1.5	53.6 ± 3.1	5.0 ± 1.8	15.3 ± 0.9	37.8 ± 3.8	0.8 ± 0.1	0.2 ± 0.0		
**pH = 7.5**	240	88.3 ± 9.3	8.0 ± 1.6	UND	UND	UND	8.7 ± 2.1	42.1 ± 3.0	UND	UND	0.20 ± 0.05	0.91
	450	276.0 ± 30.7	7.6 ± 2.8	63.9 ± 0.3	32.5 ± 0.2	3.6 ± 0.5	11.0 ± 0.0	28.9 ± 0.9	2.8 ± 0.1	0.6 ± 0.0		

b

Growth medium	t (h)	Lipid fraction	Fatty acid composition of total lipids and their fractions (%, wt/wt)							
			14:0	14:1(n-5)	16:0	16:1(n-7)	18:0	18:1(n-9)	18:2(n-6)	Others
**pH = 8.5 (Control)**	240	TL	30.2 ± 0.2	8.3 ± 0.6	9.8 ± 0.0	40.0 ± 4.1	2.6 ± 1.6	5.6 ± 1.7	1.9 ± 0.0	2.4 ± 0.3
	450	TL	31.0 ± 0.7	4.4 ± 1.1	9.4 ± 0.9	39.0 ± 3.9	2.7 ± 2.0	5.4 ± 2.2	0.8 ± 0.3	7.3 ± 1.8
		N	34.6 ± 1.2	5.7 ± 0.4	10.0 ± 0.6	41.1 ± 0.9	0.9 ± 0.0	2.3 ± 0.2	1.3 ± 0.1	7.7 ± 0.4
		G	31.7 ± 0.2	4.7 ± 1.3	8.6 ± 0.8	39.4 ± 0.3	3.3 ± 2.2	3.9 ± 1.4	0.7 ± 0.4	4.1 ± 0.8
		P	14.4 ± 1.8	10.0 ± 0.0	13.7 ± 1.1	29.6 ± 0.4	4.7 ± 0.9	17.1 ± 2.4	5.2 ± 2.8	5.8 ± 0.3
**pH = 7.5**	240	TL	29.1 ± 1.0	3.7 ± 0.6	11.4 ± 0.4	38.6 ± 0.5	1.7 ± 0.8	2.9 ± 1.9	1.2 ± 1.1	11.5 ± 2.5
	450	TL	30.1 ± 0.0	3.4 ± 0.3	11.6 ± 0.2	38.3 ± 0.8	0.9 ± 0.0	3.4 ± 2.3	1.7 ± 1.6	11.1 ± 2.8
		N	32.1 ± 0.1	5.1 ± 0.3	10.3 ± 0.1	43.7 ± 1.5	0.5 ± 0.0	1.8 ± 0.4	<0.5	2.2 ± 0.1
		G	38.3 ± 0.5	8.1 ± 0.5	8.3 ± 0.3	32.8 ± 0.5	0.9 ± 0.5	1.1 ± 0.5	1.7 ± 0.8	5.3 ± 0.3
		P	37.3 ± 0.9	1.9 ± 0.4	25.1 ± 1.1	15.2 ± 1.0	5.9 ± 1.7	9.4 ± 1.8	3.2 ± 0.2	1.9 ± 0.1

Abbreviations: (a) x (mg/L), dry biomass; L/x (%), lipids on dry biomass; N (%); neutral lipid fraction of total lipids; G+S (%); glycolipid and sphingolipid fraction of total lipids; P (%), fraction of phospholipids on total lipids; S/x (%), intracellular polysaccharides on dry biomass; P/x (%), intracellular proteins on dry biomass; TCh/x (%), total chlorophyll (chlorophyll a and b) on dry biomass; TC/x (%), total carotenoids on dry biomass; μ (1/d), maximum specific growth rate; R^2, the R-squared statistical measure; UND, undetermined. (b) ND: not detected, *Others: mainly 10:0, 12:0. Note: only glycolipids (i.e., G fraction) are mentioned as the amide bond of sphingolipids resists methanolysis during methyl esterification.

Table S10. (a) Biomass production, reserve materials accumulation and growth parameters and (b) fatty acid composition of total lipids (TL) and their lipid fractions (neutral – N, glycolipids - G and phospholipids - P), of *Nephroselmis pyriformis* PAT2.7 grown in modified Artificial Seawater (mASW) at pH = 8.5 under photoperiod of 24:0 (h light:dark) or photoperiod 16:8 in an 8.7 L (Vw = 5 L) capacity Open-Pond Simulating Reactor (OPSR).

a

Growth medium	t (h)	Biomass (x)	Lipids (L)				Polysaccharides (S)	Proteins (P)	Pigments		Growth parameters	
		x (mg/L)	L/x (%)	Lipid fractions (%) N	G+S	P	S/x (%)	P/x (%)	TCh/x (%)	TC/x (%)	μ (1/d)	R^2
Photoperiod 24:0 (Control)	240	137.9 ± 32.8	6.7 ± 0.2	UND	UND	UND	11.6 ± 1.1	21.8 ± 1.8	1.4 ± 0.1	0.4 ± 0.0	0.22 ± 0.03	0.95
	450	471.4 ± 27.6	5.5 ± 1.6	41.5 ± 1.5	53.6 ± 3.1	5.0 ± 1.8	15.3 ± 0.9	37.8 ± 3.8	0.8 ± 0.1	0.2 ± 0.0		
Photoperiod 16:8	240	90.0 ± 1.5	3.8 ± 0.1	UND	UND	UND	8.2 ± 0.0	19.0 ± 0.0	UND	UND	0.10 ± 0.04	0.91
	450	191.5 ± 10.4	4.2 ± 0.2	55.0 ± 1.3	42.0 ± 0.9	0.8 ± 0.0	10.2 ± 0.4	22.0 ± 4.7	UND	UND		

b

Growth medium	t (h)	Lipid fraction	Fatty acid composition of total lipids and their fractions (%, wt/wt) 14:0	14:1(n-5)	16:0	16:1(n-7)	18:0	18:1(n-9)	18:2(n-6)	Others
Photoperiod 24:0 (Control)	240	TL	30.2 ± 0.2	8.3 ± 0.6	9.8 ± 0.0	40.0 ± 4.1	2.6 ± 1.6	5.6 ± 1.7	1.9 ± 0.0	2.4 ± 0.3
	450	TL	31.0 ± 0.7	4.4 ± 1.1	9.4 ± 0.9	39.0 ± 3.9	2.7 ± 2.0	5.4 ± 2.2	0.8 ± 0.3	7.3 ± 1.8
		N	34.6 ± 1.2	5.7 ± 0.4	10.0 ± 0.6	41.1 ± 0.9	0.9 ± 0.0	2.3 ± 0.2	1.3 ± 0.1	7.7 ± 0.4
		G	31.7 ± 0.2	4.7 ± 1.3	8.6 ± 0.8	39.4 ± 0.3	3.3 ± 2.2	3.9 ± 1.4	0.7 ± 0.4	4.1 ± 0.8
		P	14.4 ± 1.8	10.0 ± 0.0	13.7 ± 1.1	29.6 ± 0.4	4.7 ± 0.9	17.1 ± 2.4	5.2 ± 2.8	5.8 ± 0.3
Photoperiod 16:8	240	TL	31.1 ± 0.5	3.9 ± 0.3	12.4 ± 0.3	32.5 ± 0.9	3.2 ± 0.7	5.9 ± 0.0	ND	10.9 ± 1.1
	450	TL	24.8 ± 1.2	4.5 ± 1.0	14.7 ± 2.1	28.1 ± 1.0	6.3 ± 1.5	11.4 ± 2.7	5.0 ± 1.6	5.2 ± 2.5
		N	36.0 ± 0.5	3.7 ± 0.1	11.4 ± 0.1	44.0 ± 0.8	0.5 ± 0.1	1.7 ± 0.2	0.5 ± 0.1	1.0 ± 0.2
		G	31.0 ± 2.8	11.1 ± 0.5	10.6 ± 0.2	28.8 ± 0.0	3.0 ± 0.0	6.1 ± 0.8	0.0 ± 0.0	9.4 ± 1.7
		P	13.4 ± 0.3	8.6 ± 0.1	13.6 ± 0.5	18.1 ± 0.1	9.5 ± 0.5	14.4 ± 0.5	9.1 ± 2.6	13.3 ± 0.7

Abbreviations: (a) x (mg/L), dry biomass; L/x (%), lipids on dry biomass; N (%); neutral lipid fraction of total lipids; G+S (%); glycolipid and sphingolipid fraction of total lipids; P (%), fraction of phospholipids on total lipids; S/x (%), intracellular polysaccharides on dry biomass; P/x (%), intracellular proteins on dry biomass; TCh/x (%), total chlorophyll (chlorophyll a and b) on dry biomass; TC/x (%), total carotenoids on dry biomass; μ (1/d), maximum specific growth rate; R^2, the R-squared statistical measure; UND, undetermined. (b) ND: not detected, *Others: mainly 10:0, 12:0. Note: only glycolipids (i.e., G fraction) are mentioned as the amide bond of sphingolipids resists methanolysis during methyl esterification.

Table S11. The data represent the values of biomass (x, mg/L), reserve materials accumulation (i.e., lipids, L/x%, wt/wt, polysaccharides, S/x%, wt/wt and proteins, P/x%, wt/wt), and fatty acid composition of total lipids (left part) when *Picochlorum costavermella* VAS2.5 was cultured under control conditions (modified Artificial Sea Water - mASW, pH = 8.5 and photoperiod 24:0), nitrogen limitation (mASW.N$^-$) and phosphorus limitation (mASW.P$^-$). Two-sample t-test was applied for statistical analysis of the values regarding biomass production and reserve materials accumulation at 240 h and 450 h of the same culture. Statistically significant differences of biomass production, reserve materials accumulation and fatty acid composition of total lipids among the various culture conditions at 240 h and 450 h of culture were identified using one-way ANOVA analysis of variance (right part). Tukey's Honest Significant Difference (HSD) post hoc test was applied to identify pairwise differences between group means. A value of p ≤ 0.05 was considered statistically significant.

***Picochlorum costavermella* VAS2.5**

Two-Sample t-test

	**Control**		**mASW.N$^-$**		**mASW.P$^-$**	
	**240 h**	**450 h**	**240 h**	**450 h**	**240 h**	**450 h**
**x (mg/L)**	113.1	275.8	52.1	254.5	196.3	202.0
	88.3	262.4	50.9	236.7	140.3	201.2
	p =	0.00693	*p* =	0.00211	*p* =	0.35642
**L/x% (wt/wt)**	24.8	16.1	3.8	10.7	6.5	16.1
	21.0	16.1	3.6	10.1	4.7	16.1
	p =	0.0691	*p* =	0.00222	*p* =	0.00736
**S/x% (wt/wt)**	12.2	15.5	8.2	10.0	7.4	1.7
	12.0	14.9	8.2	10.0	7.2	1.3
	p =	0.01025	*p* =	0.00154	*p* =	0.00148
**P/x% (wt/wt)**	51.5	53.5	15.3	25.2	78.2	36.0
	38.7	48.5	15.3	23.8	70.0	35.2
	p =	0.481	*p* =	0.00583	*p* =	0.01126
**Composition of total lipids in fatty acids**						
**14:0**	6.2	5.4	5.3	8.3	7.2	6.8
	4.0	2.6	4.5	7.1	7.2	7.2
**14:1(n-5)**	3.3	4.3	0.5	5.3	2.9	2.8
	3.1	2.3	0.5	4.1	2.7	2.4

One-Way ANOVA (Tukey test)

	**t (h)**		**Control - mASW.N$^-$**	**Control - mASW.P$^-$**	**mASW.N$^-$ - mASW.P$^-$**
**x (mg/L)**	240	*p*	0.26647	0.14236	0.03733
	450		0.15711	0.01036	0.03404
**L/x% (wt/wt)**	240	*p*	0.00316	0.00428	0.57393
	450		0.000398581	1	0.000398581
**S/x% (wt/wt)**	240	*p*	0.000134972	0.0000752793	0.01251
	450		0.000814292	0.0000462736	0.000196824
**P/x% (wt/wt)**	240	*p*	0.03482	0.03728	0.00512
	450		0.00235	0.01136	0.02831
**One-Way ANOVA (Tukey test)**					
**14:0**	240	*p*	0.97633	0.21758	0.18184
	450		0.11718	0.18415	0.85008
**14:1(n-5)**	240	*p*	0.000353018	0.08018	0.000569511
	450		0.42511	0.76775	0.22211

**16:0**	17.3	19.6	22.4	23.9	25.3	26.5		240	*p*	0.01285	0.00379	0.07171
	15.7	14.8	21.6	20.9	24.5	24.9		450		0.22372	0.07605	0.45458
**16:1(n-7)**	28.0	24.6	25.1	31.5	30.1	28.5		240	*p*	0.98438	0.49808	0.57599
	20.0	15.4	24.1	29.5	26.9	25.3		450		0.15653	0.33901	0.68376
**17:0**	0.1	0.1	1.1	0.7	0.3	0.5		240	*p*	0.00328	0.17539	0.00683
	0.1	0.1	0.9	0.5	0.3	0.5		450		0.0178	0.03285	0.51846
**18:0**	1.5	1.9	0.1	6.8	0.8	2.2		240	*p*	0.00683	0.02384	0.10755
	1.5	1.1	0.1	6.2	0.4	0.8		450		0.01164	1	0.01164
**18:1(n-9)**	30.8	42.3	5.8	6	7.1	9.7		240	*p*	0.04591	0.05469	0.95769
	19.6	19.3	4.8	3.8	6.1	7.1		450		0.13947	0.18919	0.9295
**18:2(n-6)**	6.6	9.1	2	1.4	2.3	1.6		240	*p*	0.25401	0.2687	0.9978
	3.0	3.9	1.2	0.8	1.1	1.2		450		0.16542	0.18605	0.98929
**18:3(n-3)**	0.5	0.5	12.7	0.0	0.8	0.0		240	*p*	0.000304297	1	0.000304297
	0.5	0.5	11.7	0.0	0.2	0.0		450		-	-	-
**18:4(n-3)**	0.0	0.0	3.3	0.0	2.3	0.5		240	*p*	0.00241	0.00525	0.12588
	0.0	0.0	2.7	0.0	2.3	0.5		450		-	-	-
**20:1(n-9)**	3.1	2.2	4.5	3.9	6.6	3.9		240	*p*	0.73437	0.53707	0.91994
	1.9	1.6	3.3	2.3	2.6	3.9		450		0.33279	0.12498	0.55434
**20:5(n-3)**	19.6	17.6	14.4	21.9	22.2	20.4		240	*p*	0.76126	0.65453	0.34484
	13.2	8.8	13.4	20.1	17	14.4		450		0.31848	0.65004	0.72029
**Others**	2.0	2.0	10.9	8.5	1.7	8.5		240	*p*	0.00515	0.97143	0.0048
	1.4	1.0	8.9	8.1	1.3	1.7		450		0.1799	0.49371	0.55824

$p>0.05$, not statistically significant

$p<0.05$, statistically significant

Table S12. The data represent the values of biomass (x, mg/L), reserve materials accumulation (i.e., lipids, L/x%, wt/wt, polysaccharides, S/x%, wt/wt and proteins, P/x%, wt/wt), and fatty acid composition of total lipids (left part) when *Picochlorum costavermella* VAS2.5 was cultured under control conditions (modified Artificial Sea Water - mASW, pH = 8.5 and photoperiod 24:0), mASW , pH = 7.5 and photoperiod 24:0 ('pH = 7.5') and mASW, pH = 8.5 and photoperiod 16:8 ('Photoperiod 16:8'). Two-sample t-test was applied for statistical analysis of the values regarding biomass production and reserve materials accumulation at 240 h and 450 h of the same culture. Statistically significant differences of biomass production, reserve materials accumulation and fatty acid composition of total lipids among the experimental sets Control – pH = 7.5 and Control – Photoperiod 16:8, at 240 h and 450 h of culture, were identified using Two-sample t-test as well. A value of $p \leq 0.05$ was considered statistically significant.

***Picochlorum costavermella* VAS2.5**

	**Two-Sample t-test**					
	**Control**		**pH = 7.5**		**Photoperiod 16:8**	
	**240 h**	**450 h**	**240 h**	**450 h**	**240 h**	**450 h**
**x (mg/L)**	113.1	275.8	70	201.8	189.5	268.3
	88.3	262.4	63.6	86.8	69.9	173.1
	p =	0.00693	*p* =	0.31065	*p* =	0.35596
**L/x% (wt/wt)**	24.8	16.1	38.6	38.4	7.6	8.3
	21.0	16.1	14.4	30.4	7.2	6.3
	p =	0.07095	*p* =	0.59854	*p* =	0.93083
**S/x% (wt/wt)**	12.2	15.5	12.5	9.8	8.3	10.9
	12.0	14.9	8.3	9.0	7.7	9.1
	p =	0.01025	*p* =	0.68596	*p* =	0.16955
**P/x% (wt/wt)**	51.5	53.5	53.8	40.6	9.6	36.9
	38.7	48.5	32.2	32.8	9.4	28.3
	p =	0.481	*p* =	0.63831	*p* =	0.03296
**Composition of total lipids in fatty acids**						
**14:0**	6.2	5.4	8.9	10.7	7.6	6.0
	4.0	2.6	7.7	8.7	4.4	4.4
**14:1(n-5)**	3.3	4.3	4.6	3.7	2.1	3.7
	3.1	2.3	3.8	3.1	1.7	1.9

**Two-Sample t-test**				
	**t (h)**		**pH = 8.5 (Control) – pH = 7.5**	**Photoperiod 24:0 (Control) – Photoperiod 16:8**
**x (mg/L)**	240	*p*	0.11798	0.6817
	450		0.16385	0.42001
**L/x% (wt/wt)**	240	*p*	0.79652	0.01485
	450		0.04484	0.01256
**S/x% (wt/wt)**	240	*p*	0.50364	0.0059
	450		0.00735	0.03171
**P/x% (wt/wt)**	240	*p*	0.88253	0.03084
	450		0.09087	0.06593
**Two-Sample t-test**				
**14:0**	240	*p*	0.12517	0.68854
	450		0.08029	0.53431
**14:1(n-5)**	240	*p*	0.13613	0.02833
	450		0.93243	0.74584

**16:0**	17.3	19.6	26.4	14.8	28.2	26.4		240	*p*	0.01309	0.08553
	15.7	14.8	25	13.6	22.8	21.2		450		0.34906	0.20314
**16:1(n-7)**	28.0	24.6	34.6	25.6	31.0	27.5		240	*p*	0.13198	0.57788
	20.0	15.4	33.4	22.8	24.0	25.3		450		0.47451	0.30866
**17:0**	0.1	0.1	0.5	0.4	0.0	0.0		240	*p*	0.02056	0.38794
	0.1	0.1	0.4	0.3	0.1	0.1		450		0.03958	0.38794
**18:0**	1.5	1.9	0.7	1.9	3.3	6.1		240	*p*	0.01686	0.06935
	1.5	1.1	0.5	1.1	2.5	2.9		450		1	0.21053
**18:1(n-9)**	30.8	42.3	6.1	8.2	11.6	16.8		240	*p*	0.07383	0.12783
	19.6	19.3	5.3	5.2	10.4	11.0		450		0.17328	0.29022
**18:2(n-6)**	6.6	9.1	1.9	4.2	4.4	3.0		240	*p*	0.25431	0.49363
	3.0	3.9	2.0	3.8	1.2	2.8		450		0.43887	0.30067
**18:3(n-3)**	0.5	0.5	0.6	3.3	1.3	2.7		240	*p*	0.29289	0.00772
	0.5	0.5	0.5	1.9	1.2	1.3		450		0.09993	0.17479
**20:1(n-9)**	3.1	2.2	1.8	2.8	9.7	7.5		240	*p*	0.27452	0.15963
	1.9	1.6	1.2	2.9	5.3	5.5		450		0.08902	0.04785
**20:5(n-3)**	19.6	17.6	15.3	24.2	10	9.0		240	*p*	0.74381	0.16453
	13.2	8.8	15.1	23.6	8.8	7.0		450		0.13606	0.36829
**Others**	2.0	2.0	2.3	8.6	6.1	4.6		240	*p*	0.21227	0.46083
	1.4	1.0	2.2	5.0	1.5	3.8		450		0.10503	0.0519

$p>0.05$, not statistically significant

$p<0.05$, statistically significant

Table S13. The data represent the values of biomass (x, mg/L), reserve materials accumulation (i.e., lipids, L/x%, wt/wt, polysaccharides, S/x%, wt/wt and proteins, P/x%, wt/wt), and fatty acid composition of total lipids (left part) when *Picochlorum oklahomense* PAT3.2B was cultured under control conditions (modified Artificial Sea Water - mASW, pH = 8.5 and photoperiod 24:0), nitrogen limitation (mASW.N$^-$) and phosphorus limitation (mASW.P$^-$). Two-sample t-test was applied for statistical analysis of the values regarding biomass production and reserve materials accumulation at 240 h and 450 h of the same culture. Statistically significant differences of biomass production, reserve materials accumulation and fatty acid composition of total lipids among the various culture conditions at 240 h and 450 h of culture were identified using one-way ANOVA analysis of variance (right part). Tukey's Honest Significant Difference (HSD) post hoc test was applied to identify pairwise differences between group means. A value of $p \leq 0.05$ was considered statistically significant.

***Picochlorum oklahomense* PAT3.2B**

Two-Sample t-test

	**Control**		**mASW.N$^-$**		**mASW.P$^-$**	
	**240 h**	**450 h**	**240 h**	**450 h**	**240 h**	**450 h**
**x (mg/L)**	109.1	451.9	54.6	102.6	50.6	129.4
	90.9	390.3	44.0	93.4	39.8	121.2
	p =	0.00986	*p* =	0.02014	*p* =	0.00709
**L/x% (wt/wt)**	15.4	11.8	3.8	6.1	10.9	8.5
	12.4	11.2	3.9	4.1	9.9	3.5
	p =	0.25722	*p* =	0.3382	*p* =	0.22652
**S/x% (wt/wt)**	13.7	9.4	8.2	9.5	14.1	14.1
	12.5	8.2	8.2	8.3	11.9	8.9
	p =	0.0368	*p* =	0.33902	*p* =	0.6483
**P/x% (wt/wt)**	59.3	54.0	15.3	33.0	32.1	52.3
	57.5	44.4	15.3	25.2	24.1	39.5
	p =	0.20027	*p* =	0.07097	*p* =	0.14237
**Composition of total lipids in fatty acids**						
**14:0**	5.5	1.9	7.1	8.2	8.1	12.1
	4.1	1.5	6.3	7.6	7.1	8.5

One-Way ANOVA (Tukey test)

	**t (h)**		**Control - mASW.N$^-$**	**Control - mASW.P$^-$**	**mASW.N$^-$ - mASW.P$^-$**
**x (mg/L)**	240	*p*	0.02728	0.02203	0.90841
	450		0.00222	0.00287	0.5939
**L/x% (wt/wt)**	240	*p*	0.00903	0.1416	0.02992
	450		0.12244	0.17006	0.91526
**S/x% (wt/wt)**	240	*p*	0.02631	0.49748	0.02729
	450		0.99889	0.52553	0.54613
**P/x% (wt/wt)**	240	*p*	0.00207	0.00584	0.06215
	450		0.13536	0.89626	0.1971
**One-Way ANOVA (Tukey test)**					
**14:0**	240	*p*	0.17489	0.07221	0.54725
	450		0.05133	0.02134	0.37062

**14:1(n-5)**	3.2	7.5	7.1	8.2	8.1	12.1	240	*p*	0.01858	0.01048	0.46167
	2.2	6.3	6.3	7.6	7.1	8.5	450		0.81169	0.22326	0.39519
**16:0**	15.2	18.9	15.6	15.2	19.5	8.2	240	*p*	0.88734	0.35884	0.54736
	13.4	15.3	14.8	14.6	15.3	8.2	450		0.41477	0.01908	0.04127
**16:1(n-7)**	2.9	3.3	4.7	3.8	3.6	3.1	240	*p*	0.34162	0.46325	0.94011
	1.7	2.7	2.9	3.6	3.4	2.7	450		0.20061	0.94373	0.15246
**17:0**	3.6	0.5	8.0	8.3	3.6	12.4	240	*p*	0.27428	0.92054	0.39103
	2.2	0.5	4.2	6.1	3.6	8.0	450		0.08783	0.03412	0.40838
**18:0**	7.8	8.7	13.6	2.8	8.4	16.9	240	*p*	0.56326	0.86271	0.35182
	7.2	5.9	8.2	2.4	3.4	13.5	450		0.15441	0.04435	0.01232
**18:1(n-9)**	23.3	14.8	25.2	21.4	20.0	25.6	240	*p*	0.77002	0.58621	0.30953
	18.5	14.6	21.4	13.8	14.6	18.6	450		0.78672	0.32277	0.59286
**18:2(n-6)**	4.9	22.2	27.4	27.1	30.3	7.9	240	*p*	0.30493	0.15261	0.72853
	4.7	20.6	10.8	19.1	20.5	7.9	450		0.87192	0.05409	0.03969
**18:3(n-3)**	24.5	23.6	14.4	14.0	16.4	18.3	240	*p*	0.1663	0.1433	0.98208
	19.7	22.4	13.2	13.2	10.0	18.3	450		0.0011	0.00819	0.00871
**18:4(n-3)**	8.5	0.5	0.0	5.2	0.0	0.0	240	*p*	0.45311	0.45311	-
	0.5	0.5	0.0	4.0	0.0	0.0	450		0.00732	0.6156	0.00525
**Others**	3.2	7.5	4.7	5.1	4.6	2.8	240	*p*	0.57882	0.24085	0.63304
	2.2	6.3	2.7	2.7	4.6	2.8	450		0.13857	0.0684	0.64727

$p>0.05$, not statistically significant

$p<0.05$, statistically significant

Table S14 The data represent the values of biomass (x, mg/L), reserve materials accumulation (i.e., lipids, L/x%, wt/wt, polysaccharides, S/x%, wt/wt and proteins, P/x%, wt/wt), and fatty acid composition of total lipids (left part) when *Picochlorum oklahomense* PAT3.2B was cultured under control conditions (modified Artificial Sea Water - mASW, pH = 8.5 and photoperiod 24:0), mASW , pH = 7.5 and photoperiod 24:0 ('pH = 7.5') and mASW, pH = 8.5 and photoperiod 16:8 ('Photoperiod 16:8'). Two-sample t-test was applied for statistical analysis of the values regarding biomass production and reserve materials accumulation at 240 h and 450 h of the same culture. Statistically significant differences of biomass production, reserve materials accumulation and fatty acid composition of total lipids among the experimental sets Control – pH = 7.5 and Control – Photoperiod 16:8, at 240 h and 450 h of culture, were identified using Two-sample t-test as well. A value of $p \leq 0.05$ was considered statistically significant.

***Picochlorum oklahomense* PAT3.2B**

	Two-Sample t-test							Two-Sample t-test			
	Control		pH = 7.5		Photoperiod 16:8						
	240 h	450 h	240 h	450 h	240 h	450 h		t (h)		pH = 8.5 (Control) - pH = 7.5	Photoperiod 24:0 (Control) - Photoperiod 16:8
x (mg/L)	109.1	451.9	55.1	303.6	54.3	191.2		240	*p*	0.04318	0.04519
	90.9	390.3	42.9	178.6	39.7	118		450		0.12284	0.03074
	p =	0.00986	*p* =	0.02799	*p* =	0.01055					
L/x% (wt/wt)	15.4	11.8	35.2	9.6	3.7	1.4		240	*p*	0.45147	0.02115
	12.4	11.2	13.2	6.0	2.7	0.6		450		0.1798	0.00226
	p =	0.25722	*p* =	0.27904	*p* =	0.07527					
S/x% (wt/wt)	13.7	9.4	17.4	23.9	6.7	5.7		240	*p*	0.85829	0.02126
	12.5	8.2	6.6	15.7	4.9	5.5		450		0.11743	0.03429
	p =	0.0368	*p* =	0.36895	*p* =	0.8457					
P/x% (wt/wt)	59.3	54.0	53.8	60.4	7.9	23.9		240	*p*	0.2912	0.000757806
	57.5	44.4	32.2	43.4	5.7	18.3		450		0.80806	0.03695
	p =	0.20027	*p* =	0.58367	*p* =	0.04152					
	Composition of total lipids in fatty acids							Two-Sample t-test			
14:0	5.5	1.9	2.6	2.6	-	5.7		240	*p*	0.09075	-
	4.1	1.5	1.5	1.4	-	3.3		450		0.682	0.14799

**14:1(n-5)**	3.2	7.5	6.5	14.0	-	9.2		240	*p*	0.02665	-
	2.2	6.3	5.9	8.8	-	8.0		450		0.23376	0.18303
**16:0**	15.2	18.9	19.3	15.4	-	14.0		240	*p*	0.09695	-
	13.4	15.3	17.3	8.2	-	13.4		450		0.31855	0.20347
**16:1(n-7)**	2.9	3.3	3.3	4.3	-	2.4		240	*p*	0.58761	-
	1.7	2.7	2.3	2.3	-	2.5		450		0.80088	0.21227
**17:0**	3.6	0.5	5.9	0.0	-	0.0		240	*p*	0.36864	-
	2.2	0.5	3.3	0.1	-	0.1		450		0.02986	0.02986
**18:0**	7.8	8.7	1.2	9.2	-	8.8		240	*p*	0.00306	-
	7.2	5.9	0.8	4.8	-	7.2		450		0.91892	0.70654
**18:1(n-9)**	23.3	14.8	15.6	14.7	-	15.8		240	*p*	0.1364	-
	18.5	14.6	13.2	11.9	-	12.6		450		0.42363	0.78463
**18:2(n-6)**	4.9	22.2	23.9	22.4	-	33.0		240	*p*	0.02197	-
	4.7	20.6	18.9	19.2	-	20.4		450		0.76923	0.49177
**18:3(n-3)**	24.5	23.6	26.3	27.3	-	24.8		240	*p*	0.85787	-
	19.7	22.4	15.5	18.3	-	12.2		450		0.96886	0.55079
**18:4(n-3)**	8.5	0.5	5.8	2.0	-	0.0		240	*p*	0.96691	-
	0.5	0.5	2.8	1.0	-	0.1		450		0.17184	0.02986
**Others**	3.2	7.5	3.8	5.1	-	5.1		240	*p*	0.23663	-
	2.2	6.3	3.4	1.5	-	1.5		450		0.19822	0.19822

$p > 0.05$, not statistically significant

$p < 0.05$, statistically significant

Table S15. The data represent the values of biomass (x, mg/L), reserve materials accumulation (i.e., lipids, L/x%, wt/wt, polysaccharides, S/x%, wt/wt and proteins, P/x%, wt/wt), and fatty acid composition of total lipids (left part) when *Picochlorum oklahomense* SAG4.4 was cultured under control conditions (modified Artificial Sea Water - mASW, pH = 8.5 and photoperiod 24:0), nitrogen limitation (mASW.N$^-$) and phosphorus limitation (mASW.P$^-$). Two-sample t-test was applied for statistical analysis of the values regarding biomass production and reserve materials accumulation at 240 h and 450 h of the same culture. Statistically significant differences of biomass production, reserve materials accumulation and fatty acid composition of total lipids among the various culture conditions at 240 h and 450 h of culture were identified using one-way ANOVA analysis of variance (right part). Tukey's Honest Significant Difference (HSD) post hoc test was applied to identify pairwise differences between group means. A value of $p \leq 0.05$ was considered statistically significant.

***Picochlorum oklahomense* SAG4.4**

Two-Sample t-test

	Control		mASW.N$^-$		mASW.P$^-$	
	**240 h**	**450 h**	**240 h**	**450 h**	**240 h**	**450 h**
**x (mg/L)**	95.6	312.9	86.3	210.1	108.2	581.2
	85.8	285.7	74.5	181.3	83.6	476.8
	p =	0.00477	*p* =	0.01773	*p* =	0.01499
**L/x% (wt/wt)**	10.6	10.9	2.5	1.2	10.6	7.3
	6.6	7.9	1.7	1.2	4.6	4.1
	p =	0.77931	*p* =	0.1695	*p* =	0.6325
**S/x% (wt/wt)**	12.0	13.8	14.6	12.0	14.1	12.1
	10.4	12.4	9.2	9.8	11.9	9.1
	p =	0.21579	*p* =	0.7643	*p* =	0.32601
**P/x% (wt/wt)**	36.9	46.1	12.4	18.2	33.7	39.6
	33.1	41.5	5.4	10.2	25.3	36.8
	p =	0.09828	*p* =	0.42374	*p* =	0.18833
**Composition of total lipids in fatty acids**						
**14:0**	6.0	1.9	4.0	2.5	1.7	3.7
	2.2	1.5	3.2	2.5	0.5	1.5
**14:1(n-5)**	2.8	7.5	9.8	10.2	6.6	12

One-Way ANOVA (Tukey test)

	**t (h)**		**Control - mASW.N$^-$**	**Control - mASW.P$^-$**	**mASW.N$^-$ - mASW.P$^-$**
**x (mg/L)**	240	*p*	0.69231	0.90234	0.48135
	450		0.20386	0.03045	0.01079
**L/x% (wt/wt)**	240	*p*	0.21813	0.94032	0.29418
	450		0.04001	0.2442	0.17034
**S/x% (wt/wt)**	240	*p*	0.95726	0.76537	0.89978
	450		0.46211	0.3915	0.98145
**P/x% (wt/wt)**	240	*p*	0.0237	0.54635	0.04479
	450		0.00996	0.43543	0.01803
**One-Way ANOVA (Tukey test)**					
**14:0**	240	*p*	0.95196	0.30742	0.40367
	450		0.68916	0.63304	0.99342
**14:1(n-5)**	240	*p*	0.20318	0.57851	0.53866

	1.6	6.3	5.4	10.0	3.0	8.2		450		0.26714	0.26714	1
**16:0**	13.5	18.9	19.9	16.1	19.5	18.0		240	*p*	0.65051	1	0.65051
	13.3	15.3	16.7	14.3	7.3	17.4		450		0.5558	0.9322	0.40434
**16:1(n-7)**	1.8	3.3	8.1	5.4	4.9	2.8		240	*p*	0.05668	0.40541	0.17124
	1.6	2.7	5.9	4.0	2.5	2.4		450		0.14931	0.81929	0.09238
**17:0**	2.8	0.5	8.4	7.7	5.5	6.1		240	*p*	0.07658	0.38711	0.26428
	1.6	0.5	6.0	7.5	3.3	5.5		450		0.000217477	0.00052068	0.01235
**18:0**	5.8	8.7	4.3	3.2	0.8	0.6		240	*p*	0.499	0.18033	0.55384
	3.6	6.9	0.9	3.0	0.6	0.6		450		0.01603	0.00472	0.08503
**18:1(n-9)**	19.1	14.8	9.9	17.5	19.6	17.9		240	*p*	0.0159	0.98994	0.01688
	17.7	14.6	8.1	16.5	16.8	12.7		450		0.59454	0.95905	0.73614
**18:2(n-6)**	6.5	22.2	6.2	20.4	22.6	24.3		240	*p*	0.87157	0.00728	0.00873
	2.9	20.6	5.2	19.4	19.6	17.9		450		0.85354	0.99335	0.90187
**18:3(n-6)**	8.6	0.5	0.0	0.0	0.0	0.0		240	*p*	0.00538	0.00538	-
	6.6	0.5	0.0	0.0	0.0	0.0		450		-	-	-
**18:3(n-3)**	24.7	23.6	10.3	14	19.5	22.9		240	*p*	0.20113	0.99474	0.2189
	14.7	22.4	10.3	13	19.1	16.5		450		0.0122	0.99401	0.01857
**18:4(n-3)**	10.9	1.0	0.0	0.0	3.6	3.9		240	*p*	0.04702	0.14647	0.36742
	6.1	1.0	0.0	0.0	2.8	0.9		450		0.72069	0.57612	0.27882
**Others**	14.5	4.7	38.7	6.9	6.2	8.9		240	*p*	0.0345	0.30699	0.0143
	11.1	4.1	28.9	5.9	3.6	3.9		450		0.64914	0.64914	1

$p>0.05$, not statistically significant

$p<0.05$, statistically significant

Table S16 The data represent the values of biomass (x, mg/L), reserve materials accumulation (i.e., lipids, L/x%, wt/wt, polysaccharides, S/x%, wt/wt and proteins, P/x%, wt/wt), and fatty acid composition of total lipids (left part) when *Picochlorum oklahomense* SAG4.4 was cultured under control conditions (modified Artificial Sea Water - mASW, pH = 8.5 and photoperiod 24:0), mASW , pH = 7.5 and photoperiod 24:0 ('pH = 7.5') and mASW, pH = 8.5 and photoperiod 16:8 ('Photoperiod 16:8'). Two-sample t-test was applied for statistical analysis of the values regarding biomass production and reserve materials accumulation at 240 h and 450 h of the same culture. Statistically significant differences of biomass production, reserve materials accumulation and fatty acid composition of total lipids among the experimental sets Control – pH = 7.5 and Control – Photoperiod 16:8, at 240 h and 450 h of culture, were identified using Two-sample t-test as well. A value of $p \leq 0.05$ was considered statistically significant.

Picochlorum oklahomense **SAG4.4**

	**Two-Sample t-test**					
	**Control**		**pH = 7.5**		**Photoperiod 16:8**	
	**240 h**	**450 h**	**240 h**	**450 h**	**240 h**	**450 h**
**x (mg/L)**	95.6	312.9	77.0	217.5	65.2	199.7
	85.8	285.7	76.2	214.9	59.6	199.7
	p =	0.00477	*p* =	0.0000949159	*p* =	0.000416063
**L/x% (wt/wt)**	10.6	10.9	15.2	11.9	2.6	1.7
	6.6	7.9	14.0	11.7	1.0	1.1
	p =	0.77931	*p* =	0.0441	*p* =	0.68573
**S/x% (wt/wt)**	12.0	13.8	14.1	12.8	6.3	8.6
	10.4	12.4	12.3	12.8	6.1	7.2
	p =	0.21579	*p* =	0.73524	*p* =	0.13807
**P/x% (wt/wt)**	36.9	46.1	49.1	46.2	9.7	24.0
	33.1	41.5	45.7	35.2	9.5	23.0
	p =	0.09828	*p* =	0.36455	*p* =	0.00134
**Composition of total lipids in fatty acids**						
**14:0**	6.0	1.9	0.7	1.8	6.4	6.3

**Two-Sample t-test**			
**t (h)**		**pH = 8.5 (Control) - pH = 7.5**	**Photoperiod 24:0 (Control) - Photoperiod 16:8**
240	*p*	0.10311	0.03754
450		0.02598	0.01812
240	*p*	0.10278	0.08739
450		0.25146	0.03467
240	*p*	0.23861	0.02503
450		0.75573	0.03438
240	*p*	0.03977	0.00556
450		0.65489	0.01318
**Two-Sample t-test**			
240	*p*	0.2002	0.36702

	2.2	1.5	0.3	1.6	6.2	4.1		450		1	0.08868
**14:1(n-5)**	2.8	7.5	1.5	6.6	4.5	7.0		240	*p*	0.2907	0.14768
	1.6	6.3	1.1	6.0	3.5	4.0		450		0.46548	0.47752
**16:0**	13.5	18.9	13.5	21.8	7.1	7.1		240	*p*	0.46084	0.00377
	13.3	15.3	11.3	21.0	6.3	6.7		450		0.14495	0.03011
**16:1(n-7)**	1.8	3.3	13.5	5.5	9.4	9.4		240	*p*	0.00294	0.25788
	1.6	2.7	12.3	5.6	3.4	6.4		450		0.01393	0.08519
**17:0**	2.8	0.5	1.6	5.0	14.5	11.6		240	*p*	0.36883	0.08656
	1.6	0.5	1.4	5.1	8.7	11.7		450		0.000246822	0.0000405787
**18:0**	5.8	8.7	6.8	0.7	14.7	10.8		240	*p*	0.23134	0.38674
	3.6	6.9	6.4	0.8	5.3	10.0		450		0.01596	0.11852
**18:1(n-9)**	19.1	14.8	51.6	14.4	24.7	23.1		240	*p*	0.000798846	0.91263
	17.7	14.6	50.4	14.2	13.5	19.1		450		0.10557	0.08553
**18:2(n-6)**	6.5	22.2	5.0	20.2	15.0	11.0		240	*p*	0.92179	0.28744
	2.9	20.6	4.8	18.6	7.0	9.0		450		0.21913	0.01239
**18:3(n-6)**	8.6	0.5	0.0	0.0	0.0	0.0		240	*p*	-	-
	6.6	0.5	0.0	0.0	0.0	0.0		450		-	-
**18:3(n-3)**	24.7	23.6	5.6	13.8	11	9.2		240	*p*	0.09869	0.18467
	14.7	22.4	4.0	13.4	6.8	9.0		450		0.0045	0.00191
**18:4(n-3)**	10.9	1.0	0.0	0.7	0.0	0.0		240	*p*	-	-
	6.1	1.0	0.0	0.5	0.0	0.0		450		0.05654	-
**20:1(n-9)**	0.0	1.3	0.0	1.3	0.0	0.0		240	*p*	-	-
	0.0	1.1	0.0	1.1	0.0	0.0		450		1	-
**20:5(n-3)**	0.0	0.0	0.0	3.5	0.0	0.0		240	*p*	-	-
	0.0	0.0	0.0	2.1	0.0	0.0		450		-	-
**Others**	14.5	4.7	4.4	7.6	16.7	16.1		240	*p*	0.03741	0.20866
	11.1	4.1	4.0	7.4	15.5	8.9		450		0.01025	0.15418

$p > 0.05$, not statistically significant

$p < 0.05$, statistically significant

Table S17. The data represent the values of biomass (x, mg/L), reserve materials accumulation (i.e., lipids, L/x%, wt/wt, polysaccharides, S/x%, wt/wt and proteins, P/x%, wt/wt), and fatty acid composition of total lipids (left part) when *Microchloropsis gaditana* VON5.3 was cultured under control conditions (modified Artificial Sea Water - mASW, pH = 8.5 and photoperiod 24:0), nitrogen limitation (mASW.N^{-}) and phosphorus limitation (mASW.P^{-}). Two-sample t-test was applied for statistical analysis of the values regarding biomass production and reserve materials accumulation at 240 h and 450 h of the same culture. Statistically significant differences of biomass production, reserve materials accumulation and fatty acid composition of total lipids among the various culture conditions at 240 h and 450 h of culture were identified using one-way ANOVA analysis of variance (right part). Tukey's Honest Significant Difference (HSD) post hoc test was applied to identify pairwise differences between group means. A value of $p \leq 0.05$ was considered statistically significant.

Microchloropsis gaditana **VON5.3**

Two-Sample t-test

	**Control**		**mASW.N^{-}**		**mASW.P^{-}**	
	**240 h**	**450 h**	**240 h**	**450 h**	**240 h**	**450 h**
**x (mg/L)**	145.8	659.0	122	416	221.7	430.2
	72.8	387.0	72.8	371.8	219.7	354.2
	p =	0.09896	*p* =	0.01221	*p* =	0.04578
**L/x% (wt/wt)**	13.4	15.3	10.9	12.2	12.8	21.4
	8.0	8.3	9.3	11.6	7.6	17.0
	p =	0.8267	*p* =	0.16972	*p* =	0.11832
**S/x% (wt/wt)**	7.4	10.1	9.8	10.9	7.5	10.1
	6.6	9.3	7.6	10.5	7.1	9.3
	p =	0.0412	*p* =	0.21554	*p* =	0.03301
**P/x% (wt/wt)**	19.2	26.8	13.7	18.2	21.6	38.6
	18.2	17.0	11.3	14.2	12.6	31.6
	p =	0.58255	*p* =	0.25355	*p* =	0.08736
**Composition of total lipids in fatty acids**						
**14:0**	8.1	7.5	7.1	8.1	8.3	7.0

One-Way ANOVA (Tukey test)

	**t (h)**		**Control - mASW.N^{-}**	**Control - mASW.P^{-}**	**mASW.N^{-} - mASW.P^{-}**
x	240	*p*	0.94251	0.10459	0.08214
	450		0.57362	0.56664	0.99988
L/x%	240	*p*	0.98007	0.98609	0.99944
	450		0.99952	0.21963	0.22537
S/x%	240	*p*	0.32293	0.94946	0.42708
	450		0.24953	1	0.24953
P/x%	240	*p*	0.36399	0.91077	0.5284
	450		0.57651	0.16159	0.07058
**One-Way ANOVA (Tukey test)**					
14:0	240	*p*	0.9911	0.99776	0.98017

	5.7	5.7	7.1	7.1	5.3	6.8	450		0.537	0.93429	0.71383
**14:1(n-5)**	2.9	4.7	3.7	3.7	3.8	5.0	240	*p*	0.25161	0.25161	1
	0.9	2.9	3.5	3.7	3.4	4.6	450		0.99035	0.47343	0.42051
**16:0**	23.2	23.2	24.8	24.8	23.1	20.8	240	*p*	0.83636	0.8838	0.99411
	23.2	22.4	18.2	21.2	20.5	19.0	450		0.99219	0.32954	0.29589
**16:1(n-7)**	32.1	32.1	30.8	27.2	28.7	29.5	240	*p*	0.9981	0.9981	1
	24.9	30.5	26.6	26.6	28.7	28.7	450		0.0216	0.12605	0.12605
**17:0**	0.5	0.3	0.2	0.2	0.2	0.2	240	*p*	0.04807	0.04807	1
	0.5	0.3	0.2	0.2	0.2	0.2	450		0.43863	0.12882	0.43863
**18:0**	1.2	0.5	0.5	0.5	0.4	0.4	240	*p*	0.46002	0.64996	0.91472
	0.4	0.3	0.1	0.1	0.4	0.2	450		0.87655	0.87655	1
**18:1(n-9)**	7.7	7.7	5.9	14.1	11.3	8.4	240	*p*	0.83316	0.99398	0.78111
	7.3	5.9	5.7	5.9	4.3	4.4	450		0.70652	0.99391	0.65217
**18:2(n-6)**	2.5	2.5	2.9	1.6	3.5	2.4	240	*p*	0.95206	0.72739	0.87581
	1.3	1.5	1.5	1.2	1.9	1.2	450		0.67103	0.9512	0.82639
**18:3(n-3)**	2.6	0.9	0.0	0.5	0.5	1.0	240	*p*	0.14821	0.23225	0.86276
	1.0	0.5	0.0	0.5	0.3	0.4	450		0.87447	1	0.87447
**18:4(n-3)**	0.1	0.1	0.0	0.0	1.0	0.6	240	*p*	0.62593	0.01003	0.007
	0.2	0.2	0.1	0.1	0.8	0.6	450		0.32923	0.00899	0.00503
**20:1(n-9)**	4.5	3.3	2.8	3.0	3.4	4.4	240	*p*	0.78944	0.76266	0.99848
	2.5	2.5	2.8	2.8	2.2	4.2	450		1	0.0545	0.0545
**20:5(n-3)**	19.0	21	24.6	24.5	22.0	25.8	240	*p*	0.64459	0.68958	0.99579
	16.2	19	17.2	17.1	19.2	20.0	450		0.97752	0.76034	0.86072
**Others**	9.6	2.8	9.7	5.0	5.0	3.2	240	*p*	0.79803	0.74953	0.43444
	2.8	2.2	6.9	1.4	2.6	1.4	450		0.90959	0.99206	0.85768

$p>0.05$, not statistically significant

$p<0.05$, statistically significant

Table S18. The data represent the values of biomass (x, mg/L), reserve materials accumulation (i.e., lipids, L/x%, wt/wt, polysaccharides, S/x%, wt/wt and proteins, P/x%, wt/wt), and fatty acid composition of total lipids (left part) when *Microchloropsis gaditana* VON5.3 was cultured under control conditions (modified Artificial Sea Water - mASW, pH = 8.5 and photoperiod 24:0), mASW , pH = 7.5 and photoperiod 24:0 ('pH = 7.5') and mASW, pH = 8.5 and photoperiod 16:8 ('Photoperiod 16:8'). Two-sample t-test was applied for statistical analysis of the values regarding biomass production and reserve materials accumulation at 240 h and 450 h of the same culture. Statistically significant differences of biomass production, reserve materials accumulation and fatty acid composition of total lipids among the experimental sets Control – pH = 7.5 and Control – Photoperiod 16:8, at 240 h and 450 h of culture, were identified using Two-sample t-test as well. A value of p ≤ 0.05 was considered statistically significant.

Microchloropsis gaditana VON5.3										
	Two-Sample t-test						Two-Sample t-test			
	Control		pH = 7.5		Photoperiod 16:8					
	240 h	450 h	240 h	450 h	240 h	450 h	t (h)		pH = 8.5 (Control) - pH = 7.5	Photoperiod 24:0 (Control) - Photoperiod 16:8
**x (mg/L)**	145.8	659	84.5	486.8	113.6	189	240	*p*	0.41088	0.9431
	72.8	387	51.5	392.4	99.0	165	450		0.62092	0.12676
	p =	0.09896	*p* =	0.01763	*p* =	0.03728				
**L/x% (wt/wt)**	13.4	15.3	15.3	14.9	4.6	12.7	240	*p*	0.78925	0.14544
	8.0	8.3	8.7	13.3	4.2	11.1	450		0.58737	0.98031
	p =	0.8267	*p* =	0.59933	*p* =	0.01187				
**S/x% (wt/wt)**	7.4	10.1	10.8	11.6	5.1	7.9	240	*p*	0.50986	0.0555
	6.6	9.3	6.6	9.4	4.3	6.3	450		0.56486	0.10077
	p =	0.0412	*p* =	0.52697	*p* =	0.11535				
**P/x% (wt/wt)**	19.2	26.8	63.0	37.1	9.3	29.3	240	*p*	0.2221	0.00313
	18.2	17.0	30.8	27.1	8.9	17.9	450		0.28244	0.84208
	p =	0.58255	*p* =	0.47259	*p* =	0.12611				
**Composition of total lipids in fatty acids**							**Two-Sample t-test**			
**14:0**	8.1	7.5	7.5	7.5	3.3	12.8	240	*p*	0.83097	0.0916
	5.7	5.7	6.9	7.4	3.1	10.8	450		0.44523	0.06089
**14:1(n-5)**	2.9	4.7	3.8	5.1	2.4	8.2	240	*p*	0.2651	0.72909
	0.9	2.9	3.2	3.1	2.2	6.8	450		0.84425	0.08327
**16:0**	23.2	23.2	22.8	19.7	22.5	20.9	240	*p*	0.32877	0.10405

	23.2	22.4	19.6	18.1	21.7	18.9		450		0.04878	0.11468
**16:1(n-7)**	32.1	32.1	28.5	29.5	12.6	27.2		240	*p*	0.69849	0.04792
	24.9	30.5	24.9	24.9	12.7	24.6		450		0.23428	0.07145
**17:0**	0.5	0.3	0	0.3	0	0.7		240	*p*	-	-
	0.5	0.3	0	0.2	0	0.5		450		1	0.08868
**18:0**	1.2	0.5	0	0.4	4.5	1.4		240	*p*	-	0.05008
	0.4	0.3	0	0.2	3.3	1		450		0.55279	0.07002
**18:1(n-9)**	7.7	7.7	7.9	8	37.1	6.3		240	*p*	0.55279	0.00103
	7.3	5.9	7.5	4.6	35.3	5.1		450		0.81922	0.41618
**18:2(n-6)**	2.5	2.5	3.1	3.1	10.9	3.2		240	*p*	0.31175	0.0206
	1.3	1.5	2.5	1.9	8.9	2.4		450		0.58761	0.33792
**18:3(n-3)**	2.6	0.9	2.8	2.8	2	0		240	*p*	0.38041	0.78463
	1	0.5	2.6	1.4	2.1	0		450		0.19439	-
**18:4(n-3)**	0.1	0.1	0	0.5	0	3.4		240	*p*	-	-
	0.1	0.1	0	0.1	0	2		450		0.43251	0.06568
**20:1(n-9)**	4.5	3.3	0	3.7	1.4	2.4		240	*p*	-	0.1772
	2.5	2.5	0	2.9	1.5	2.2		450		0.55279	0.28286
**20:5(n-3)**	19	21	22.8	25.9	6.6	16.2		240	*p*	0.19494	0.0163
	16.2	19	20	22.9	5.6	16		450		0.13476	0.06045
**Others**	9.6	2.8	8.2	4.6	3.1	3.8		240	*p*	0.88538	0.4348
	2.8	2.2	5.4	2.6	2.7	3		450		0.40256	0.21367

$p>0.05$, not statistically significant

$p<0.05$, statistically significant

Table S19. The data represent the values of biomass (x, mg/L), reserve materials accumulation (i.e., lipids, L/x%, wt/wt, polysaccharides, S/x%, wt/wt and proteins, P/x%, wt/wt), and fatty acid composition of total lipids (left part) when *Nephroselmis pyriformis* PAT2.7 was cultured under control conditions (modified Artificial Sea Water - mASW, pH = 8.5 and photoperiod 24:0), nitrogen limitation (mASW.N$^-$) and phosphorus limitation (mASW.P$^-$). Two-sample t-test was applied for statistical analysis of the values regarding biomass production and reserve materials accumulation at 240 h and 450 h of the same culture. Statistically significant differences of biomass production, reserve materials accumulation and fatty acid composition of total lipids among the various culture conditions at 240 h and 450 h of culture were identified using one-way ANOVA analysis of variance (right part). Tukey's Honest Significant Difference (HSD) post hoc test was applied to identify pairwise differences between group means. A value of p ≤ 0.05 was considered statistically significant.

***Nephroselmis pyriformis* PAT2.7**

Two-Sample t-test

	**Control**		**mASW.N$^-$**		**mASW.P$^-$**	
	**240 h**	**450 h**	**240 h**	**450 h**	**240 h**	**450 h**
**x (mg/L)**	170.7	499.0	137.4	215.0	394.2	473.2
	105.1	443.8	106.7	109.0	379.0	431.2
	p =	0.01612	*p* =	0.54429	*p* =	0.09899
**L/x% (wt/wt)**	6.9	7.1	3.7	7.8	2.5	1.8
	6.5	3.9	3.5	4.6	2.5	1.6
	p =	0.53431	*p* =	0.2463	*p* =	0.02151
**S/x% (wt/wt)**	12.7	16.2	12.5	34	16.2	22.5
	10.5	14.4	11.3	29.2	16.2	21.1
	p =	0.12129	*p* =	0.01541	*p* =	0.01562
**P/x% (wt/wt)**	23.6	41.6	27.4	26.8	12.0	22.4
	20.0	34.0	23.0	19.4	12.0	21.8
	p =	0.06264	*p* =	0.6739	*p* =	0.000914563
**Composition of total lipids in fatty acids**						
**14:0**	30.4	31.7	33.5	32.2	27.1	24.1
	30.0	29.3	30.9	24.6	26.5	22.5
**14:1(n-5)**	8.9	5.5	4.8	4.5	3.7	7.8

One-Way ANOVA (Tukey test)

	**t (h)**		**Control - mASW.N$^-$**	**Control - mASW.P$^-$**	**mASW.N$^-$ - mASW.P$^-$**
**x (mg/L)**	240	*p*	0.86552	0.00768	0.00642
	450		0.019	0.92858	0.02269
**L/x% (wt/wt)**	240	*p*	0.000984724	0.000384794	0.01761
	450		0.92594	0.24656	0.17779
**S/x% (wt/wt)**	240	*p*	0.95448	0.04021	0.04789
	450		0.00998	0.11308	0.04078
**P/x% (wt/wt)**	240	*p*	0.41921	0.04934	0.0221
	450		0.08456	0.07197	0.9714
**One-Way ANOVA (Tukey test)**					
**14:0**	240	*p*	0.30529	0.10556	0.03279
	450		0.81406	0.22291	0.39285
**14:1(n-5)**	240	*p*	0.01413	0.00725	0.32404

	7.7	3.3	4.2	3.3	3.3	7.2		450		0.88731	0.11745	0.08259
**16:0**	9.8	10.3	12.0	14.8	11.1	10.1		240	*p*	0.02591	0.07475	0.31463
	9.8	8.5	11.2	11.6	10.9	9.7		450		0.16532	0.94232	0.21923
**16:1(n-7)**	44.1	42.9	41.8	43.0	35.3	41.7		240	*p*	0.94328	0.53084	0.69312
	35.9	35.1	35.4	41.2	34.5	39.1		450		0.67581	0.91503	0.87886
**18:0**	4.2	4.7	1.6	3.5	3.0	2.3		240	*p*	0.67771	0.97676	0.7848
	1.0	0.7	1.0	1.3	1.6	1.1		450		0.98679	0.86809	0.93145
**18:1(n-9)**	7.3	7.6	2.4	6.6	9.6	9.8		240	*p*	0.18518	0.18518	0.03528
	3.9	3.2	1.8	2.2	8.6	5.0		450		0.94875	0.8187	0.65891
**18:2(n-6)**	1.9	1.1	0.0	0.1	3.9	1.1		240	*p*	0.23372	0.62489	0.10314
	1.9	0.5	0.0	0.1	1.7	1.1		450		0.157	0.44792	0.05691
**Others**	2.7	9.1	12.9	12.9	9.8	9.5		240	*p*	0.13382	0.14668	0.99284
	2.1	5.5	6.5	6.5	9.6	9.3		450		0.72863	0.78017	0.99451

$p>0.05$, not statistically significant

$p<0.05$, statistically significant

Table S20. The data represent the values of biomass (x, mg/L), reserve materials accumulation (i.e., lipids, L/x%, wt/wt, polysaccharides, S/x%, wt/wt and proteins, P/x%, wt/wt), and fatty acid composition of total lipids (left part) when *Nephroselmis pyriformis* PAT2.7 was cultured under control conditions (modified Artificial Sea Water - mASW, pH = 8.5 and photoperiod 24:0), mASW , pH = 7.5 and photoperiod 24:0 ('pH = 7.5') and mASW, pH = 8.5 and photoperiod 16:8 ('Photoperiod 16:8'). Two-sample t-test was applied for statistical analysis of the values regarding biomass production and reserve materials accumulation at 240 h and 450 h of the same culture. Statistically significant differences of biomass production, reserve materials accumulation and fatty acid composition of total lipids among the experimental sets Control – pH = 7.5 and Control – Photoperiod 16:8, at 240 h and 450 h of culture, were identified using Two-sample t-test as well. A value of p ≤ 0.05 was considered statistically significant.

***Nephroselmis pyriformis* PAT2.7**

	**Two-Sample t-test**					
	**Control**		**pH = 7.5**		**Photoperiod 16:8**	
	**240 h**	**450 h**	**240 h**	**450 h**	**240 h**	**450 h**
**x (mg/L)**	170.7	499.0	97.6	306.7	91.5	201.9
	105.1	443.8	79.0	245.3	88.5	181.1
	p =	0.01612	*p* =	0.02799	*p* =	0.01055
**L/x% (wt/wt)**	6.9	7.1	9.6	10.4	3.9	4.4
	6.5	3.9	6.4	4.8	3.7	4.0
	p =	0.53431	*p* =	0.91263	*p* =	0.21554
**S/x% (wt/wt)**	12.7	16.2	10.8	11.0	8.2	10.6
	10.5	14.4	6.6	11.0	8.2	9.8
	p =	0.12129	*p* =	0.37959	*p* =	0.04018
**P/x% (wt/wt)**	23.6	41.6	45.1	29.8	19.0	26.7
	20.0	34.0	39.1	28.0	19.0	17.3
	p =	0.06264	*p* =	0.05195	*p* =	0.59436
**Composition of total lipids in fatty acids**						
**14:0**	30.4	31.7	30.1	30.1	31.6	26.0
	30.0	29.3	28.1	30.0	30.6	23.6

**Two-Sample t-test**			
**t (h)**		**pH = 8.5 (Control) - pH = 7.5**	**Photoperiod 24:0 (Control) - Photoperiod 16:8**
240	*p*	0.28295	0.282
450		0.04185	0.01092
240	*p*	0.50474	0.00589
450		0.58175	0.50474
240	*p*	0.34579	0.09319
450		0.04216	0.03533
240	*p*	0.02844	0.26628
450		0.1503	0.12046
**Two-Sample t-test**			
240	*p*	0.39355	0.23663
450		0.7439	0.07836

**14:1(n-5)**	8.9	5.5	4.3	3.7	4.2	5.5		240	*p*	0.03238	0.02246
	7.7	3.3	3.1	3.1	3.6	3.5		450		0.47295	0.95249
**16:0**	9.8	10.3	11.8	11.8	12.7	16.8		240	*p*	0.06147	0.01393
	9.8	8.5	11.0	11.4	12.1	12.6		450		0.13973	0.14616
**16:1(n-7)**	44.1	42.9	39.1	39.1	33.4	29.1		240	*p*	0.76693	0.21589
	35.9	35.1	38.1	37.5	31.6	27.1		450		0.87662	0.11365
**18:0**	4.2	4.7	2.5	0.9	3.9	7.8		240	*p*	0.66482	0.76393
	1.0	0.7	0.9	1.0	2.5	4.8		450		0.47397	0.28653
**18:1(n-9)**	7.3	7.6	4.8	5.7	5.9	14.1		240	*p*	0.40059	0.89662
	3.9	3.2	1.0	1.1	5.8	8.7		450		0.59395	0.22708
**18:2(n-6)**	1.9	1.1	2.3	3.3	0.0	6.6		240	*p*	0.71828	-
	1.9	0.5	1.1	0.1	0.0	3.4		450		0.6359	0.12309
**Others**	2.7	9.1	14	13.9	12	7.7		240	*p*	0.06876	0.01752
	2.1	5.5	9.0	8.3	9.8	2.7		450		0.37188	0.56579

$p > 0.05$, not statistically significant

$p < 0.05$, statistically significant
